# Global ischemic heart disease burden attributable to environmental risk factors, 1990–2021: an Age-Period-Cohort analysis

**DOI:** 10.3389/fpubh.2025.1622108

**Published:** 2025-08-01

**Authors:** Rui Su, Wangchu Ze, Ruiyu Huang, Yanxia Guo, Gang Liu, Baolu Zhang

**Affiliations:** ^1^Department of Nursing, The Affiliated Hospital, Southwest Medical University, Luzhou, China; ^2^School of Nursing, Southwest Medical University, Luzhou, China; ^3^School of Continuing Education, Guiyang Healthcare Vocational University, Guiyang, China; ^4^Faculty of Nursing and Midwifery, Jiangsu College of Nursing, Huaian, China; ^5^Department of Orthopedics and Center for Orthopedic Diseases Research, Affiliated Traditional Chinese Medicine Hospital of Southwest Medical University, Luzhou, China

**Keywords:** ischemic heart disease, environmental factors, global burden of disease, Age-Period-Cohort analysis, socio-demographic index

## Abstract

**Background:**

Ischemic heart disease (IHD) is the leading cause of global deaths. Environmental exposures contribute substantially to IHD burden, yet their combined effects across socio-demographic strata remain poorly characterized.

**Objective:**

This study aimed to systematically evaluate the global burden of IHD attributable to environmental factors, analyzing its temporal trends, geographical patterns, and Age-Period-Cohort (APC) effects across different socio-demographic index regions from 1990 to 2021.

**Methods:**

Data for this study were obtained from the Global Burden of Disease 2021 (GBD 2021) public dataset to investigate age-standardized deaths rates and disability-adjusted life years (DALYs) rates of IHD attributable to environmental factors from 1990 to 2021. Environmental factors included particulate matter pollution, non-optimal temperature, and lead exposure. Countries were categorized into five socio-demographic index (SDI) levels. The APC analysis model was employed to disentangle age, period, and cohort effects. Data processing and visualization were conducted using R version 4.4.3.

**Results:**

Between 1990 and 2021, global environmental IHD deaths rates decreased by 31.13% and DALYs rates by 29.85%. High SDI regions achieved 70.39% reduction in deaths rates, while low SDI regions showed only 3.13% decrease. Particulate matter pollution remained the predominant environmental contributor with the highest burdens in South Asia, the Middle East, and North Africa. APC analysis revealed that environmental-related IHD burden increased exponentially with age, with earlier birth cohorts showing substantially higher Risk Ratios (RR). Males consistently demonstrated higher burden than females across all environmental factors.

**Conclusion:**

IHD burden attributable to environmental factors shows a declining trend globally but with notable regional and gender disparities. Policymakers in low SDI regions should integrate environmental health into development strategies, high-pollution burden regions should strengthen air quality monitoring and emission control, climate-sensitive regions need to implement temperature adaptation planning, and historically industrialized regions should enhance lead exposure monitoring while ensuring occupational protection for males and environmental health safeguards for the older adults.

## 1 Introduction

Ischemic heart disease (IHD) remains the leading cause of deaths globally. The Global Burden of Disease (GBD) Study 2021 reported that IHD caused 9.0 million deaths, representing 13.2% of total global deaths, and contributed to 188.4 million disability-adjusted life years (DALYs), accounting for 6.5% of global DALYs (1). IHD is projected to primarily contribute to the global disease burden in 2050 (1). Significant disparities exist across regions with different sociodemographic index (SDI) levels, where low and low-middle SDI regions bear a higher IHD burden and experience faster growth in disease rates. In contrast, high SDI regions demonstrate declining or relatively stable IHD indicators. Gender differences are also pronounced, with males showing significantly higher deaths and DALYs than females globally and across all SDI regions. People over 70 years old account for a significant part of the IHD burden, reflecting the profound impact of population aging on cardiovascular disease patterns (1).

Environmental risk factors significantly impact IHD development through multiple pathophysiological mechanisms, underscoring their importance as intervention targets (2–4). Particulate matter pollution (PM2.5 and PM10) has emerged as a primary determinant of persistent IHD risk globally (5), Each 10 μg/m^3^ increase in PM2.5 and PM10 concentrations is associated with 1.16 and 1.80% increases in IHD deaths, respectively. Reducing particulate matter to the WHO air quality guidelines could potentially prevent up to 4.30% of related IHD deaths (6). Non-optimal temperature poses a substantial threat to IHD, with a 1°C temperature decrease increasing IHD-related deaths by 1.6% (7) and a 1°C increase raising the risk of deaths by 0.686% (8). Additionally, lead exposure caused approximately 0.85 million cardiovascular disease deaths in 2019, with IHD accounting for 48.7% of lead-exposure-related deaths (9). Lead exposure may increase IHD risk through mechanisms such as blood pressure alterations and lipid metabolism changes (9). These findings underscore the critical importance of environmental risk factors in IHD development and highlight the urgent need for targeted research and intervention strategies.

Age-Period-Cohort (APC) analyses of IHD have predominantly focused on overall epidemiological patterns (10, 11). While some studies have examined the relationship between particulate matter pollution and IHD burden (12, 13), and regional research has explored associations between non-optimal temperature and IHD (14), comprehensive APC analyses integrating these three key environmental factors across different SDI regions remain absent. This research gap limits our understanding of how environmental factors differentially impact IHD burden across various development levels and hinders the formulation of targeted environmental health policies.

Therefore, this study aims to comprehensively analyze the global burden of IHD attributable to three environmental factors including particulate matter pollution, non-optimal temperature, and lead exposure from 1990 to 2021, examining geographical variations across SDI regions, subsequently decomposing temporal trends using APC analysis to identify age-specific vulnerabilities and period-related changes, and ultimately providing evidence for environmental health policies tailored to different developmental contexts.

## 2 Methods

### 2.1 Data source

This study was conducted following the Strengthening the Reporting of Observational Studies in Epidemiology guidelines (STROBE) for ecological studies and Guidelines for Accurate and Transparent Health Estimates Reporting (GATHER) statement for global health estimates (Supplementary Tables 1, 2), and adheres to the GBD 2021 methodological framework for comparative risk assessment and burden estimation (15). We utilized data from the GBD 2021, a comprehensive database that provides standardized epidemiological estimates for 369 diseases and 87 risk factors across 204 countries and territories (15). We extracted the annual numbers and age-standardized rates of IHD-related deaths rates and disability-adjusted life years (DALYs) rates from 1990 to 2021 by sex, age, and country through the Global Health Data Exchange (GHDx) query tool (16).

Based on the 2021 SDI, we categorized countries and territories into five quintiles: high (>0.81), high-middle (0.70–0.81), middle (0.61–0.69), low-middle (0.46–0.60), and low (<0.46). The SDI is a composite measure of development status based on income per capita, education, and fertility rates (15). Detailed information about the SDI classification of all countries and territories included in this study is provided in Supplementary Tables 3, 4.

For the APC analysis, we divided age and period by 5-year intervals, including 16 age groups (15–19 to 90–94). Following the GBD 2021 study methodology, we primarily analyzed IHD burden in populations aged 15 and above. We divided the period into 7 groups, from 1990 to 1994 to 2020–2021, with the last group covering a 2-year interval. We calculated birth cohorts using the formula (birth cohort: period-age), generating 23 birth cohorts (1895–1899 to 2005–2009). We selected 2000–2004 as the reference period, 40–44 years as the reference age group, and 1940–1944 as the reference birth cohort, and calculated the Risk Ratios (RR) of environmental-attributable IHD deaths and DALYs rates for each period and birth cohort. A two-sided *p* < 0.05 was considered statistically significant.

We chose 40–44 years as the reference age group because this middle adulthood period represents a point when IHD incidence begins to rise significantly, and individuals have accumulated sufficient environmental exposure time to serve as an ideal baseline for assessing the long-term impact of environmental factors. Additionally, this age group typically has relatively stable lifestyles and living environments, facilitating accurate assessment of environmental exposure effects while providing a sufficiently large sample size to ensure statistical reliability. We selected the period 2000–2004 as the reference period as it represents the approximate midpoint of our study timeframe (1990–2021), allowing for a clear interpretation of trends before and after this period.

The data used for these analyses are all publicly available from the GBD database and do not involve human subjects, therefore ethical review board approval was not required.

### 2.2 IHD and environmental risk factors definition

In the GBD 2021, IHD is defined according to the International Classification of Diseases (ICD) version 10, primarily coded as I20-I21.6, I21.9-I25.9, and Z82.4-Z82.49. MI is defined according to the Fourth Universal Definition of MI. Incidence is estimated for any MI, first-ever or recurrent. Stable angina is defined as reversible myocardial ischemia brought on by activity or stress, with diagnosis based on clinical symptoms (15).

Environmental risk factors examined in this research include particulate matter pollution, non-optimal temperature, and lead exposure. Particulate matter pollution includes two sources of PM2.5: ambient particulate matter pollution and household air pollution from the use of solid fuels for cooking, including coal, charcoal, wood, agricultural residue, and animal dung. The theoretical minimum risk exposure level (TMREL) for ambient particulate matter pollution is 2.4–5.9 μg/m^3^. Non-optimal temperature is an aggregate of the burden attributable to low and high temperatures. Heat and cold effects relate to effects above and below the TMREL. The population-weighted mean TMREL is 25.6°C. There are two types of lead exposure: acute exposure, measured by micrograms of lead per decilitre of blood, and chronic exposure, measured by micrograms of lead per gram of bone (Supplementary Table 5).

### 2.3 IHD burden estimation

According to the GBD 2021 methodology, IHD deaths were estimated using the Cause of Death Ensemble Modeling (CODEm) approach. Death models were established for both sexes and age groups ranging from 1 to over 95 years old. Disease Modeling Meta-Regression version 2.1 (DisMod-MR 2.1) was the main modeling tool for IHD, with maximum remission set as 1/13 (1 month). The GBD modeling incorporated several environmental and related covariates to improve estimation accuracy, including Outdoor pollution (PM2.5), Indoor air pollution, and Elevation over 1,500 m at Level 2, alongside other factors such as Healthcare access and quality index and Lag distributed income per capita that potentially interact with environmental exposures (Supplementary Table 6).

The GBD 2021 employed environmental covariates (including PM2.5 and indoor air pollution) in the CODEm models to estimate overall IHD mortality. Subsequently, the comparative risk assessment approach was used to calculate the proportion of disease burden attributable to each risk factor. Our study utilized these final attributable burden estimates, which represent the excess burden that would be eliminated if exposure to each environmental risk factor was reduced to the theoretical minimum risk exposure level.

DALYs rates were calculated as the sum of years of life lost (YLLs) due to premature deaths and years lived with disability (YLDs). YLLs were calculated by multiplying the number of deaths in each age group by the remaining life expectancy in that age group using the GBD standard life table. Disability weights for IHD were assigned according to different clinical manifestations and their severity levels. The GBD 2021 methodology classified acute myocardial infarction into two severity levels: days 1–2 with a disability weight of 0.432 (95% CI: 0.288–0.579) and days 3–28 with a disability weight of 0.074 (95% CI: 0.049–0.105). Angina was categorized as mild (DW: 0.033, 95% CI: 0.020–0.052), moderate (DW: 0.080, 95% CI: 0.052–0.113), or severe (DW: 0.167, 95% CI: 0.110–0.240) based on the Medical Expenditure Panel Survey (Supplementary Table 7). In the GBD study, based on the population size and IHD prevalence in different SDI regions, total and severity-specific numbers of IHD cases were calculated, and then severity-specific disability weights were assigned with comorbidity correction to calculate YLDs. Uncertainty was propagated by sampling 1,000 draws at each computational step, allowing for the combination of uncertainty from multiple sources.

### 2.4 Statistical analysis

To systematically analyze the influence of age, period, and cohort dimensions on the burden of IHD attributable to environmental factors, we applied the APC analysis model (17). Age effect reflects the pattern of IHD burden changes with increasing age; period effect represents the influence of specific historical periods, such as advances in diagnostic techniques, treatment methods, and environmental policies that affect all age groups; cohort effect reveals differences in environmental exposure and health risks faced by different birth cohorts. The APC model is expressed as follows:


Ln(Yij):μ+αi+βj+γk

Where *Y*ij represents the environmental factor-specific IHD deaths or DALYs rates, calculated as the number of deaths or DALYs attributed to environmental factors divided by total population, *α*i indicates the age effect in age group i, *β*j represents the period effect in period j, *γ*k denotes the RR of the birth cohort k related to age group i and period j (k: j-i), and *μ* is the intercept term (18).

Due to the “identification problem” arising from the linear dependency relationship in the APC model (Birth Cohort: Period-Age), we adopted the Intrinsic Estimator (IE) method to address the multicollinearity problem, which is based on the singular value decomposition of matrices. Net Drift reflects the overall annual percentage change in age-standardized rates, incorporating both period and cohort dimensions; Local Drift shows the annual percentage change for specific age groups, revealing differences in temporal trends across age groups.

### 2.5 Data processing and visualization tools

For the APC analysis, we utilized the online APC Web Tool provided by the National Institutes of Health (4), which facilitates the estimation of net drift, local drift, age-specific curves, and period and cohort relative risks. This tool allows for comprehensive decomposition of temporal trends into distinct age, period, and cohort effects.

All data processing, statistical analyses, and visualization were conducted using R version 4.4.3. We employed various R packages for data manipulation, statistical modeling, and creating visualizations to effectively represent the geographical and temporal patterns of environmental-attributable IHD burden across different SDI regions. Specifically, we employed the ‘tidyverse’ package suite for data manipulation, ‘ggplot2’ for creating figures, ‘sf’ and ‘rnaturalearth’ packages for geographical mapping, and base R functions for age-standardization calculations.

To ensure analytical robustness, we verified our extracted data against published GBD 2021 reports and conducted sensitivity analyses by examining trends across different SDI classification schemes. All statistical tests were two-sided with significance set at *p* < 0.05.

## 3 Results

### 3.1 Overall environmental burden of IHD

#### 3.1.1 Change of overall environmental-related IHD burden during 1990–2021

The global age-standardized deaths rates from environmentally related IHD decreased from 57.64 (95% CI: 45.79, 68.89) to 39.70 (95% CI: 30.74, 47.81) per 100,000 population between 1990 and 2021, representing a reduction of 31.13%. Similarly, the DALYs rates declined from 1,179.60 (95% CI: 942.76, 1,410.91) to 827.52 (95% CI: 648.13, 987.15) per 100,000 population, showing a decrease of 29.85%. However, these global improvements masked substantial disparities across development levels. A pronounced socioeconomic gradient emerged, with high SDI regions achieving remarkable reductions in deaths rates of 70.39%, while low SDI regions showed minimal progress with decreases of only 3.13%. This nearly 23-fold difference in improvement rates highlights significant global health inequities in environmental protection and healthcare access. Gender disparities were also evident throughout the study period. Males consistently demonstrated higher deaths rates and DALYs rates than females across all regions. Paradoxically, females exhibited greater relative improvements, with deaths rates reductions of 36.55% compared to 26.46% for males, suggesting differential benefits from environmental health interventions.

#### 3.1.2 Global burden of overall environmental-related IHD burden in 2021

**Figure 1:**
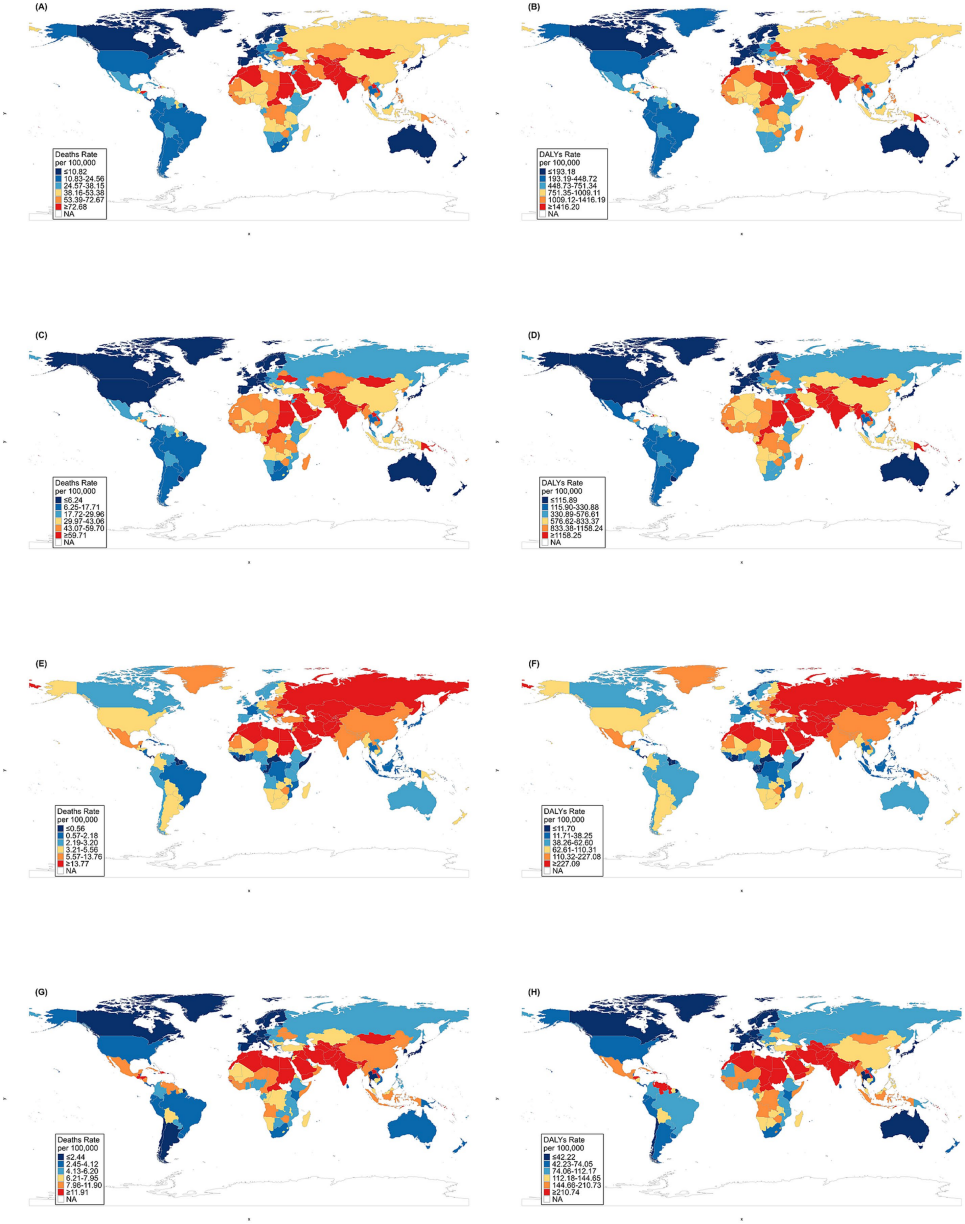
Age-standardized deaths and DALYs rates (per 100,000 population) of IHD attributed to overall environmental risk factors in 2021stratified by country. **(A)** Age-standardized IHD deaths rates attributed to overall environmental factors. **(B)** Age-standardized IHD DALYs rates attributed to overall environmental factors. **(C)** Age-standardized IHD deaths rates attributed to particulate matter pollution. **(D)** Age-standardized IHD DALYs rates attributed to particulate matter pollution. **(E)** Age-standardized IHD deaths rates attributed to non-optimal temperature. **(F)** Age-standardized IHD DALYs rates attributed to non-optimal temperature. **(G)** Age-standardized IHD deaths rates attributed to lead exposure. **(H)** Age-standardized IHD DALYs rates attributed to lead exposure.

The geographical analysis revealed distinct global distribution patterns of environmental-related IHD in 2021. High age-standardized deaths rates concentrated in North Africa, Middle East, and South Asia exceeded 72.68 per 100,000 population, with countries like Saudi Arabia, Iran, India, and Pakistan experiencing the highest values. These regions also showed elevated DALYs rates exceeding 1,416.20 per 100,000. Western Europe, North America, and parts of South America demonstrated lower deaths rates below 34.57 per 100,000. Australia showed unexpectedly high rates despite its high SDI status (Figures 1A,B). For gender analysis revealed that males in the Middle East, South Asia, and North Africa exhibited IHD deaths rates 1.5–2 times higher than females, while in North America and Australia, the gender gap was narrower with relatively higher female rates compared to global averages (Supplementary Figures 1A,B, 2A,B).

#### 3.1.3 APC analysis of overall environmental-related IHD burden

**Figure 2:**
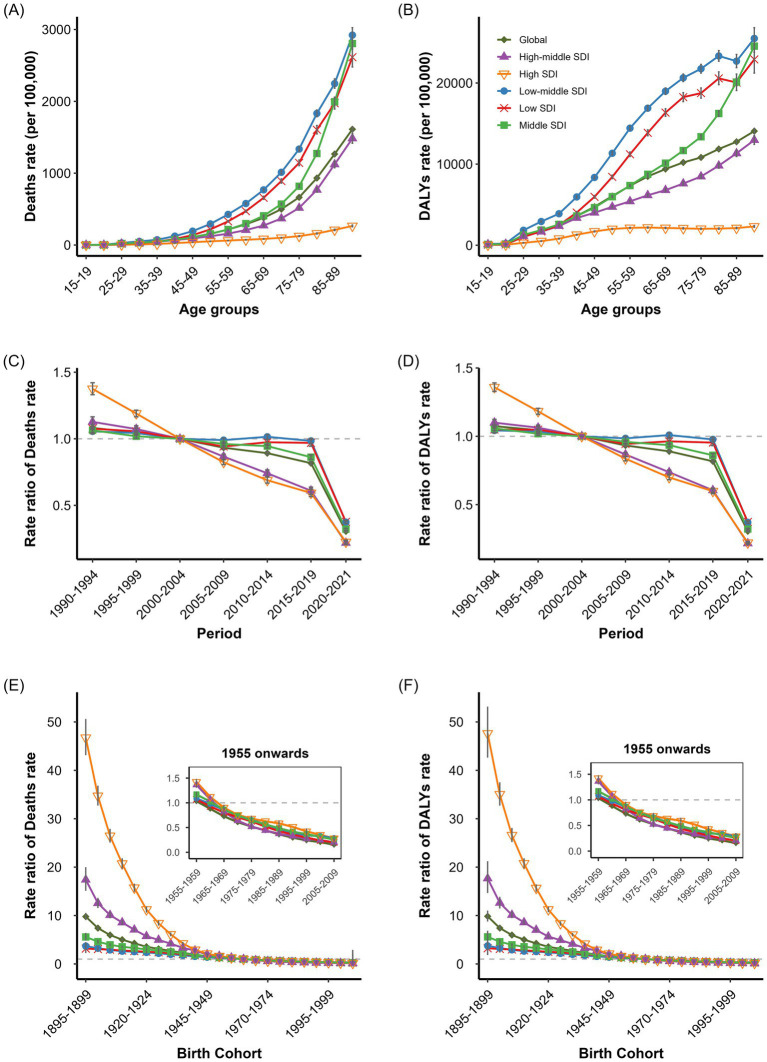
Age, period, and cohort effects on IHD deaths and DALYs rates attributed to overall environmental risk factors. **(A)** Age-specific IHD Deaths rates attributed to Overall Environmental by SDI regions. **(B)** Age-specific IHD DALYs rates attributed to Overall Environmental by SDI regions. **(C)** Period -specific IHD Deaths rates attributed to Overall Environmental by SDI regions. **(D)** Period -specific IHD DALYs rates attributed to Overall Environmental by SDI regions. **(E)** Cohort-specific IHD Deaths rates attributed to Overall Environmental by SDI regions. **(F)** Cohort-specific IHD DALYs rates attributed to Overall Environmental by SDI regions.

APC analysis revealed net drifts in environmental-related IHD deaths rates and DALYs rates of −3.098% (95% CI: −3.17, −3.026%) and −3.087% (95% CI: −3.133, −3.042%), respectively. High SDI regions demonstrated the most significant decline, with net drifts of −5.013% (95% CI: −5.287, −4.739%) and −4.991% (95% CI: −4.04, −3.82%), while low SDI regions showed the least improvement at −2.366% (95% CI: −2.498, −2.234%) and −2.347% (95% CI: −2.446, −2.249%; Supplementary Table 9). Against this backdrop, the environmental-related IHD burden increased markedly with age, demonstrating a clear age gradient. Low-middle SDI regions exhibited the highest burden across nearly all age groups, with deaths rates peaking at approximately 4,000 per 100,000 in the 65–75 age group, while high SDI regions maintained lower levels across all age brackets (Figures 2A,B). All regions displayed an age-ascending pattern that formed an inverse “V” shape in older adult populations. All SDI regions showed a downward trend in environmental-related IHD risk over time. Period rate ratios in high SDI regions declined from 1.40 in 1990–1994 to 0.36 in 2015–2019, while low SDI regions demonstrated limited improvement, decreasing from 1.12 to 0.64. Notably, low SDI regions exhibited distinct fluctuations during 2005–2015, contrasting with the steady decline observed in other regions (Figures 2C,D). Early birth cohorts (1900–1920) showed significantly higher RR than recent cohorts. High SDI regions demonstrated the most dramatic decline in cohort effects, with deaths RR for the 1900–1904 cohort reaching 20 before rapidly declining, while middle and low SDI regions showed relatively modest changes. The cohort effect curves across different SDI regions converged near the reference cohort (1940–1944), with low SDI regions gradually surpassing high SDI regions in the most recent cohorts (Figures 2E,F). For gender analysis revealed that males consistently exhibited higher environmental-related IHD burden than females; however, females showed greater improvement with net drifts of −3.334% (95% CI: −3.428, −3.24%) and −3.325% (95% CI: −3.383, −3.267%), compared to males at −2.933% (95% CI: −3.04, −2.825%) and −2.924% (95% CI: −2.994, −2.854%; Supplementary Table 9). Males demonstrated a more pronounced decline in cohort effects than females, particularly in high SDI regions (Supplementary Figures 3, 4).

**Figure 3:**
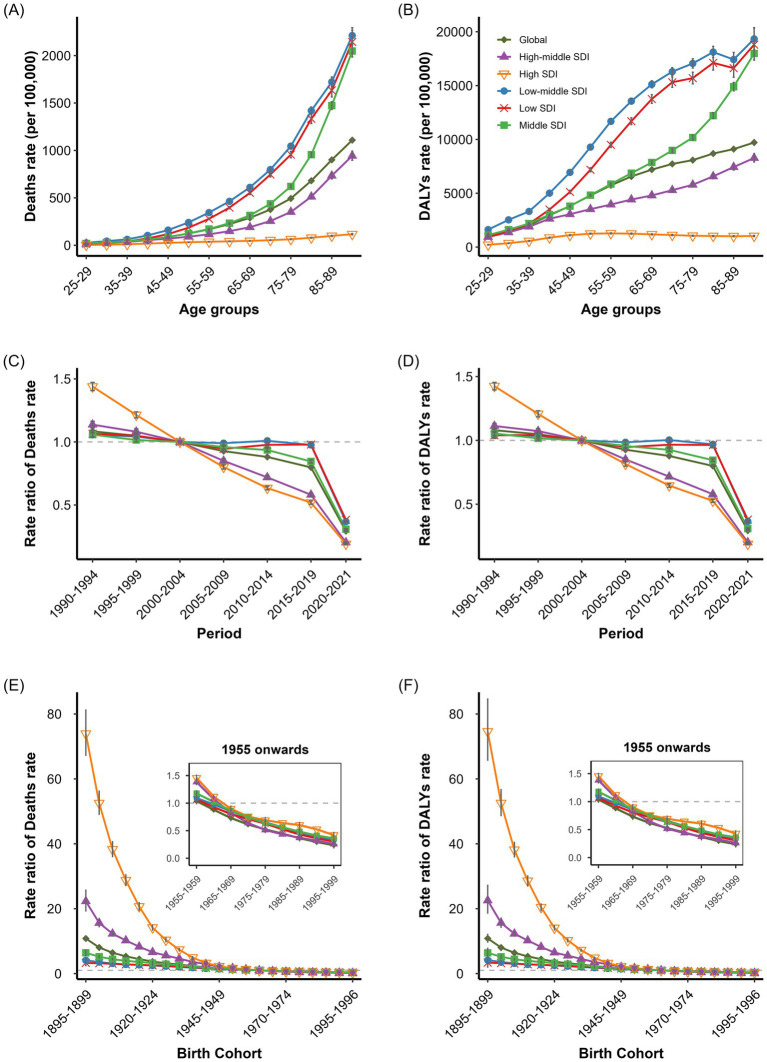
Age, period, and cohort effects on IHD deaths and DALYs rates attributed to non-optimal temperature. **(A)** Age-specific IHD deaths rates attributed to non-optimal temperature by SDI regions. **(B)** Age-specific IHD DALYs rates attributed to non-optimal temperature by SDI regions. **(C)** Period -specific IHD deaths rates attributed to non-optimal temperature by SDI regions. **(D)** Period -specific IHD DALYs rates attributed to non-optimal temperature by SDI regions. **(E)** Cohort-specific IHD deaths rates attributed to non-optimal temperature by SDI regions. **(F)** Cohort-specific IHD DALYs rates attributed to non-optimal temperature by SDI regions.

### 3.2 Specific environmental risk factors analysis

#### 3.2.1 Change of specific environmental risk factor-related IHD burden during 1990–2021

Between 1990 and 2021, IHD burden attributable to specific environmental factors exhibited notable patterns across various dimensions. As the predominant contributor, particulate matter pollution demonstrated substantial reductions, with deaths decreasing from 1,570,390 (95% CI: 1,199.84, 1,966.48) to 610,520 (95% CI: 1,866.68, 3,103.25; 56.74% reduction) and DALYs declining from 36,568,810 (95% CI: 28,680.23, 45,510.30) to 54,675,670 (95% CI: 41,652.49, 67,418.89; 49.21% reduction). Consequently, the age-standardized deaths rates decreased from 45.07 to 28.88 per 100,000 population (33.70% reduction), with DALYs rates following similar trends. Gender disparities persisted consistently across all environmental factors; notably, in 2021, male age-standardized deaths rates for particulate matter pollution reached 37.98 per 100,000 compared to 23.18 for females, with analogous patterns observed for non-optimal temperature and lead exposure, albeit these gender gaps narrowed throughout the study period. Furthermore, a pronounced socioeconomic gradient emerged in environmental risk improvement. Whilst high SDI regions achieved substantial progress with particulate matter pollution deaths rates declining by 75.65%, high-middle SDI regions by 48.96%, and middle SDI regions by 21.25%, Low-middle and low SDI regions exhibited considerably slower progress with reductions of merely 8.89 and 4.66%, respectively. Paradoxically, certain environmental factors worsened in resource-limited settings, as evidenced by non-optimal temperature-related IHD deaths rates increasing by 5.89% in low-middle SDI regions and by 0.94% in low SDI regions. Similarly, lead exposure-related deaths rates rose by 7.12% in low-middle SDI regions and 5.98% in low SDI regions. These adverse trends were likewise reflected in DALYs rates, with non-optimal temperature-related DALYs rates increasing by 4.82% in low-middle SDI regions and lead exposure-related metrics rising by 4.51% in middle SDI regions (Table 1; Supplementary Table 8).

**Table 1:** Change in environmental-related IHD deaths rates and DALYs rates (per 100,000 population) from 1990 to 2021.

Risk factor	Measure metric	Categories	Global	Sex		SDI^a^ region				
				Male	Female	High SDI	High-middle SDI	Middle SDI	Low-middle SDI	Low SDI
Environmental	Deaths Rates	1990	57.64 (45.79, 68.89)	69.27 (54.76, 83.24)	48.04 (37.87, 57.92)	40.73 (29.82, 51.97)	68.51 (52.16, 84.43)	57.17 (45.91, 67.78)	71.42 (58.02, 84.41)	63.93 (51.08, 76.53)
		2021	39.70 (30.74, 47.81)	50.94 (39.30, 62.02)	30.48 (23.53, 36.83)	12.06 (8.84, 15.01)	39.08 (29.07, 48.03)	47.97 (36.18, 58.25)	67.64 (53.54, 80.26)	61.93 (49.91, 73.60)
		%Ch ^b^	−31.13%	−26.46%	−36.55%	−70.39%	−42.96%	−16.09%	−5.29%	−3.13%
	DALYs Rates	1990	1179.60 (942.76, 1410.91)	1479.71 (1174.30, 1766.57)	908.30 (727.23, 1092.99)	761.97 (565.29, 973.24)	1280.03 (990.29, 1578.32)	1167.15 (945.02, 1387.83)	1630.10 (1320.96, 1926.39)	1418.49 (1124.88, 1702.88)
		2021	827.52 (648.13, 987.15)	1, 089.67 (856.84, 1318.28)	591.33 (460.69, 707.35)	240.11 (182.12, 296.64)	696.38 (537.62, 865.95)	927.67 (717.42, 1126.57)	1, 630.10 (1, 320.96, 1, 926.39)	1293.39 (1037.81, 1535.99)
		%Ch ^b^	−29.85%	−26.36%	−34.90%	−68.48%	−45.6%	−20.52%	−9.08%	−8.82%
Particulate matter pollution	Deaths Rates	1990	45.07 (33.95, 56.58)	53.53 (40.78, 66.90)	37.99 (28.78, 48.21)	27.68 (17.08, 38.64)	54.37 (38.86, 71.73)	47.16 (37.00, 57.56)	59.50 (46.69, 72.76)	54.56 (43.15, 67.43)
		2021	29.88 (22.33, 37.22)	37.98 (27.93, 47.84)	23.18 (17.16, 28.93)	6.74 (4.57, 9.04)	27.75 (19.77, 35.86)	37.14 (27.41, 46.51)	54.21 (41.50, 66.54)	52.02 (40.80, 63.49)
		%Ch ^b^	−33.70%	−29.05%	−39.00%	−75.65%	−48.96%	−21.25%	−8.89%	−4.66%
	DALYs Rates	1990	936.39 (730.00, 1165.83)	1160.90 (902.95, 1443.45)	731.75 (560.84, 913.33)	522.60 (327.98, 722.10)	1018.96 (741.70, 1339.39)	965.48 (760.63, 1181.74)	1361.99 (1070.32, 1663.22)	1210.91 (955.10, 1497.44)
		2021	638.48 (486.47, 787.82)	833.35 (623.99, 1048.38)	461.65 (348.37, 572.47)	140.69 (97.82, 186.40)	501.39 (357.16, 654.56)	728.13 (546.26, 913.14)	1198.30 (934.51, 1463.54)	1094.12 (864.87, 1342.30)
		%Ch ^b^	−31.82%	−28.22%	−36.91%	−73.08%	−50.80%	−24.58%	−12.02%	−9.64%
Non-optimal temperature	Deaths Rates	1990	936.39 (730.00, 1165.83)	1160.90 (902.95, 1443.45)	731.75 (560.84, 913.33)	10.75 (9.12, 14.12)	13.43 (11.58, 17.12)	8.06 (5.83, 10.80)	9.00 (5.48, 12.90)	6.35 (4.02, 9.13)
		2021	638.48 (486.47, 787.82)	833.35 (623.99, 1048.38)	461.65 (348.37, 572.47)	4.25 (3.43, 5.82)	9.07 (7.30, 12.61)	7.85 (5.93, 10.74)	9.53 (5.74, 14.23)	6.41 (4.17, 9.00)
		%Ch ^b^	−31.82%	−28.22%	−36.91%	−60.47%	−32.44%	−2.61%	5.89%	0.94%
	DALYs Rates	1990	10.57 (8.63, 13.74)	12.49 (10.22, 16.41)	8.95 (7.26, 11.57)	196.60 (170.05, 257.76)	244.78 (210.67, 311.09)	161.04 (117.42, 217.83)	202.03 (121.50, 289.48)	142.43 (90.24, 206.68)
		2021	7.39 (5.57, 10.44)	9.33 (7.05, 12.98)	5.79 (4.35, 8.14)	83.20 (66.68, 116.00)	158.14 (127.62, 218.78)	147.98 (110.75, 202.53)	211.78 (125.60, 317.34)	133.91 (86.51, 190.05)
		%Ch ^b^	−30.09%	−25.30%	−35.31%	−57.68%	−35.39%	−8.11%	4.82%	−5.98%
Lead exposure	Deaths Rates	1990	7.91 (−1.15, 16.94)	10.80 (−1.58, 22.98)	5.70 (−0.83, 12.15)	5.46 (−0.77, 11.94)	6.87 (−0.97, 14.86)	8.42 (−1.21, 17.89)	12.64 (−1.93, 26.76)	11.54 (−1.79, 23.92)
		2021	7.11 (−1.01, 14.88)	9.93 (−1.42, 20.76)	4.91 (−0.69, 10.50)	2.02 (−0.28, 4.39)	6.15 (−0.85, 13.10)	8.80 (−1.25, 18.38)	13.54 (−1.96, 28.81)	12.23 (−1.80, 25.27)
		%Ch ^b^	−10.11%	−8.06%	−13.86%	−63.00%	−10.48%	4.51%	7.12%	5.98%
	DALYs Rates	1990	166.11 (−24.29, 359.91)	230.62 (−33.86, 498.48)	110.66 (−16.08, 234.59)	102.41 (−14.39, 221.26)	132.97 (−18.71, 289.85)	172.53 (−24.69, 372.09)	285.91 (−43.55, 614.67)	253.44 (−38.60, 532.62)
		2021	138.57 (−19.52, 289.73)	195.17 (−27.66, 407.75)	89.67 (−12.52, 189.54)	36.18 (−5.10, 77.11)	103.45 (−14.06, 220.96)	157.02 (−22.14, 328.22)	275.71 (−39.54, 589.56)	239.69 (−35.02, 502.18)
		%Ch ^b^	−16.58%	−15.37%	−18.97%	−64.67%	−22.20%	−8.99%	−3.57%	−5.42%

All rates are presented as age-standardized rates per 100,000 population with 95% confidence intervals in parentheses. Statistical significance was determined using two-sided t-tests, with *p* < 0.05 considered significant.

a SDI, Socio-demographic Index, categorized as High (>0.81), High-middle (0.70–0.81), Middle (0.61–0.69), Low-middle (0.46–0.60), and Low (<0.46).

b %Ch = Percent change, represents the relative change in rates from 1990 to 2021. Negative values indicate a decrease in rates; positive values indicate an increase.

#### 3.2.2 Global burden of specific environmental risk factor-related IHD burden in 2021

The geographical distribution of particulate matter pollution burden revealed distinct regional patterns. The highest burden concentrated predominantly in South Asia, Middle East, and North Africa with deaths rates ≥ 43.07 per 100,000 and DALYs rates ≥ 576.82 per 100,000; meanwhile, Sub-Saharan Africa and East Asia exhibited middle-level burden with deaths rates 17.72–43.06 per 100,000 and DALYs rates 330.88–576.81 per 100,000; conversely, the Americas, Europe, and Oceania demonstrated the lowest burden with deaths rates ≤ 17.71 per 100,000 and DALYs rates ≤ 330.87 per 100,000. Notably, within-region variations were particularly pronounced in Africa (Figures 1C,D). Gender analysis highlighted that males generally experienced higher burden levels globally, although Western Europe and parts of North America displayed narrower gender gaps, whereas South Asia, the Middle East, and North Africa exhibited the most substantial male–female disparities (Supplementary Figures 1C,D, 2C,D).

Non-optimal temperature burden displayed a distinctive geographical distribution. The highest burden occurred primarily in the Middle East, North Africa, and Central Asia with deaths rates ≥ 5.57 per 100,000 and DALYs rates ≥ 110.32 per 100,000; in contrast, Russia, the southern United States, and parts of Africa showed middle-level burden with deaths rates 2.19–5.56 per 100,000 and DALYs rates 38.25–110.31 per 100,000; whilst most of South America, Australia, and Canada presented the lowest burden with deaths rates ≤ 2.18 per 100,000 and DALYs rates ≤ 38.24 per 100,000. Significant north–south variations were evident within Africa, alongside distinct differences between Central and East Asia (Figures 1E,F). Gender analysis revealed that the male–female gap appeared wider in tropical and subtropical regions than temperate zones, with marked regional characteristics in regions experiencing extreme seasonal temperature fluctuations, such as Central Asia and Eastern Europe (Supplementary Figures 1E,F, 2E,F).

Lead exposure demonstrated a remarkably distinctive distribution pattern compared to other environmental factors. The highest burden was predominantly found in North America, Canada, and Australia with deaths rates ≥ 7.96 per 100,000 and DALYs rates ≥ 144.66 per 100,000; by comparison, parts of Europe and Africa exhibited middle-level burden with deaths rates 4.15–7.95 per 100,000 and DALYs rates 74.05–144.65 per 100,000; whilst South America and Russia showed the lowest burden with deaths rates ≤ 4.14 per 100,000 and DALYs rates ≤ 74.04 per 100,000. Within-region variations were notable, particularly east–west differences in Europe and complex inter-country variations in Africa (Figures 1G,H). Gender analysis revealed the most variable geographical patterns among environmental factors, with males showing substantially higher burden rates in regions with high industrial activity; however, the gender gap was less pronounced in regions where environmental contamination was the predominant exposure pathway, such as parts of South America and Southeast Asia (Supplementary Figures 1G,H, 2G,H).

### 3.3 APC analysis of specific environmental risk factor-related IHD burden

#### 3.3.1 APC Analysis of particulate matter pollution-related IHD burden

**Figure 4:**
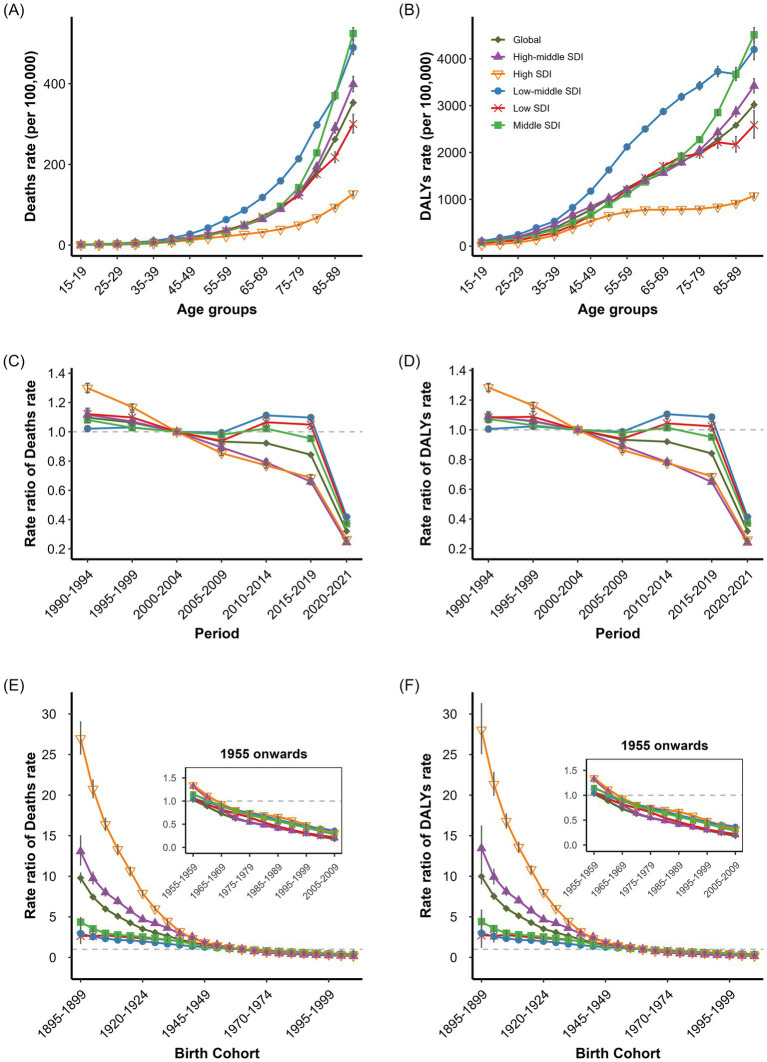
Age, period, and cohort effects on IHD deaths and DALYs rates attributed to particulate matter pollution. **(A)** Age-specific IHD deaths rates attributed to particulate matter pollution by SDI regions. **(B)** Age-specific IHD DALYs rates attributed to particulate matter pollution by SDI regions. **(C)** Period -specific IHD Deaths rates attributed to particulate matter pollution by SDI regions. **(D)** Period -specific IHD DALYs rates attributed to particulate matter pollution by SDI regions. **(E)** Cohort-specific IHD deaths rates attributed to particulate matter pollution by SDI regions. **(F)** Cohort-specific IHD DALYs rates attributed to particulate matter pollution by SDI regions.

APC analysis of particulate matter pollution-related IHD deaths and DALYs rates revealed distinct age, period, and cohort effects across global and SDI regions. The global net drifts in particulate matter pollution-attributable IHD deaths and DALYs rates were −3.23% (95% CI: −3.27, −3.19%) and −3.22% (95% CI: −3.25, −3.19%) respectively, with negative local drifts across all age groups (Supplementary Table 10). For age-specific analysis demonstrated particulate matter pollution-related IHD burden increased markedly with age across all SDI regions (Figures 3A,B). Low-middle SDI region exhibited the highest age-specific burden, with deaths rates rising from 1.0 per 100,000 in the 30–34 age group to over 21.0 per 100,000 in the 80–84 age group. Low SDI regions ranked second, whilst high SDI regions maintained the lowest rates throughout. Middle SDI region showed higher deaths rates than global average in populations over 70 years. Males demonstrated significantly higher particulate matter pollution-related IHD burden than females across all regions (Supplementary Figures 5A,B6A,B). For DALYs rates followed inverse “V” shaped trends in low-middle, low, and middle SDI regions, peaking at 354.62, 292.60, and 151.93 per 100,000 in the 70–74, 65–69, and 70–74 age groups, respectively. High and high-middle SDI regions exhibited monotonic decline after age 35–39 (Figures 4A,B). The period effect analysis showed a substantial global reduction in particulate matter pollution-related IHD burden from 1990 to 2019. Period rate ratios for deaths rates declined from 1.32 (95% CI: 1.30, 1.34) to 0.56 (95% CI: 0.55, 0.57), and for DALYs rates from 1.34 (95% CI: 1.32, 1.36) to 0.59 (95% CI: 0.58, 0.60). High SDI regions showed the most marked improvement, with deaths rates ratios decreasing from 5.71 (95% CI: 5.59, 5.82) to 0.53 (95% CI: 0.52, 0.54). Low SDI regions demonstrated the least improvement, with deaths rates ratios declining only from 1.18 (95% CI: 1.14, 1.22) to 0.63 (95% CI: 0.61, 0.65; Figures 4C,D). Cohort effect analysis revealed significant declines in particulate matter pollution-related IHD burden across successive birth cohorts in all SDI regions (Figures 4E,F). High SDI regions exhibited highest RR in early birth cohorts but showed rapid declines after the 1945–1949 cohort. Low SDI regions had relatively higher risks after the 1950–1954 cohort. Males showed more pronounced risk declines from early cohorts than females, particularly in high SDI regions (Supplementary Figures 5E,F, 6E,F).

#### 3.3.2 APC analysis of non-optimal temperature-related IHD burden

APC analysis of non-optimal temperature-related IHD deaths and DALYs rates demonstrated significant age, period, and cohort effects globally and across SDI regions. The global net drifts in temperature-attributable IHD deaths and DALYs rates were both −2.98% (95% CI: −3.03, −2.92% for deaths; −3.01, −2.94% for DALYs rates), with negative local drifts across all age groups (Supplementary Table 11). Age-specific analysis revealed non-optimal temperature-related IHD burden increased substantially with age across all SDI regions (Figures 3A,B). Low SDI region exhibited the highest burden, with deaths rates rising from 0.32 per 100,000 in the 15–19 age group to 7.53 per 100,000 in the 60–64 age group before declining slightly in older ages. Low-middle SDI region ranked second, whilst high and high-middle SDI regions maintained lower rates throughout. Middle SDI region temperature-related IHD deaths rates were highest in the 50–79 age range at approximately 1.25 per 100,000. Male adults from low and low-middle SDI regions demonstrated markedly higher deaths rates than females, despite similar age-specific patterns (Supplementary Figures 7A,B, 8A,B). For DALYs rates, followed inverse “V” shaped trends in low, low-middle, and middle SDI regions, with peak values in the 60–64 age group for low and low-middle regions and in the 55–59 age group for middle SDI regions (Figures 3A,B). Males consistently exhibited higher burden across all age groups (Supplementary Figures 7A,B, 8A,B). The period effect analysis demonstrated consistent declines in temperature-related IHD burden from 1990 to 2019. Global deaths rate ratios declined from 1.32 (95% CI: 1.30, 1.34) to 0.56 (95% CI: 0.55, 0.57; Figures 3C,D). High SDI regions showed the most substantial improvement, decreasing deaths rate ratios from 4.26 (95% CI: 4.08, 4.44) to 0.43 (95% CI: 0.41, 0.44). Low SDI regions showed modest improvement with ratios declining only from 1.18 (95% CI: 1.14, 1.22) to 0.63 (95% CI: 0.61, 0.65). Both sexes exhibited similar period effect patterns (Supplementary Figures 7C,D, 8C,D). The cohort effect analysis revealed monotonic decreases in temperature-related IHD burden across successive birth cohorts in all SDI regions (Figures 3E,F). High SDI regions demonstrated the most pronounced effects, with deaths rate ratios declining from 5.27 (95% CI: 4.44, 6.26) in the 1910–1914 birth cohort to 0.47 (95% CI: 0.43, 0.51) in recent cohorts. Low SDI regions showed less substantial improvements. Males demonstrated greater improvement magnitude in recent cohorts than females (Supplementary Figures 7E,F, 8E,F).

#### 3.3.3 APC analysis of lead exposure -related IHD burden

**Figure 5:**
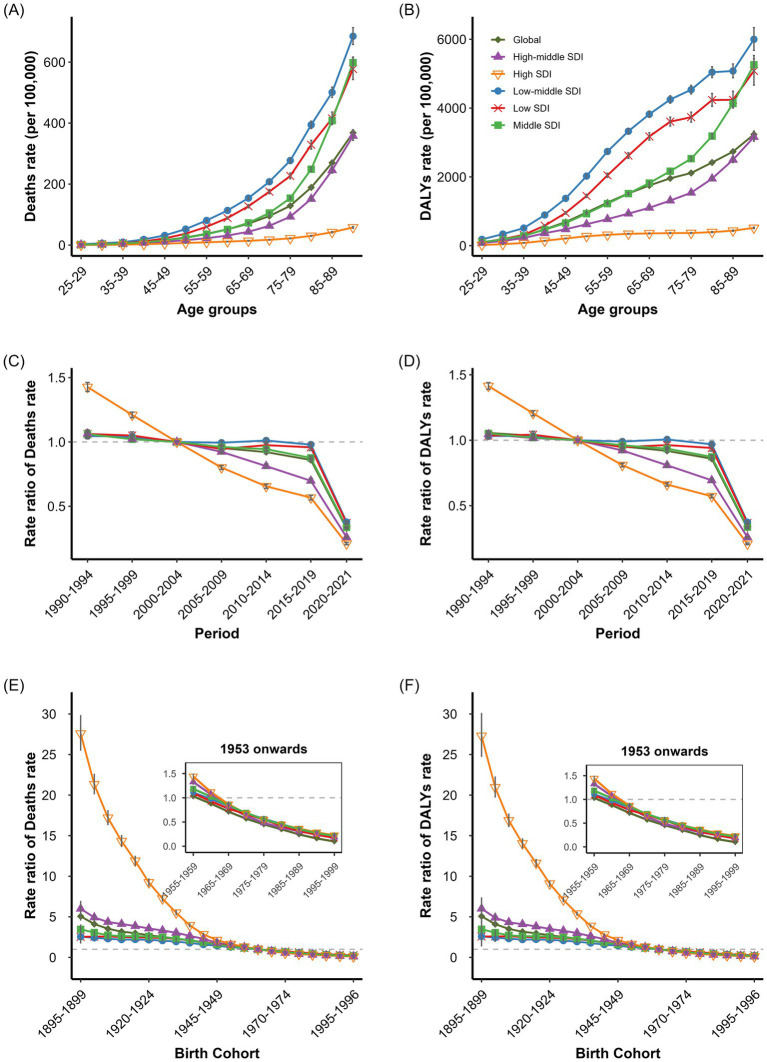
Age, period, and cohort effects on IHD deaths and DALYs rates attributed to lead exposure. **(A)** Age-specific IHD Deaths rates attributed to Lead exposure by SDI regions. **(B)** Age-specific IHD DALYs rates attributed to lead exposure by SDI regions. **(C)** Period -specific IHD Deaths rates attributed to lead exposure by SDI regions. **(D)** Period -specific IHD DALYs rates attributed to lead exposure by SDI regions. **(E)** Cohort-specific IHD Deaths rates attributed to lead exposure by SDI regions. **(F)** Cohort-specific IHD DALYs rates attributed to lead exposure by SDI regions.

APC analysis of lead exposure-related IHD deaths and DALYs rates revealed notable age, period, and cohort effects globally and across SDI regions. The global net drifts in lead exposure-attributable IHD deaths and DALYs rates were −2.80% (95% CI: −2.86, −2.74%) and −2.79% (95% CI: −2.83, −2.74%), respectively, with negative local drifts across all age groups (Supplementary Table 12). For age-specific analysis demonstrated lead exposure-related IHD burden increased substantially with age across all SDI regions (Figures 5A,B). Markedly high burden was observed in adults aged 25–29 years with deaths rates of 4.37 per 100,000, reflecting early life lead exposure effects. The highest overall burden occurred in low-middle SDI region, followed by low SDI region, with deaths rates peaking at approximately 12 per 100,000 in the 80–84 age group. High and high-middle SDI regions maintained lower rates throughout. Lead exposure-related IHD deaths rates were consistently higher in males than females across all age groups (Supplementary Figures 9A,B, 10A,B). For DALYs rates followed inverse “V” shaped trends in low-middle, low, and middle SDI regions, peaking in the 60–64 age group for low-middle SDI regions and in the 55–59 age group for low SDI regions (Figures 5A,B). High SDI regions exhibited relatively elevated DALYs rates in younger age groups compared to other environmental exposures. Males consistently demonstrated higher burden than females across all age groups (Supplementary Figures 9A,B, 10A,B). For period effect analysis showed declines in lead exposure-related IHD burden from 1990 to 2019, though less pronounced than for other environmental factors. Global period rate ratios for deaths rates decreased from 1.20 (95% CI: 1.16, 1.24) to 0.64 (95% CI: 0.61, 0.66; Figures 5C,D). High SDI regions demonstrated most substantial improvement, with deaths rate ratios declining from 1.48 (95% CI: 1.37, 1.59) to 0.47 (95% CI: 0.43, 0.51). Low and low-middle SDI regions experienced less significant improvements, suggesting persistent lead exposure challenges (Supplementary Figures 9C,D, 10C,D). For cohort effect analysis revealed decreasing trends in lead exposure-related IHD burden across successive birth cohorts in all SDI regions (Figures 5E,F). High SDI regions demonstrated most marked effects, with deaths rate ratios declining from 16.35 (95% CI: 12.51, 21.38) in the 1900–1904 birth cohort to approximately 1.0 in recent cohorts. Males exhibited greater improvement magnitude in recent cohorts than females, particularly in high SDI regions (Supplementary Figures 9E,F, 10E,F).

## 4 Discussion

### 4.1 Plenary findings

This study systematically evaluates the global burden of IHD attributable to environmental factors (particulate matter pollution, non-optimal temperature, and lead exposure), analyzing temporal trends during 1990–2021, geographical distribution patterns, and APC effects across different SDI regions. The analysis found that environment-related IHD burden decreased significantly but unevenly worldwide. Particulate matter pollution emerged as the predominant environmental contributor to IHD, while non-optimal temperature and lead exposure showed varying improvement patterns. Geographical analysis revealed highest environmental IHD burden in South Asia, the Middle East, and North Africa, reflecting inadequate environmental regulations and healthcare infrastructure in these regions. APC analysis demonstrated that environmental IHD burden increases exponentially with age. High SDI regions achieved substantial improvements through effective environmental policies, while low SDI regions showed limited progress, with lead exposure and non-optimal temperature-related IHD burden even increasing in some regions. Gender analysis indicated consistently higher environmental IHD burden in males than females, reflecting differences in occupational and behavioral exposure patterns.

### 4.2 Environmental factors and IHD relationship

The analysis found that environment-related IHD deaths rates decreased globally by 31.13% between 1990 and 2021, with DALYs rates declining by 29.85%. Understanding the mechanisms behind these patterns is crucial for interpreting our findings. These environmental factors influence cardiovascular health through multiple pathophysiological mechanisms, including systemic inflammation (19–21), oxidative stress (19, 20, 22), endothelial dysfunction (19–22), and autonomic nervous system dysregulation (19–21, 23), ultimately promoting atherosclerosis, thrombosis, and increased susceptibility to ischemic events (19–21). However, these improvements were not uniformly distributed globally. Our research reveals uneven geographical distribution of environment-related IHD burden, with South Asia (India, Pakistan), Middle East (Saudi Arabia, Iran), and North Africa (Egypt) showing highest burden levels. This geographical clustering reflects the combined effects of increased environmental exposures, inadequate regulatory frameworks, and limited healthcare infrastructure in these regions (24, 25). Beyond geographical patterns, our APC analysis reveals important temporal and demographic dimensions. Environment-related IHD burden increases exponentially with age across all regions, with high SDI regions achieving substantial improvements while low SDI regions showing limited progress, and earlier birth cohorts experiencing higher risk.

Our APC analysis shows that environment-related IHD burden rises exponentially with age across all regions, a pattern that results from multiple underlying mechanisms (25). Primarily, cumulative lifetime exposure to environmental pollutants results in progressive vascular damage and atherosclerotic progression (19–21). Furthermore, age-related physiological changes, including decreased cardiovascular reserve and impaired endothelial function, enhance vulnerability to environmental stressors (25, 26). Lastly, older adult populations exhibit reduced thermoregulatory capacity and decreased ability to metabolize toxins, amplifying the cardiovascular impacts of temperature extremes and lead exposure (26–28). These age-related vulnerabilities underscore the importance of protecting older adult populations in environmental health policies. Period effect analysis reveals significant socioeconomic gradient, with high SDI regions achieving substantial improvement during the study period (period risk ratio decreasing from 1.40 to 0.36), while low SDI regions showed limited progress (from 1.12 to 0.64) (25), This disparity may be attributed to earlier implementation and more effective enforcement of environmental protection policies in high-income regions, resulting in long-term reductions in environmental exposure (29). Period effect analysis reveals significant socioeconomic gradient, with high SDI regions achieving substantial improvement during the study period (period risk ratio decreasing from 1.40 to 0.36), while low SDI regions showed limited progress (from 1.12 to 0.64). This disparity may be attributed to earlier implementation and more effective enforcement of environmental protection policies in high-income regions, resulting in long-term reductions in environmental exposure. Successful policy interventions demonstrate the potential for reducing environmental-related ischemic heart disease burden through comprehensive regulatory frameworks. China’s Air Pollution Prevention and Control Action Plan (2013–2017) reduced PM2.5-related ischemic heart disease deaths from 106,010 cases in 2013 to 97,790 cases in 2017, while PM2.5 concentrations decreased by 33.3% (30). France’s National Heat Wave Plan implemented after the 2003 heat wave prevented approximately 4,400 deaths during the 2006 heat wave compared to expected mortality (31). The United States’ lead paint ban in the 1970s resulted in a 90% decline in blood lead levels, reducing cardiovascular disease risk, while 21 countries have enacted similar legislation with UNEP-WHO support (32). Economic analysis demonstrates that a $10 per capita public health investment saves 9.1 lives per 100,000, with cardiovascular disease prevention as a major beneficiary, yielding $1.50 in social benefits for every $1 invested (33). This evidence underscores the effectiveness of comprehensive regulatory frameworks in reducing environmental-related ischemic heart disease burden, with actual policy results directly demonstrating that environmental interventions can significantly reduce ischemic heart disease burden. Cohort effect analysis indicates significantly higher RR in earlier birth cohorts (1900–1920) compared to recent cohorts, with high SDI regions showing most pronounced decline in cohort effects, reflecting cumulative benefits of environmental protection measures across generations. In response to these age-related vulnerability patterns, age-related environmental IHD patterns demand age-friendly environmental policies protecting both older adult populations and younger generations.

Finally, building upon these temporal patterns revealed by APC analysis, our investigation identified significant gender disparities in environmental-related IHD burden. Males consistently demonstrate higher burden than females across all environmental exposures and SDI regions, with the gap most pronounced in South Asia and the Middle East, consistent with previous research (26, 34). This differential burden reflects the complex interplay of occupational, behavioral, and biological factors. First of all, regarding occupational exposures, with males disproportionately represented in high-exposure industries including construction, mining, and manufacturing (35, 36), where PM2.5 levels can exceed ambient concentrations by several fold and temperature/lead exposures occur through industrial processes (37). Secondly, concerning behavioral factors, particularly smoking with global rates of approximately 35% in males versus 6%in females, may amplify cardiovascular effects through synergistic mechanisms with environmental exposures (38, 39). Furthermore, emerging evidence suggests sex-specific biological responses to environmental stressors. Males potentially exhibit greater vascular inflammation and endothelial dysfunction following PM2.5 exposure (40, 41), coupled with differential thermoregulatory responses to temperature extremes (27, 28), and enhanced susceptibility to lead-induced cardiovascular toxicity (9, 23). These findings underscore the need for gender-sensitive environmental health interventions, particularly enhanced occupational safety standards in male-dominated industries combined with targeted health promotion strategies. Consequently, concerning gender disparities across environmental exposures warrant specific policy responses. Concerning gender disparities in environmental IHD burden, environmental health policies should account for gender-specific exposure patterns and vulnerabilities, with particular attention to occupational protections in male-dominated high-risk industries.

These observed geographical and demographic disparities highlight the need for targeted interventions. Concerning regional inequalities in environmental burden, governments in low and low-middle SDI regions (Sub-Saharan Africa, South Asia) should integrate environmental health into national development strategies while enhancing regulatory frameworks and research capabilities. For healthcare professionals in these regions, implementation of comprehensive cardiovascular screening protocols that incorporate environmental exposures is essential, whereas community engagement in environmental health initiatives remains fundamental to sustainable progress.

### 4.3 Particulate matter pollution-related IHD burden

Particulate matter pollution represents the predominant environmental contributor to IHD burden. The study found global particulate matter-attributable IHD deaths rates decreased by 33.70% between 1990 and 2021, with DALYs rates declining by 31.82%. Particulate matter exposure induces cardiovascular pathology through multiple pathways, including systemic inflammation, oxidative stress, endothelial dysfunction, and autonomic dysregulation (19–21). These changes accelerate atherosclerotic progression, enhance thrombogenicity, and increase myocardial susceptibility to ischemic injury (19, 20). Our geographical analysis revealed pronounced particulate matter-related IHD burden in South Asia, the Middle East, and North Africa. APC analysis demonstrated that particulate matter-related IHD burden increased markedly with age, with high SDI regions achieving the most substantial period and cohort improvements. Gender analysis showed consistently higher particulate matter-related IHD burden in males. Our geographical analysis revealed pronounced particulate matter-related IHD burden in South Asia (India, Pakistan), the Middle East (Saudi Arabia, Iran), and North Africa (Egypt), regions characterized by rapid industrialization without commensurate environmental safeguards (14, 42). Our APC analysis demonstrated that particulate matter-related IHD burden increased exponentially with age, peaking in older adult populations, consistent with cumulative vascular damage from prolonged exposure (14, 42) and age-associated cardiovascular vulnerability (19). Period effect analysis revealed marked regional disparities in particulate matter-related IHD burden reduction. High SDI regions (such as Europe and North America) achieved substantial improvements during the study period through stringent emissions standards and comprehensive air pollution control measures (14, 30). In contrast, middle and low-middle SDI regions faced the dual challenge of expanding industrial activities with limited air quality management infrastructure (14, 42), showing minimal progress. Cohort effect analysis showed significant risk reductions across successive cohorts in high SDI regions, while low SDI regions demonstrated relatively stable cohort patterns, indicating limited intergenerational improvements in environmental protection (14, 42, 43). Finally, our gender analysis revealed consistently higher particulate matter-attributable IHD burden in males, reflecting a combination of exposure and physiological factors (40, 41). In occupational exposure, males demonstrate higher participation rates in high-exposure industries such as mining and construction (35, 36). In behavioral exposure, global male smoking rates significantly exceed female rates (39). Although research indicates females may have higher biological susceptibility to particulate matter exposure, the greater cumulative exposure patterns among males drive their higher overall disease burden (40). To address these gender-specific exposure patterns and vulnerabilities, healthcare providers should develop gender-differentiated cardiovascular health promotion strategies, complemented by targeted educational initiatives addressing occupation-specific risks among males.

These findings underscore the critical need for targeted interventions. For particulate matter pollution as the predominant environmental contributor, policy interventions should focus on establishing robust air quality monitoring networks in high-burden regions including South Asia (India, Pakistan), Middle East (Saudi Arabia, Iran), and North Africa (Egypt), coupled with strengthened emission standards and urban planning policies. Notably, healthcare systems should incorporate air quality risk assessment into routine cardiovascular examinations, while vulnerable populations, particularly those with pre-existing cardiovascular conditions, require accurate information and appropriate protective guidance.

### 4.4 Non-optimal temperature-related IHD burden

Non-optimal temperature exposure constitutes a significant environmental contributor to IHD burden. The analysis revealed that global age-standardized non-optimal temperature-related IHD deaths rates decreased by 30.09% between 1990 and 2021, with DALYs rates declining by 27.69%. Non-optimal temperature affects cardiovascular function through several physiological stress mechanisms, including altered blood viscosity (through dehydration and blood concentration), autonomic nervous system perturbations (manifested as sympathetic activation and vasoconstriction), and systemic inflammatory responses (8, 19, 21). Our geographical analysis demonstrated that temperature-attributable IHD burden was primarily concentrated in the Middle East, North Africa, and parts of Central Asia. APC analysis indicated that temperature-related IHD burden increased with age, peaking in older adult populations, with low SDI regions exhibiting the highest burden. Period effect analysis showed significant improvements in high SDI regions, while low-middle SDI regions displayed concerning increasing trends. Cohort analysis revealed higher RR in earlier birth cohorts, with high SDI regions demonstrating the most pronounced decline in cohort effects. Gender analysis showed consistently higher temperature-related IHD burden in males, though this disparity varied by region. Our analysis revealed that temperature-attributable IHD burden exhibited distinctive regional patterns, with highest rates concentrated in the Middle East (Saudi Arabia, UAE), North Africa (Egypt, Algeria), and parts of Central Asia (Kazakhstan, Uzbekistan) (26, 44). This distribution is influenced by regional climate characteristics and varying levels of adaptive capacity in these populations (26, 44, 45). Our APC analysis demonstrated significant temperature-related IHD burden in older populations across all SDI regions, with low SDI regions exhibiting the highest rates (26). This age-dependent vulnerability reflects diminished thermoregulatory capacity and cardiovascular reserve in older adult individuals (26). Addressing this age-dependent vulnerability requires that in the clinical context, cardiovascular screening protocols should integrate environmental risk assessments for older adults, while community-level interventions should emphasize older adult environmental vulnerability, particularly during extreme weather events. Contrary to the overall global improvement pattern, low-middle SDI regions showed concerning trends, with temperature-related IHD deaths rates increasing by 5.89% during the study period. This divergence likely results from combined factors including climate change impacts (26, 45), limited air conditioning availability (46), inadequate housing infrastructure (46, 47), and healthcare system constraints (26, 45). Period effect analysis quantified the remarkable protection achieved in high SDI regions, where RR for temperature-related IHD burden declined dramatically from 4.26 to 0.43, reflecting decades of investment in temperature adaptation infrastructure (26). Birth cohort analysis revealed progressive risk reductions across successive cohorts, most pronounced in high SDI regions (34, 48). These improvements in high SDI regions likely result from a combination of improved building standards (36), enhanced public awareness of temperature-related health risks (49), better clinical management of temperature-sensitive cardiovascular conditions (48), and stronger healthcare infrastructure (34, 48). Finally, we observed significant gender disparities in temperature-related IHD burden, with males consistently showing higher rates, particularly in high-temperature exposure scenarios (26, 34). This differential vulnerability reflects sex-specific physiological differences in thermoregulation (27, 28), occupational exposure patterns with males more frequently engaged in outdoor labor (26, 48), and underlying cardiovascular risk profile differences (34, 50, 51). Interestingly, these patterns vary by temperature type and geography, with men experiencing higher burden under heat exposure in tropical regions, while women show greater vulnerability to cold-related IHD in regions like Eastern Europe (26).

Based on these geographical and demographic patterns, targeted interventions are essential. Regarding non-optimal temperature exposure patterns, interventions in Middle Eastern (Saudi Arabia, UAE), North African (Egypt, Algeria), and Central Asian (Kazakhstan, Uzbekistan) regions necessitate enhanced temperature monitoring systems, public health planning that integrates climate adaptation strategies, and improved building insulation standards. Correspondingly, healthcare protocols should evolve to address cardiovascular management during extreme temperature events, alongside patient education regarding temperature-related cardiovascular risks.

### 4.5 Lead exposure-related IHD burden

Lead exposure presents a persistent environmental hazard for cardiovascular health. The analysis found that global age-standardized lead-attributable IHD deaths rates decreased modestly by 10.11% between 1990 and 2021, with DALYs rates declining by 16.58%. Lead affects cardiovascular function through multiple mechanisms, including hypertensive effects (9), oxidative stress induction (9, 23), endothelial damage (9), and myocardial function influences (23). Our geographical analysis revealed unexpected patterns of lead-attributable IHD burden, with substantial burden observed in regions with historically high industrial activity, including North America, Australia, and parts of Europe. APC analysis demonstrated that lead-related IHD burden increases with age, with notably elevated rates in the 25–29 age group. Period effect analysis showed significant improvements in high SDI regions while low SDI regions showed limited progress. Cohort analysis revealed substantially higher RR in earlier birth cohorts, with high SDI regions demonstrating the most pronounced decline in cohort effects. Gender analysis showed consistently higher lead-attributable IHD burden in males. The geographical distribution of lead-attributable IHD burden revealed unexpected patterns, with substantial burden observed in regions with historically high industrial activity (North America, Australia, and parts of Europe). This distribution likely reflects the legacy of prolonged industrial lead use, with environmental persistence creating ongoing exposure pathways despite reduced contemporary emissions (9). APC analysis revealed distinctive age-specific patterns in lead-related IHD, with significantly elevated deaths rates in the 25–29 age group, supporting the hypothesis that childhood lead exposure may influence adult cardiovascular health through developmental impacts (9, 10, 52). Burden peaked in older age groups across all SDI regions, with low-middle SDI regions exhibiting the highest rates, potentially reflecting cumulative exposure effects and age-related cardiovascular vulnerability to toxic metals. Period effect analysis demonstrated modest but consistent global improvements, with high SDI regions experiencing the most significant progress, reflecting successful lead abatement policies, including leaded gasoline phase-out, lead paint restrictions, and industrial emissions controls (9, 53). We identified concerning trends in middle and low SDI regions, where lead-attributable IHD deaths rates increased by 4.51 and 5.98%, respectively, during the study period, suggesting emerging exposure sources potentially related to ongoing industrialization without adequate regulatory safeguards (54), increasing vehicular emissions (55), and continued use of lead-containing products (52). Cohort analysis revealed substantial risk reductions across successive birth cohorts in high SDI regions, with deaths rate ratios declining from 16.35 in the 1900–1904 cohort to approximately 1.0 in recent cohorts, reflecting generational benefits of lead exposure reduction policies (9). Finally, our gender analysis demonstrated consistently higher lead-attributable IHD burden in males across all age groups and SDI regions. This differential burden likely reflects occupational exposure disparities, with males disproportionately engaged in high-exposure industries including mining, manufacturing, and construction (9). We suggest that the relatively slower improvement in female lead-related IHD burden may indicate the importance of residential environmental and non-occupational exposure pathways in gender disparities.

Given these distinctive exposure patterns and differential impacts, targeted strategies are necessary. In addressing lead exposure patterns, environmental monitoring must be strengthened in regions with historical industrial activity (North America, Australia, parts of Europe), while emissions regulation in rapidly industrializing middle SDI regions requires substantial improvement. For clinical practice, incorporation of lead exposure history into cardiovascular risk assessments for young adults (25–29 age group) is warranted, alongside public awareness campaigns targeting lead exposure sources, particularly in households with children.

### 4.6 Limitations and future directions

Despite its contributions, this study has three notable limitations that suggest specific directions for future research. Initially, as a secondary analysis of GBD estimates, our study inherits the uncertainties inherent in the GBD modeling framework, with relatively wide confidence intervals reflecting combined uncertainty from limited data availability, model selection, and parameter estimation. Consequently, expanding environmental monitoring networks in resource-limited regions would reduce estimation uncertainty and improve data quality for future burden assessments. Additionally, the GBD framework’s approach of analyzing each risk factor independently through comparative risk assessment prevents assessment of potential synergistic or antagonistic effects between particulate matter, temperature, and lead exposures. Therefore, future research should investigate synergistic effects between multiple environmental exposures using advanced statistical methods to provide more comprehensive insights into their combined cardiovascular impacts. Finally, relying on modeled estimates rather than primary patient data may affect the precision of burden estimates, particularly in regions with limited vital registration systems or environmental monitoring networks. Accordingly, integrating clinical registry data with environmental monitoring, particularly in data-sparse regions, would improve burden estimates and enable more targeted interventions based on local exposure patterns and population characteristics.

## 5 Conclusion

This comprehensive analysis reveals significant yet uneven reductions in environmentally-attributable IHD burden during 1990–2021. Particulate matter pollution remains the predominant environmental contributor, with South Asia, the Middle East, and North Africa bearing the heaviest burden. Similarly, non-optimal temperature exposure affects primarily the Middle East, North Africa, and Central Asia, while lead exposure shows distinctive patterns in historically industrialized regions. Across all environmental factors, males consistently demonstrate higher environmental-related IHD burden than females, and APC analysis indicates exponential increases with age, particularly in low-middle SDI regions. While this study is limited by reliance on modeled estimates and inability to assess interactions between environmental factors, the findings provide valuable evidence for policy formulation. Based on these patterns, targeted interventions are essential. Most critically, low SDI regions require integration of environmental health into development strategies, while high-burden regions necessitate robust air quality monitoring systems. Additionally, climate-adaptive health planning should be implemented for temperature-related risks, and environmental monitoring must be strengthened in both industrialized and rapidly industrializing regions. Throughout these efforts, special attention should be given to gender-specific occupational protections and age-appropriate interventions for vulnerable populations.

## Data Availability

The original contributions presented in the study are included in the article/Supplementary material, further inquiries can be directed to the corresponding author.
