# Supplementary figures and images for: Global ischemic heart disease burden attributable to environmental risk factors, 1990–2021: an Age-Period-Cohort analysis
Source: Front Public Health. 2025 Aug 1;13:1622108. doi: 10.3389/fpubh.2025.1622108 (PMC12354537; doi:10.3389/fpubh.2025.1622108)

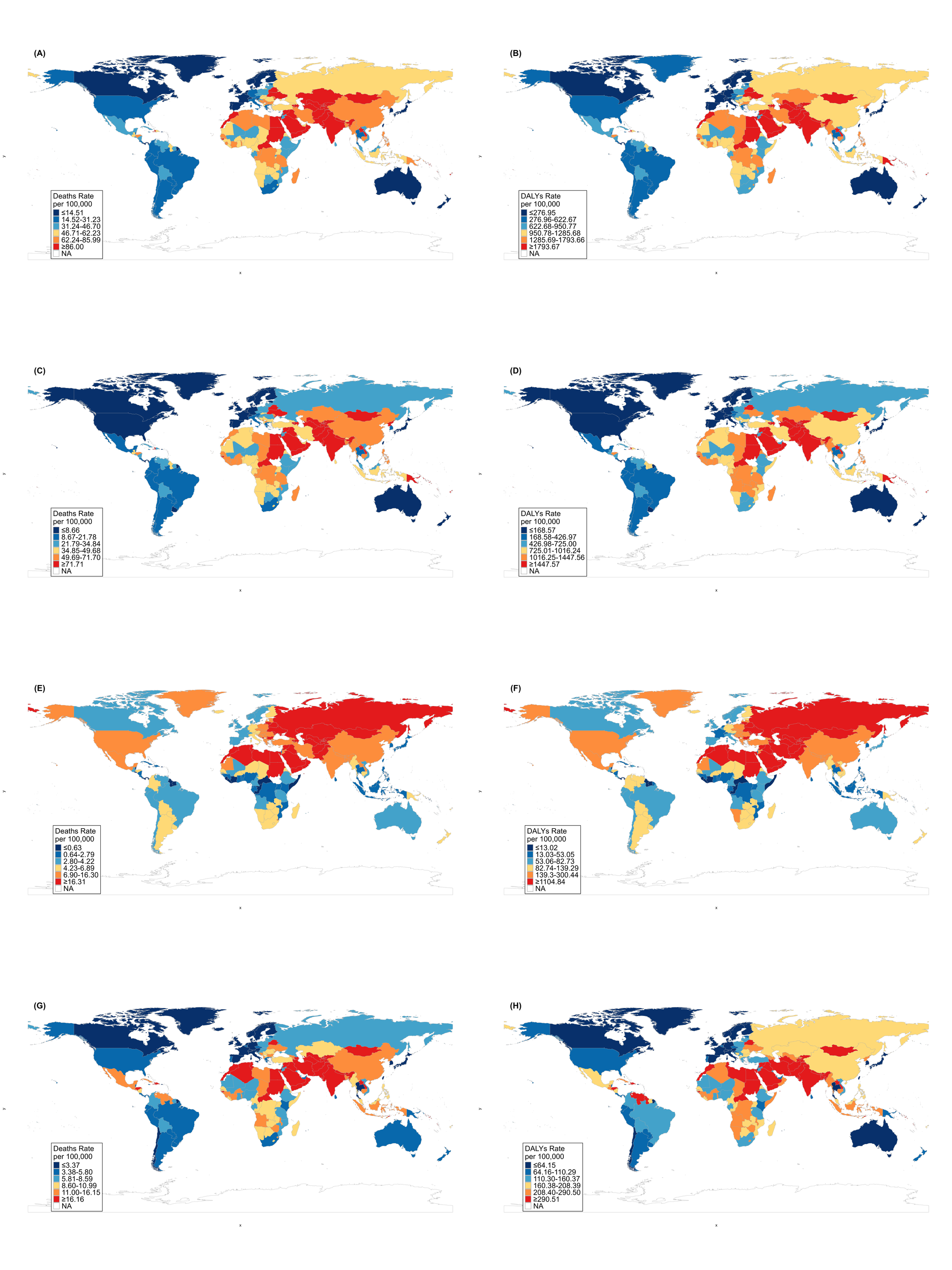

Supplement: Supplementary file 1 [file Image_1.TIFF]

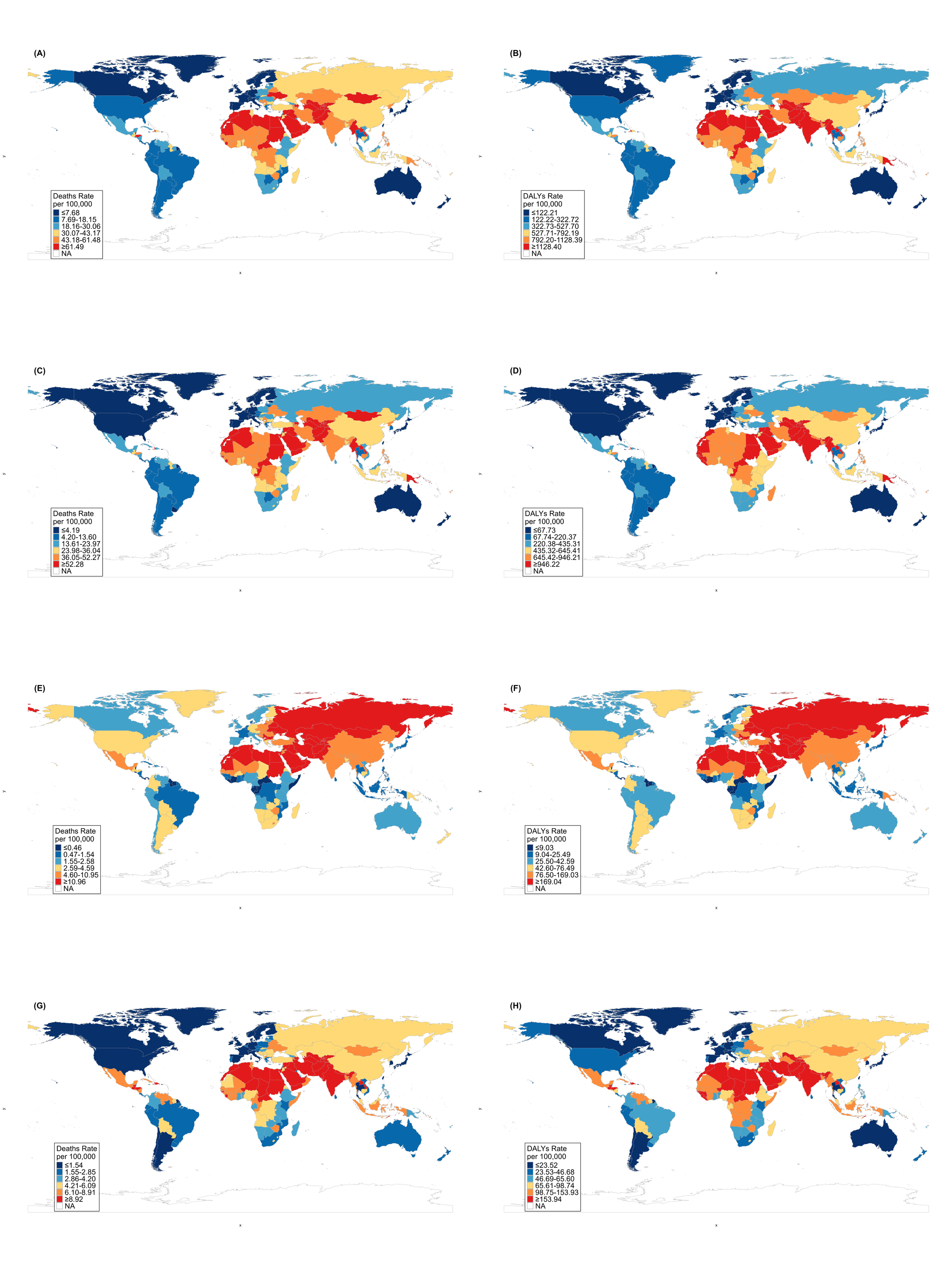

Supplement: Supplementary file 2 [file Image_2.TIFF]

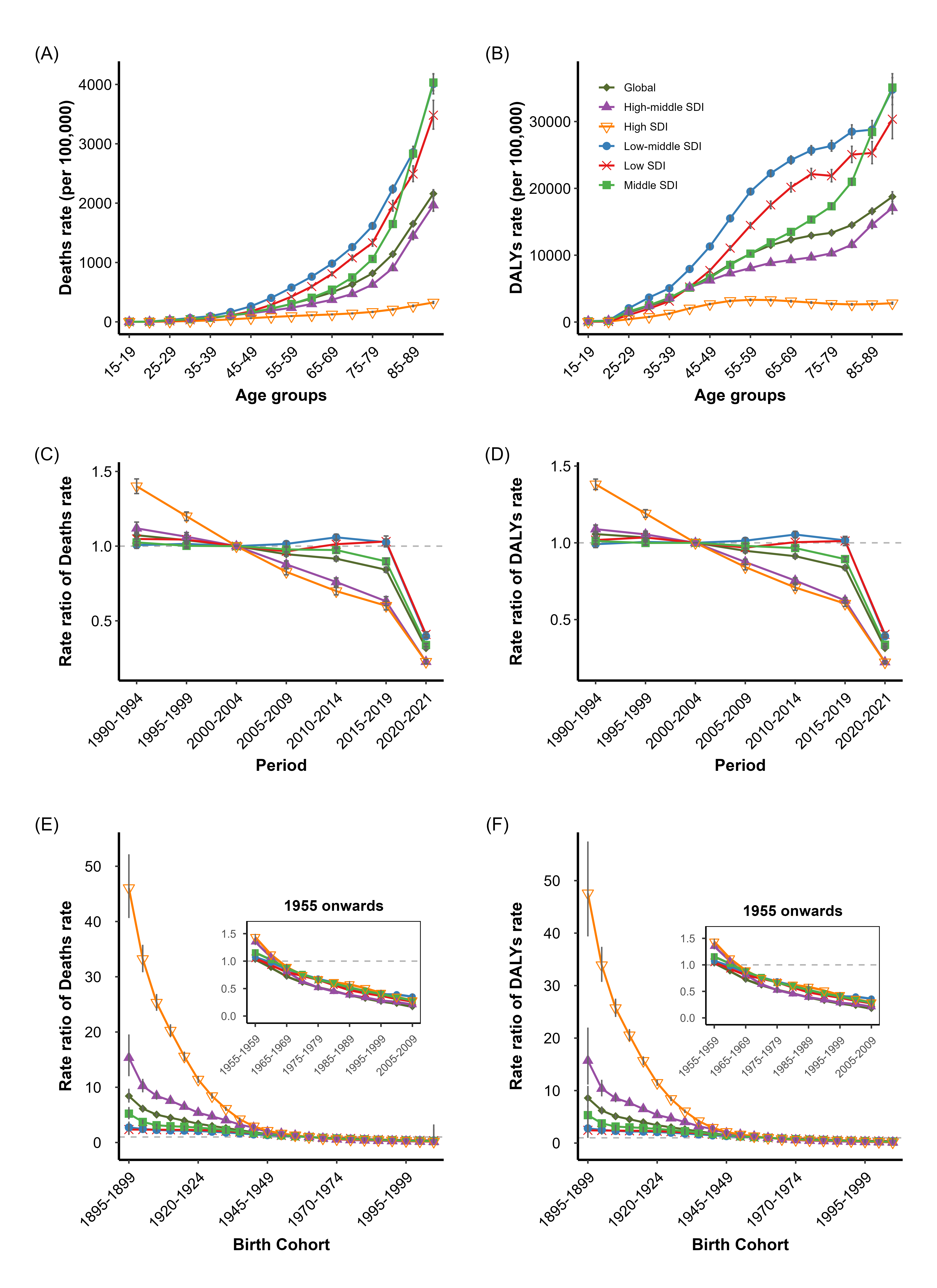

Supplement: Supplementary file 3 [file Image_3.TIFF]

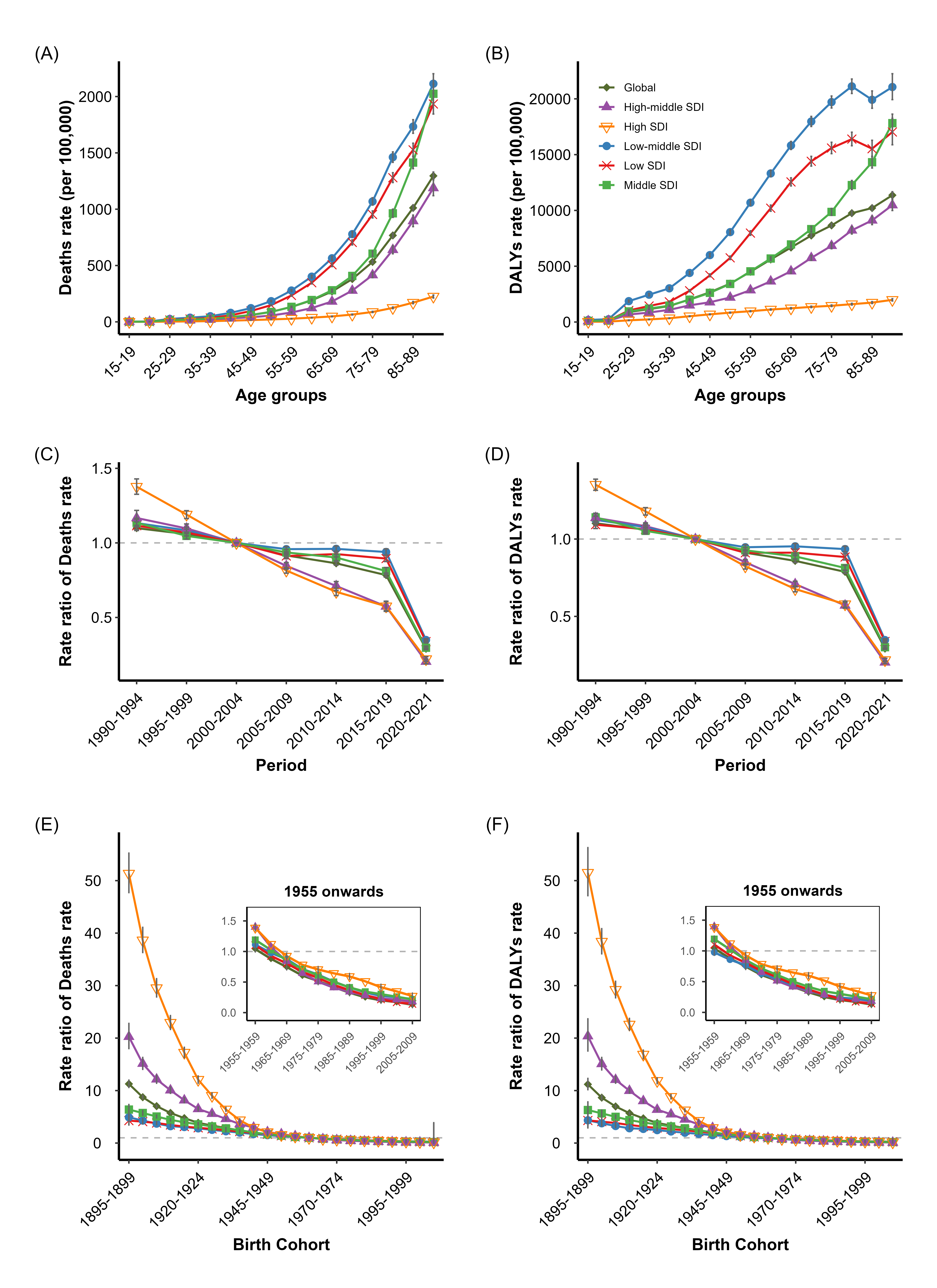

Supplement: Supplementary file 4 [file Image_4.TIFF]

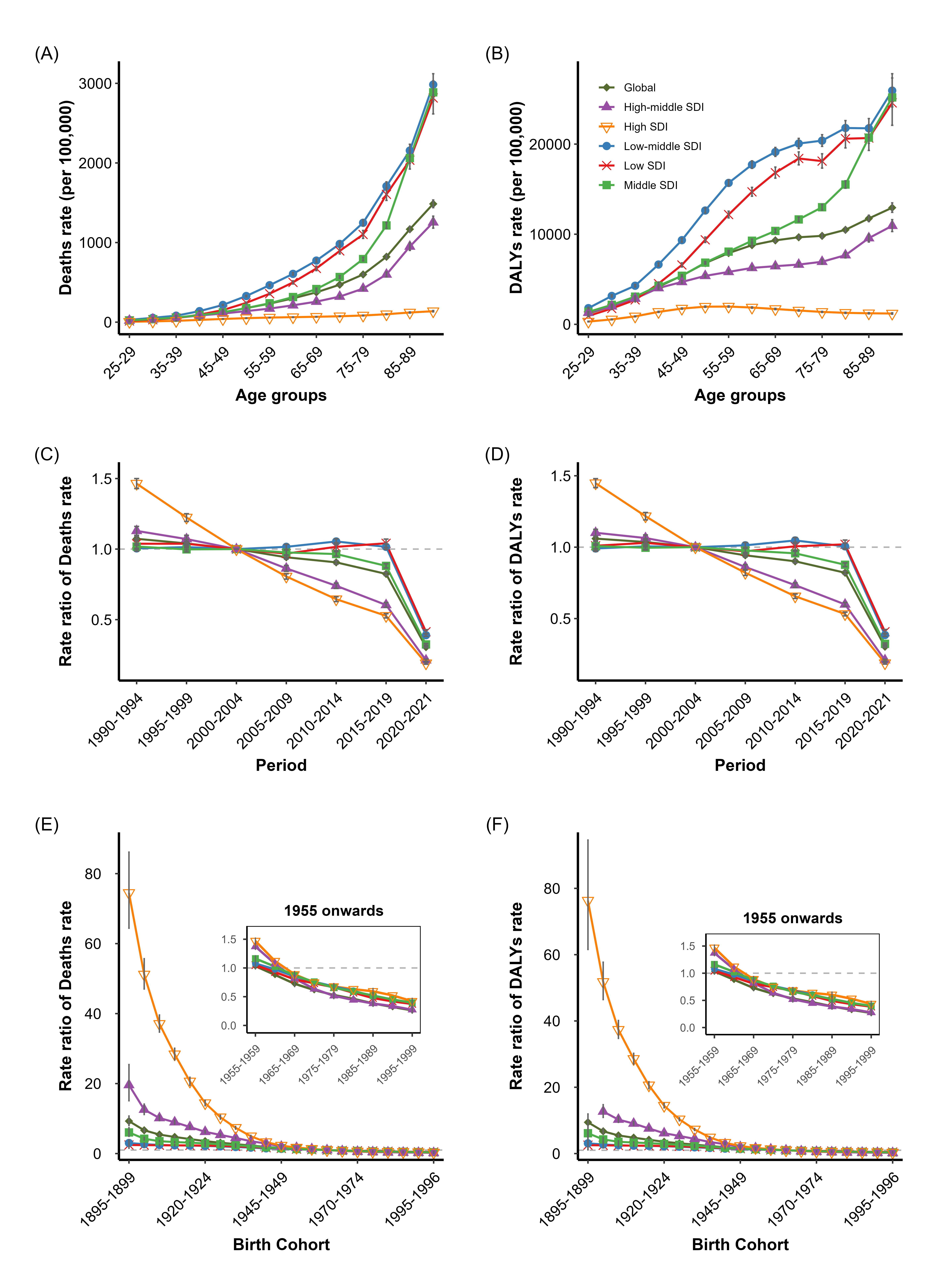

Supplement: Supplementary file 5 [file Image_5.TIFF]

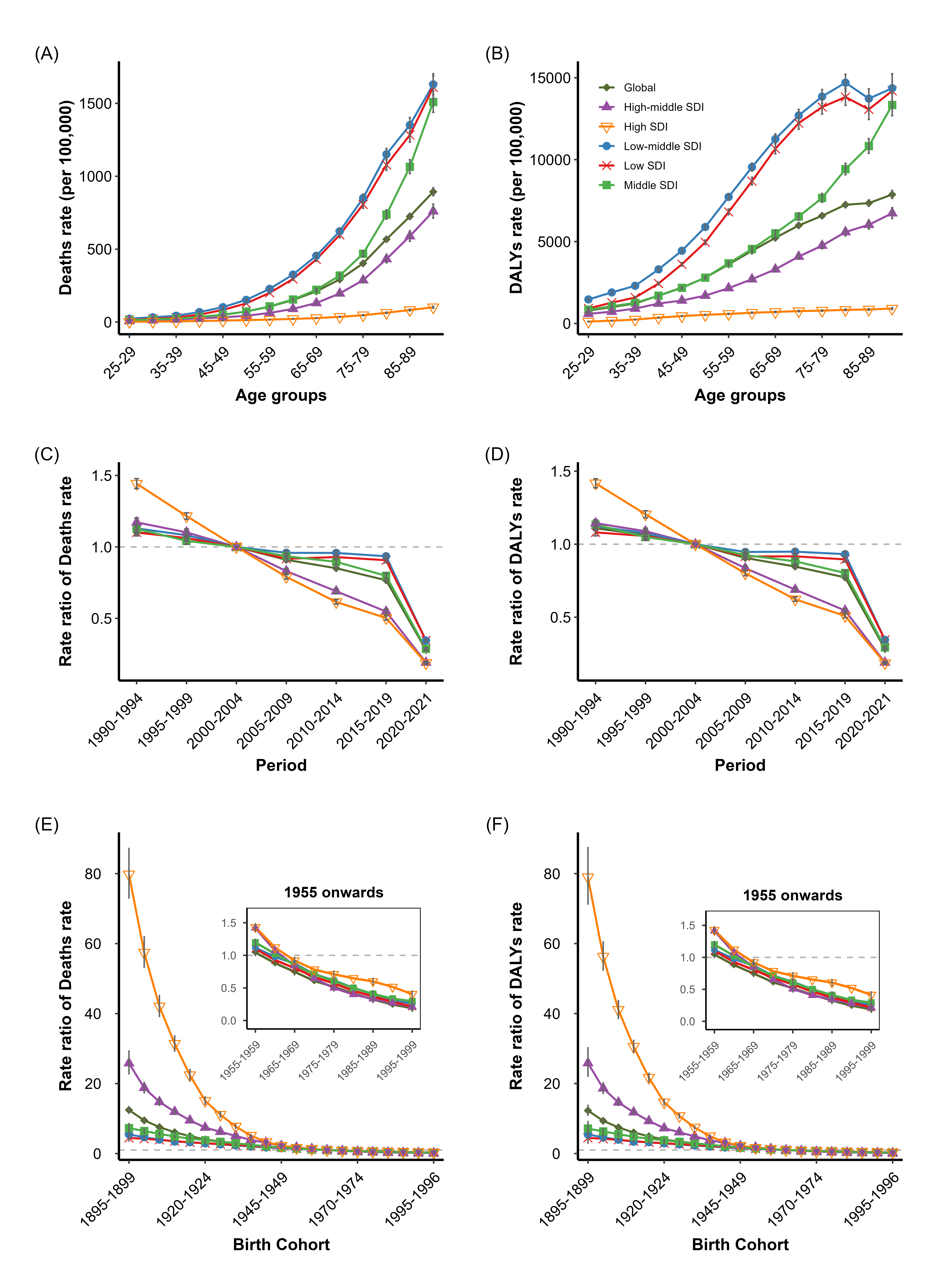

Supplement: Supplementary file 6 [file Image_6.TIFF]

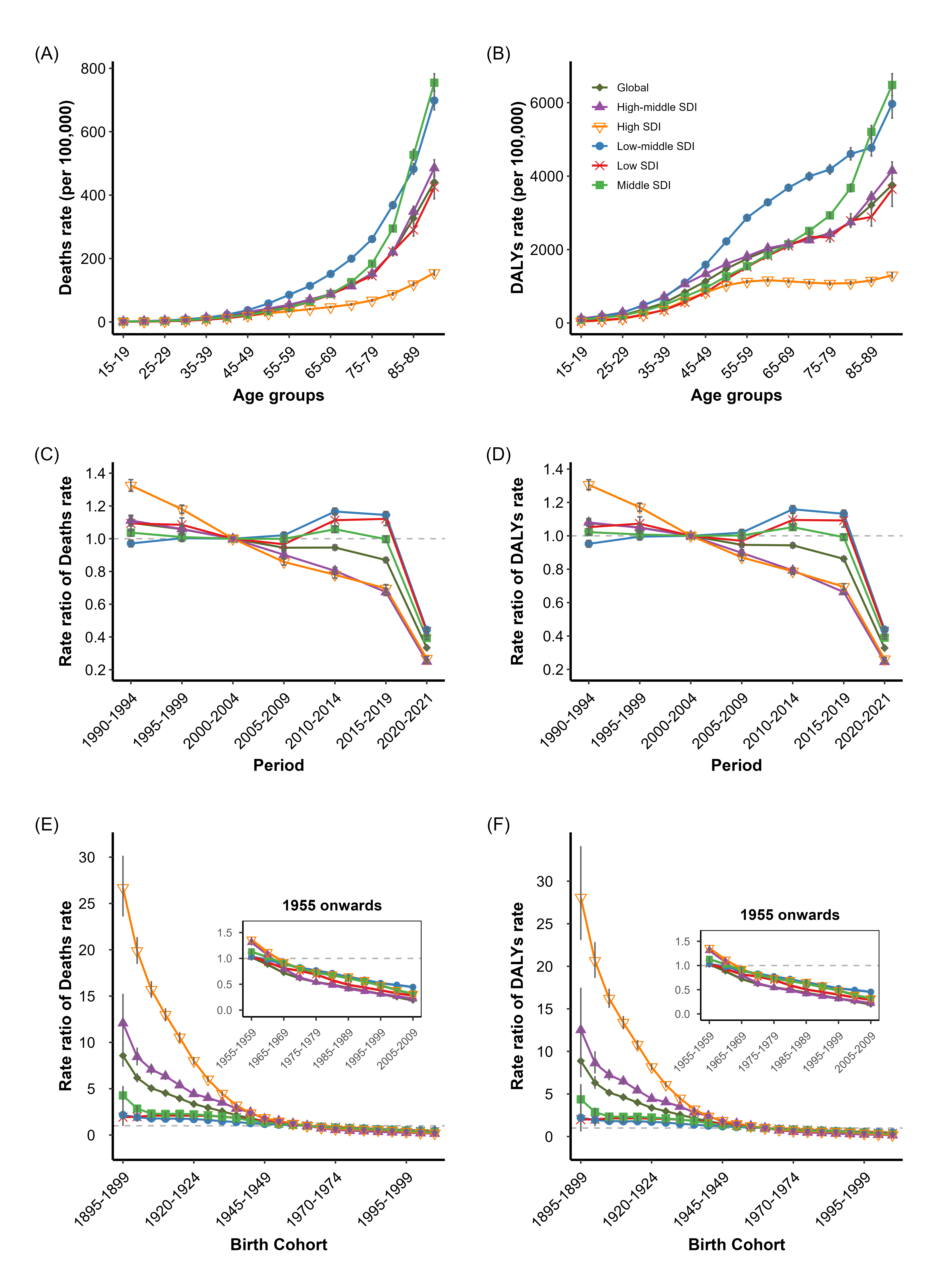

Supplement: Supplementary file 7 [file Image_7.TIFF]

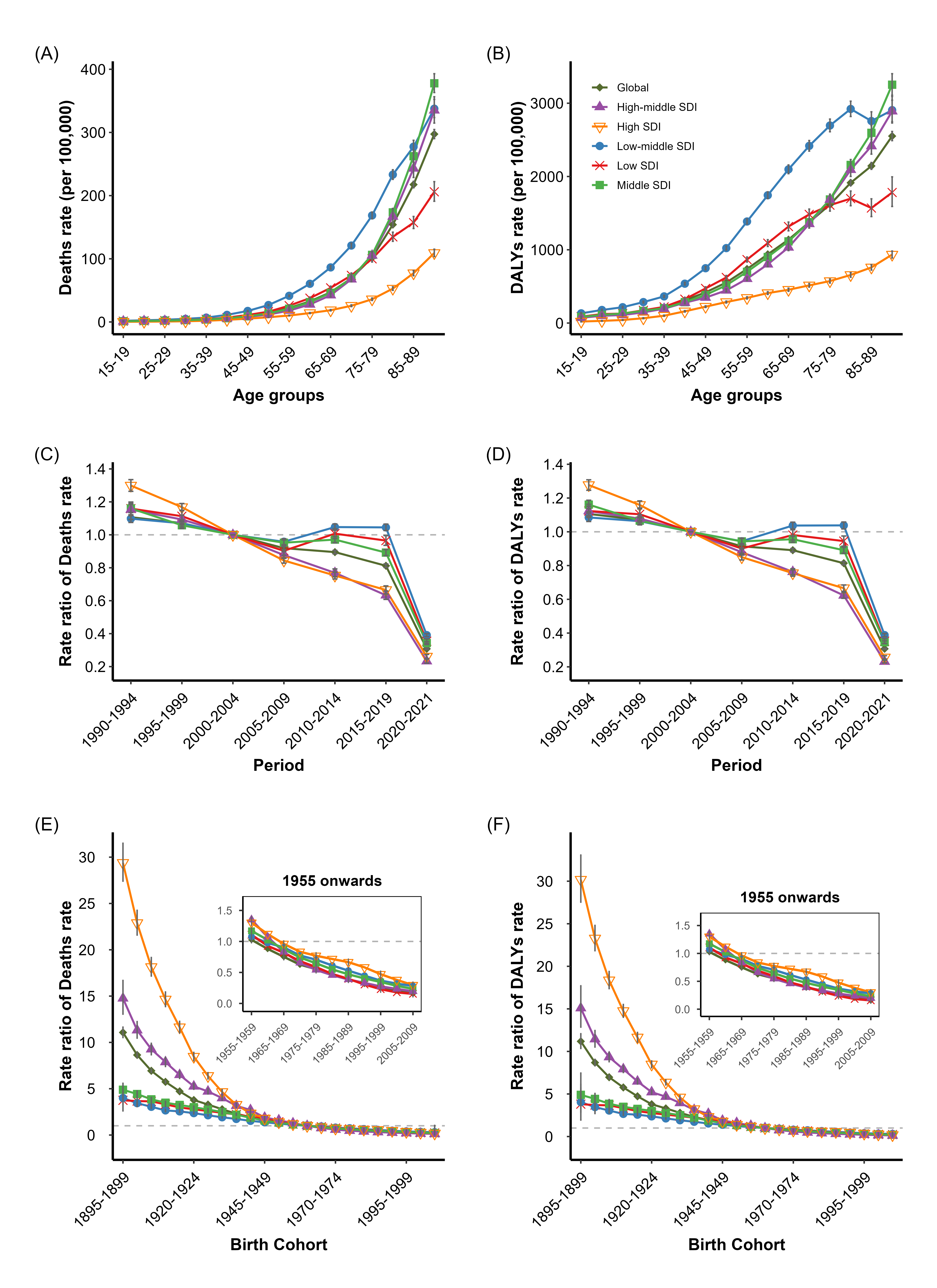

Supplement: Supplementary file 8 [file Image_8.TIFF]

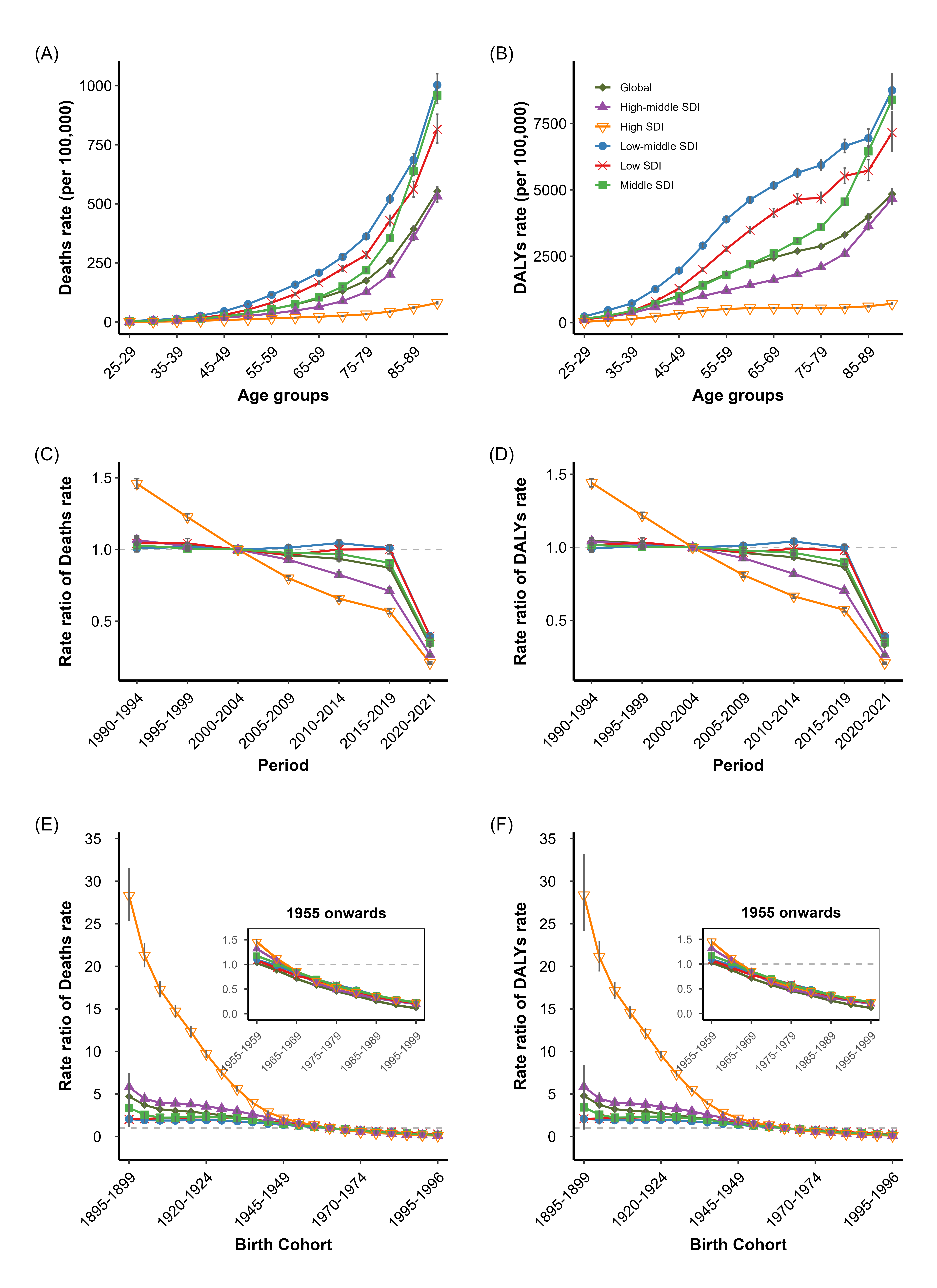

Supplement: Supplementary file 9 [file Image_9.TIFF]

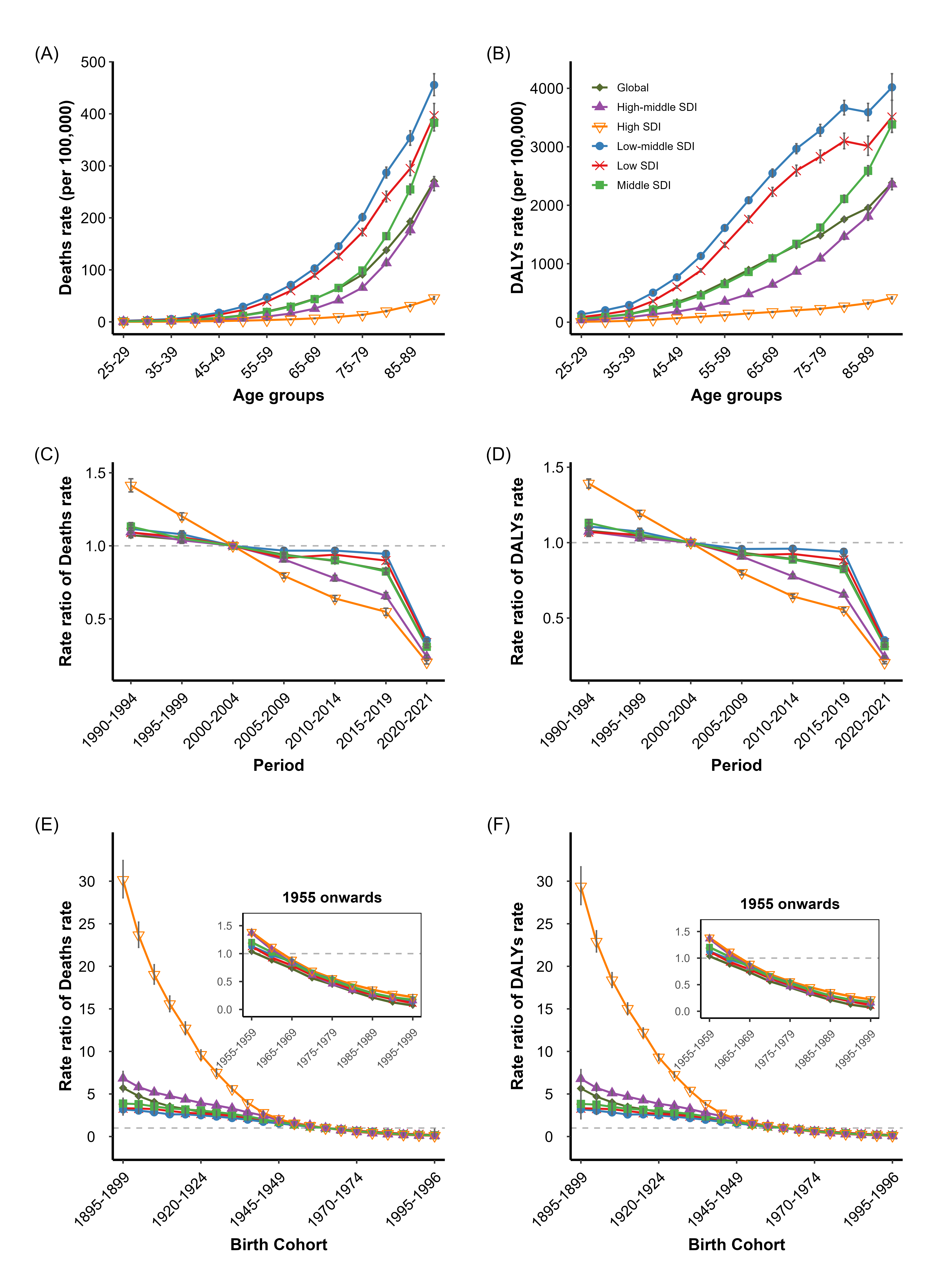

Supplement: Supplementary file 10 [file Image_10.TIFF]
