# Supplementary material for: Global ischemic heart disease burden attributable to environmental risk factors, 1990–2021: an Age-Period-Cohort analysis
Source: Front Public Health. 2025 Aug 1;13:1622108. doi: 10.3389/fpubh.2025.1622108 (PMC12354537; doi:10.3389/fpubh.2025.1622108)
Supplement: Supplementary file 11 [file Table_1.DOCX]

Supplementary Material

**Supplementary TABLE 1** STROBE Statement Checklist

| Item | Recommendation | Page |
| --- | --- | --- |
| Title and abstract | | |
| 1 | (a) Indicate the study's design with a commonly used term in the title or the abstract | 1 |
|  | (b) Provide in the abstract an informative and balanced summary of what was done and what was found | 1 |
| Introduction | | |
| 2 | Explain the scientific background and rationale for the investigation being reported | 2-3 |
| 3 | State specific objectives, including any prespecified hypotheses | 3 |
| Methods | | |
| 4 | Present key elements of study design early in the paper | 4 |
| 5 | Describe the setting, locations, and relevant dates, including periods of recruitment, exposure, follow-up, and data collection | 4 |
| 6 | (a) Cross-sectional study—Give the eligibility criteria, and the sources and methods of selection of participants | 4 |
|  | (b) For matched studies, give matching criteria and number of exposed and unexposed | 4 |
| 7 | Clearly define all outcomes, exposures, predictors, potential confounders, and effect modifiers. Give diagnostic criteria, if applicable | N/A |
| 8 | For each variable of interest, give sources of data and details of methods of assessment (measurement). Describe comparability of assessment methods if there is more than one group | 5-6 |
| 9 | Describe any efforts to address potential sources of bias | 4-6 |
| 10 | Explain how the study size was arrived at | 7 |
| 11 | Explain how quantitative variables were handled in the analyses. If applicable, describe which groupings were chosen and why | 4-5,7 |
| 12 | (a) Describe all statistical methods, including those used to control for confounding | 7-8 |
|  | (b) Describe any methods used to examine subgroups and interactions | 7-8 |
|  | (c) Explain how missing data were addressed | 8 |
|  | (d) Cross-sectional study—If applicable, describe analytical methods taking account of sampling strategy | 4,7-8 |
|  | (e) Describe any sensitivity analyses | 8 |
| Results | | |
| 13 | (a) Report numbers of individuals at each stage of study—eg numbers potentially eligible, examined for eligibility, confirmed eligible, included in the study, completing follow-up, and analysed | 9, TABLE 1 |
|  | (b) Give reasons for non-participation at each stage | N/A |
|  | (c) Consider use of a flow diagram | N/A |
| 14 | (a) Give characteristics of study participants (eg demographic, clinical, social) and information on exposures and potential confounders | 9, TABLE 1 |
|  | (b) Indicate number of participants with missing data for each variable of interest | 4 |
|  | (c) Cohort study—Summarise follow-up time (eg, average and total amount) | N/A |
| 15 | Cross-sectional study—Report numbers of outcome events or summary measures | 9-14, TABLE 1, FIGURE 1-5 |
| 16 | (a) Give unadjusted estimates and, if applicable, confounder-adjusted estimates and their precision (eg, 95% confidence interval). Make clear which confounders were adjusted for and why they were included | 9-14, TABLE 1 |
|  | (b) Report category boundaries when continuous variables were categorized | 4-5 |
|  | (c) If relevant, consider translating estimates of relative risk into absolute risk for a meaningful time period | 9-14 |
| 17 | Report other analyses done—eg analyses of subgroups and interactions, and sensitivity analyses | 9-14, FIGURE 2-5 |
| Discussion | | |
| 18 | Summarise key results with reference to study objectives | 15 |
| 19 | Discuss limitations of the study, taking into account sources of potential bias or imprecision. Discuss both direction and magnitude of any potential bias | 19 |
| 20 | Give a cautious overall interpretation of results considering objectives, limitations, multiplicity of analyses, results from similar studies, and other relevant evidence | 15-19 |
| 21 | Discuss the generalisability (external validity) of the study results | 18-19 |
| Other information | | |

**Supplementary TABLE 1. (Continued)**

| Item | Recommendation | Page |
| --- | --- | --- |
| 22 | Give the source of funding and the role of the funders for the present study and, if applicable, for the original study on which the present article is based | N/A  (Not mentioned in provided manuscript) |

**Supplementary TABLE 2** GATHER Statement Checklist

| Item | Checklist item | Page |
| --- | --- | --- |
| Objectives and funding | | |
| 1 | Define the indicator(s), populations (including age, sex, and geographic entities), and time period(s) for which estimates were made | 1,4 |
| 2 | List the funding sources for the work | N/A  (Not mentioned in provided manuscript) |
| Data Inputs | | |
| For all data inputs from multiple sources that are synthesized as part of the study: | | |
| 3 | Describe how the data were identified and how the data were accessed | 4 |
| 4 | Specify the inclusion and exclusion criteria. Identify all ad-hoc exclusions | 4-5 |
| 5 | Provide information on all included data sources and their main characteristics. For each data source used, report reference information or contact name/institution, population represented, data collection method, year(s) of data collection, sex and age range, diagnostic criteria or measurement method, and sample size, as relevant | 4-6 |
| 6 | Identify and describe any categories of input data that have potentially important biases (e.g., based on characteristics listed in item 5) | 19 |
| For data inputs that contribute to the analysis but were not synthesized as part of the study: | | |
| 7 | Describe and give sources for any other data inputs | 4, References 16-17 |
| For all data inputs: | | |
|  | Provide all data inputs in a file format from which data can be efficiently extracted (e.g., a spreadsheet rather than a PDF), including all relevant meta-data listed in item 5. For any data inputs that cannot be shared because of ethical or legal reasons, such as third-party ownership, provide a contact name or the name of the institution that retains the right to the data | 5  (Data publicly available from GBD database) |
| Data analysis | | |
| 9 | Provide a conceptual overview of the data analysis method. A diagram may be helpful | 7-8 |
| 10 | Provide a detailed description of all steps of the analysis, including mathematical formulae. This description should cover, as relevant, data cleaning, data pre-processing, data adjustments and weighting of data sources, and mathematical or statistical model(s) | 6-8 |
| 11 | Describe how candidate models were evaluated and how the final model(s) were selected | 7-8 |
| 12 | Provide the results of an evaluation of model performance, if done, as well as the results of any relevant sensitivity analysis | 8, 19 |
| 13 | Describe methods for calculating uncertainty of the estimates. State which sources of uncertainty were, and were not, accounted for in the uncertainty analysis | 6-7, 19 |
| 14 | State how analytic or statistical source code used to generate estimates can be accessed | 8  (R version 4.4.3, APC Web Tool) |
| Results and Discussion | | |
| 15 | Provide published estimates in a file format from which data can be efficiently extracted | TABLE 1, FIGURES 1-5 |
| 16 | Report a quantitative measure of the uncertainty of the estimates (e.g. uncertainty intervals) | 9-14, TABLE 1  (95% CI reported) |
| 17 | Interpret results in light of existing evidence. If updating a previous set of estimates, describe the reasons for changes in estimates | 15-18 |
| 18 | Discuss limitations of the estimates. Include a discussion of any modelling assumptions or data limitations that affect interpretation of the estimates | 19 |

**Supplementary TABLE 3** Quintiles of different SDI ^a^ groups.

| SDI ^a^ quintile | lower bound | upper bound |
| --- | --- | --- |
| Low SDI ^a^ | 0 | 0.46581580319161997 |
| Low-middle SDI ^a^ | 0.46581580319161997 | 0.6188294452454329 |
| Middle SDI ^a^ | 0.6188294452454329 | 0.7119746219361235 |
| High-middle SDI ^a^ | 0.7119746219361235 | 0.8102959891918925 |
| High SDI ^a^ | 0.8102959891918925 | 1 |

**Note:** Values shown represent lower and upper bounds of each SDI quintile.

^a^ SDI = Socio-demographic Index, range from 0 to 1, with higher values indicating higher socio-demographic development.

**Supplementary TABLE 4** Countries and territories of different SDI ^a^ groups in 1990 and 2021.

| Countries and territories | 1990 | 2021 |
| --- | --- | --- |
| Afghanistan | Low SDI | Low SDI |
| Albania | Low-middle SDI | Middle SDI |
| Algeria | Low SDI | Middle SDI |
| American Samoa | Low-middle SDI | High-middle SDI |
| Andorra | High-middle SDI | High SDI |
| Angola | Low SDI | Low-middle SDI |
| Antigua and Barbuda | Low-middle SDI | High-middle SDI |
| Argentina | Low-middle SDI | High-middle SDI |
| Armenia | Low-middle SDI | Middle SDI |
| Australia | High-middle SDI | High SDI |
| Austria | High-middle SDI | High SDI |
| Azerbaijan | Low-middle SDI | Middle SDI |
| Bahamas | Middle SDI | High-middle SDI |
| Bahrain | Low-middle SDI | High-middle SDI |
| Bangladesh | Low SDI | Low-middle SDI |
| Barbados | Middle SDI | High-middle SDI |
| Belarus | Middle SDI | High-middle SDI |
| Belgium | High-middle SDI | High SDI |
| Belize | Low SDI | Low-middle SDI |
| Benin | Low SDI | Low SDI |
| Bermuda | Middle SDI | High SDI |
| Bhutan | Low SDI | Low-middle SDI |
| Bolivia (Plurinational State of) | Low SDI | Low-middle SDI |
| Bosnia and Herzegovina | Low-middle SDI | High-middle SDI |
| Botswana | Low SDI | Middle SDI |
| Brazil | Low-middle SDI | Middle SDI |
| Brunei Darussalam | Middle SDI | High-middle SDI |
| Bulgaria | Middle SDI | High-middle SDI |
| Burkina Faso | Low SDI | Low SDI |
| Burundi | Low SDI | Low SDI |
| Cabo Verde | Low SDI | Low-middle SDI |
| Cambodia | Low SDI | Low-middle SDI |
| Cameroon | Low SDI | Low-middle SDI |
| Canada | High-middle SDI | High SDI |
| Central African Republic | Low SDI | Low SDI |
| Chad | Low SDI | Low SDI |
| Chile | Low-middle SDI | High-middle SDI |
| China | Low SDI | High-middle SDI |
| Colombia | Low-middle SDI | Middle SDI |
| Comoros | Low SDI | Low-middle SDI |
| Congo | Low SDI | Low-middle SDI |
| Cook Islands | Low-middle SDI | High-middle SDI |
| Costa Rica | Low-middle SDI | Middle SDI |
| Côte d'Ivoire | Low SDI | Low SDI |

**Supplementary TABLE 4. (Continued)**

| Countries and territories | 1990 | 2021 |
| --- | --- | --- |
| Croatia | Middle SDI | High-middle SDI |
| Cuba | Low-middle SDI | Middle SDI |
| Cyprus | Middle SDI | High SDI |
| Czechia | Middle SDI | High SDI |
| Democratic People's Republic of Korea | Low-middle SDI | Low-middle SDI |
| Democratic Republic of the Congo | Low SDI | Low SDI |
| Denmark | High-middle SDI | High SDI |
| Djibouti | Low SDI | Low-middle SDI |
| Dominica | Low-middle SDI | High-middle SDI |
| Dominican Republic | Low SDI | Middle SDI |
| Ecuador | Low-middle SDI | Middle SDI |
| Egypt | Low SDI | Low-middle SDI |
| El Salvador | Low SDI | Low-middle SDI |
| Equatorial Guinea | Low SDI | Middle SDI |
| Eritrea | Low SDI | Low SDI |
| Estonia | Middle SDI | High SDI |
| Eswatini | Low SDI | Low-middle SDI |
| Ethiopia | Low SDI | Low SDI |
| Fiji | Low-middle SDI | Middle SDI |
| Finland | High-middle SDI | High SDI |
| France | High-middle SDI | High SDI |
| Gabon | Low SDI | Middle SDI |
| Gambia | Low SDI | Low SDI |
| Georgia | Middle SDI | High-middle SDI |
| Germany | High SDI | High SDI |
| Ghana | Low SDI | Low-middle SDI |
| Greece | Middle SDI | High-middle SDI |
| Greenland | High-middle SDI | High SDI |
| Grenada | Low SDI | Middle SDI |
| Guam | Middle SDI | High-middle SDI |
| Guatemala | Low SDI | Low-middle SDI |
| Guinea | Low SDI | Low SDI |
| Guinea-Bissau | Low SDI | Low SDI |
| Guyana | Low SDI | Middle SDI |
| Haiti | Low SDI | Low SDI |
| Honduras | Low SDI | Low-middle SDI |
| Hungary | Middle SDI | High-middle SDI |
| Iceland | High-middle SDI | High SDI |
| India | Low SDI | Low-middle SDI |
| Indonesia | Low SDI | Middle SDI |
| Iran (Islamic Republic of) | Low SDI | Middle SDI |
| Iraq | Low SDI | Middle SDI |
| Ireland | High-middle SDI | High SDI |
| Israel | Middle SDI | High-middle SDI |
| Italy | Middle SDI | High-middle SDI |
| Jamaica | Low-middle SDI | Middle SDI |
| Japan | High-middle SDI | High SDI |
| Jordan | Low-middle SDI | High-middle SDI |
| Kazakhstan | Low-middle SDI | High-middle SDI |
| Kenya | Low SDI | Low-middle SDI |
| Kiribati | Low SDI | Low-middle SDI |
| Kuwait | Middle SDI | High SDI |
| Kyrgyzstan | Low-middle SDI | Low-middle SDI |
| Lao People's Democratic Republic | Low SDI | Low-middle SDI |
| Latvia | Middle SDI | High SDI |
| Lebanon | Low-middle SDI | High-middle SDI |
| Lesotho | Low SDI | Low-middle SDI |
| Liberia | Low SDI | Low SDI |
| Libya | Low-middle SDI | High-middle SDI |
| Lithuania | Middle SDI | High SDI |
| Luxembourg | High-middle SDI | High SDI |
| Madagascar | Low SDI | Low SDI |
| Malawi | Low SDI | Low SDI |
| Malaysia | Low-middle SDI | High-middle SDI |

**Supplementary TABLE 4. (Continued)**

| Countries and territories | 1990 | 2021 |
| --- | --- | --- |
| Maldives | Low SDI | Middle SDI |
| Mali | Low SDI | Low SDI |
| Malta | Middle SDI | High-middle SDI |
| Marshall Islands | Low SDI | Low-middle SDI |
| Mauritania | Low SDI | Low-middle SDI |
| Mauritius | Low-middle SDI | High-middle SDI |
| Mexico | Low-middle SDI | Middle SDI |
| Micronesia (Federated States of) | Low SDI | Low-middle SDI |
| Monaco | High SDI | High SDI |
| Mongolia | Low-middle SDI | Low-middle SDI |
| Montenegro | Middle SDI | High-middle SDI |
| Morocco | Low SDI | Low-middle SDI |
| Mozambique | Low SDI | Low SDI |
| Myanmar | Low SDI | Low-middle SDI |
| Namibia | Low SDI | Low-middle SDI |
| Nauru | Low-middle SDI | Middle SDI |
| Nepal | Low SDI | Low SDI |
| Netherlands | High-middle SDI | High SDI |
| New Zealand | High-middle SDI | High SDI |
| Nicaragua | Low SDI | Low-middle SDI |
| Niger | Low SDI | Low SDI |
| Nigeria | Low SDI | Low-middle SDI |
| Niue | Low-middle SDI | High-middle SDI |
| North Macedonia | Low-middle SDI | High-middle SDI |
| Northern Mariana Islands | Middle SDI | High-middle SDI |
| Norway | High-middle SDI | High SDI |
| Oman | Low SDI | High-middle SDI |
| Pakistan | Low SDI | Low-middle SDI |
| Palau | Middle SDI | High-middle SDI |
| Palestine | Low SDI | Middle SDI |
| Panama | Low-middle SDI | Middle SDI |
| Papua New Guinea | Low SDI | Low SDI |
| Paraguay | Low-middle SDI | Middle SDI |
| Peru | Low-middle SDI | Middle SDI |
| Philippines | Low-middle SDI | Middle SDI |
| Poland | Middle SDI | High SDI |
| Portugal | Low-middle SDI | High-middle SDI |
| Puerto Rico | Middle SDI | High SDI |
| Qatar | Middle SDI | High SDI |
| Republic of Korea | Middle SDI | High SDI |
| Republic of Moldova | Middle SDI | High-middle SDI |
| Romania | Middle SDI | High-middle SDI |
| Russian Federation | High-middle SDI | High-middle SDI |
| Rwanda | Low SDI | Low SDI |
| Saint Kitts and Nevis | Low-middle SDI | High-middle SDI |
| Saint Lucia | Low-middle SDI | Middle SDI |
| Saint Vincent and the Grenadines | Low-middle SDI | Middle SDI |
| Samoa | Low-middle SDI | Low-middle SDI |
| San Marino | High SDI | High SDI |
| Sao Tome and Principe | Low-middle SDI | Low-middle SDI |
| Saudi Arabia | Low-middle SDI | High SDI |
| Senegal | Low SDI | Low SDI |
| Serbia | Middle SDI | High-middle SDI |
| Seychelles | Low-middle SDI | High-middle SDI |
| Sierra Leone | Low SDI | Low SDI |
| Singapore | Middle SDI | High SDI |
| Slovakia | Middle SDI | High-middle SDI |
| Slovenia | High-middle SDI | High SDI |
| Solomon Islands | Low SDI | Low SDI |
| Somalia | Low SDI | Low SDI |
| South Africa | Low-middle SDI | Middle SDI |
| South Sudan | Low SDI | Low SDI |
| Spain | Middle SDI | High-middle SDI |
| Sri Lanka | Low-middle SDI | Middle SDI |

**Supplementary TABLE 4. (Continued)**

| Countries and territories | 1990 | 2021 |
| --- | --- | --- |
| Sudan | Low SDI | Low-middle SDI |
| Suriname | Low-middle SDI | Middle SDI |
| Sweden | High-middle SDI | High SDI |
| Switzerland | High SDI | High SDI |
| Syrian Arab Republic | Low-middle SDI | Middle SDI |
| Taiwan | Middle SDI | High SDI |
| Tajikistan | Low-middle SDI | Low-middle SDI |
| Thailand | Low-middle SDI | Middle SDI |
| Timor-Leste | Low SDI | Low SDI |
| Togo | Low SDI | Low SDI |
| Tokelau | Low-middle SDI | Middle SDI |
| Tonga | Low-middle SDI | Middle SDI |
| Trinidad and Tobago | Middle SDI | High-middle SDI |
| Tunisia | Low-middle SDI | Middle SDI |
| Türkiye | Low SDI | High-middle SDI |
| Turkmenistan | Low-middle SDI | Middle SDI |
| Tuvalu | Low SDI | Low-middle SDI |
| Uganda | Low SDI | Low SDI |
| Ukraine | Middle SDI | High-middle SDI |
| United Arab Emirates | Middle SDI | High SDI |
| United Kingdom | High SDI | High SDI |
| United Republic of Tanzania | Low SDI | Low SDI |
| United States of America | High-middle SDI | High SDI |
| United States Virgin Islands | Middle SDI | High SDI |
| Uruguay | Low-middle SDI | High-middle SDI |
| Uzbekistan | Low-middle SDI | Middle SDI |
| Vanuatu | Low SDI | Low-middle SDI |
| Venezuela (Bolivarian Republic of) | Low-middle SDI | Low-middle SDI |
| Viet Nam | Low SDI | Middle SDI |
| Yemen | Low SDI | Low SDI |
| Zambia | Low SDI | Low-middle SDI |
| Zimbabwe | Low SDI | Low-middle SDI |

**Note**: ^a^ SDI = socio-demographic index; Countries are categorized based on the SDI quintile thresholds defined in Supplementary Table 1.

**Supplementary TABLE 5** Environmental risk factors for IHD based on GBD ^b^ 2021.

| Risk Factor | Level | Definition | TMREL ^c^ | Health Impact (2021) |
| --- | --- | --- | --- | --- |
| Ambient particulate matter pollution | Level 4 | Population-weighted annual average mass concentration of particles with an aerodynamic diameter less than 2.5 micrometres in a cubic metre of air | 2.4-5.9 μg/m³ ^f^ | - Deaths rates: 29.88/100,000  (95% UI ^d^ 22.33-37.22)  - DALYs ^e^ rates: 638.48/100,000  (95% UI ^d^ 486.47-787.82) |
| Non-optimal temperature | Level 2 | Aggregate of the burden attributable to low and high temperatures, with effects above and below the TMREL ^b^ | 25.6°C (population-weighted mean) | - Deaths rates: 7.39/100,000  (95% UI ^d^ 5.57-10.44)  - DALYs ^e^ rates:146.03/100,000  (95% UI ^d^ 107.55-207.43) |
| Lead exposure | Level 3 | Two types: acute exposure, measured by micrograms of lead per decilitre of blood, and chronic exposure, measured by micrograms of lead per gram of bone | Blood lead: 0.016 μg/dL ^f^  Bone lead: age-specific | - Deaths rates: 7.11/100,000  (95% UI ^d^ -1.01-14.88)  - DALYs ^e^ rates: 138.57/100,000  (95% UI ^d^ -19.52-289.73) |

**Note:** ^a^ IHD = ischemic heart disease;

^b^ GBD = Global Burden of Disease.

^c^ TMREL = Theoretical Minimum Risk Exposure Level.

^d^ UI = Uncertainty Interval.

^e^ DALYs = Disability-Adjusted Life Years.

^f^ μg/m³ = Micrograms per cubic metre; μg/dL = Micrograms per decilitre.

**Supplementary TABLE 6** Full list of selected covariates for the CODEm ^a^ models in the IHD ^b^ estimation.

| Level | Covariate | Direction ^f^ |
| --- | --- | --- |
| 1 | Summary exposure value, IHD ^b^ | 1 |
|  | Systolic blood pressure (mm Hg) | 1 |
|  | Smoking prevalence | 1 |
|  | Cholesterol (total, mean per capita) | 1 |
| 2 | Mean BMI ^c^ | 1 |
|  | Elevation over 1500m (proportion) | −1 |
|  | Fasting plasma glucose | 1 |
|  | Outdoor pollution (PM2.5 ^d^) | 1 |
|  | Indoor air pollution | 1 |
|  | Healthcare access and quality index | −1 |
| 3 | Lag distributed income per capita (I$) | −1 |
|  | Summary exposure value, omega-3 | 1 |
|  | Summary exposure value, fruits | 1 |
|  | Summary exposure value, vegetables | 1 |
|  | Summary exposure value, nuts and seeds | 1 |
|  | Pulses/legumes (kcal/capita, unadjusted) | −1 |
|  | Summary exposure value, PUFA ^e^ (percent, adjusted) | 1 |
|  | Alcohol (litres per capita) | 1 |
|  | Trans fatty acid | 1 |

**Note:** a CODEm = Cause of Death Ensemble model;

^b^ IHD = ischemic heart disease;

^c^ BMI = body mass index;

^d^ PM2.5 = particulate matter with diameter less than 2.5 micrometers;

^e^ PUFA = polyunsaturated fatty acids.

^f^ Direction: "1" indicates positive association (higher values of the covariate are associated with higher risk of IHD deaths); "−1" indicates negative association (higher values of the covariate are associated with lower risk of IHD deaths).

**Supplementary TABLE 7** Sequelae for IHD ^a^ and their associated disability weights.

| Category | Sequela | Sequela definition | Lay description | Disability weight ^b^  (95% CI) |
| --- | --- | --- | --- | --- |
| Myocardial infarction due to IHD ^a^ | Acute myocardial infarction,3 to 28 days | Acute myocardial infarction, days 3-28 | Gets short of breath after heavy physical activity, and tires easily, but has no problems when at rest. The person has to take medication every day and has some anxiety. | 0.074  (0.049-0.105) |
|  | Acute myocardial infarction,1-2 days | Acute myocardial infarction, days 1-2 | Has severe chest pain that becomes worse with any physical activity. The person feels nauseous, short of breath, and very anxious. | 0.432  (0.288-0.579) |
| Angina due to IHD ^a^ | Asymptomatic angina due to IHD ^a^ | - | - | - |
|  | Mild angina due to IHD ^a^ | Angina pectoris, mild | Has chest pain that occurs with strenuous physical activity, such as running or lifting heavy objects. After a brief rest, the pain goes away. | 0.033  (0.020-0.052) |
|  | Moderate angina due to IHD a | Angina pectoris, moderate | Has chest pain that occurs with moderate physical activity, such as walking uphill or more than half a kilometer (around a quarter-mile) on level ground. After a brief rest, the pain goes away. | 0.080  (0.052-0.113) |
|  | Severe angina due to IHD ^a^ | Angina pectoris, severe | Has chest pain that occurs with minimal physical activity, such as walking only a short distance. After a brief rest, the pain goes away. The person avoids most physical activities because of the pain. | 0.167  (0.110-0.240) |
| Ischemic heart failure | Mild heart failure | Heart failure, mild | The person has shortness of breath and easily tires with moderate physical activity, such as walking uphill or more than a quarter-mile on level ground. The person feels comfortable at rest or during activities requiring less effort. | 0.037  (0.021-0.058) |
|  | Moderate heart failure | Heart failure, moderate | The person has shortness of breath and easily tires with minimal physical activity, such as walking only a short distance. The person feels comfortable at rest but avoids moderate activity. | 0.070  (0.044-0.102) |
|  | Severe heart failure | Heart failure, severe | The person has shortness of breath and feels tired when at rest. The person avoids any physical activity, for fear of worsening the breathing problems. | 0.186  (0.128-0.261) |

**Note:** ^a^ IHD = ischemic heart disease.

^b^ Disability weights based on the Global Burden of Disease study methodology.

**Supplementary TABLE 8** Change in environmental-related IHD Deaths number and DALYs number (thousand) from 1990 to 2021

| Risk  Factor | Measure  Metric | Categories | Global | Sex | | SDI ^b^ region | | | | |
| --- | --- | --- | --- | --- | --- | --- | --- | --- | --- | --- |
|  |  |  |  | Male | Female | High SDI ^b^ | High-middle SDI ^b^ | Middle SDI ^b^ | Low-middle SDI ^b^ | Low SDI ^b^ |
| Environmental | Deaths number | 1990 | 199.49  (159.55,  238.93) | 106.34  (84.52,  127.12) | 93.15  (74.03,  112.00) | 44.85  (32.88,  57.24) | 57.05  (44.10,  70.42) | 38.19  (31.07,  45.19) | 46.91  (38.10,  55.64) | 12.18  (9.66,  14.59) |
|  |  | 2021 | 330.30  (256.25,  397.77) | 180.56  (146.17,  228.37) | 142.24  (109.79,  171.99) | 28.32  (20.76,  35.53) | 75.13  (57.61,  92.47) | 87.25  (69.48,  103.12) | 113.33  (86.54,  138.13) | 25.97  (20.80,  30.91) |
|  |  | %Ch ^c^ | 65.57% | 69.80% | 52.69% | -36.86% | 31.69% | 128.46% | 141.59% | 113.22% |
|  | DALYs number | 1990 | 45866.78  (36754.91,  54702.39) | 27001.40  (21408.95,  32275.33) | 18865.38  (15093.66,  22698.16) | 8351.53  (6190.79,  10664.24) | 12008.20  (9301.27,  14791.64) | 11839.58  (9522.97,  14112.07) | 10350.22  (8362.26,  12270.96) | 3248.98  (2574.99,  3932.52) |
|  |  | 2021 | 70760.63  (55427.07,  84336.70) | 43590.84  (34383.05,  52678.49) | 27169.80  (21160.92,  32456.17) | 4 872.47  (3648.10,  6050.52) | 13506.28  (10436.88,  16811.60) | 24 121.14  (18743.43,  29237.18) | 21630.20  (17269.29,  25539.14) | 6572.29  (5336.49,  7876.62) |
|  |  | %Ch ^c^ | 54.27% | 61.44% | 43.91% | -41.66% | 12.48% | 103.73% | 108.98% | 102.29% |
| Particulate matter  pollution | Deaths number | 1990 | 1570.39  (1199.84,  1966.48) | 829.34  (639.92,  1030.73) | 741.05  (560.91,  935.40) | 305.19  (189.49,  426.05) | 453.10  (326.01,  598.02) | 387.17  (303.94,  473.04) | 318.48  (250.90,  389.01) | 103.86  (81.72,  128.27) |
|  |  | 2021 | 2492.81  (1866.68,  3103.23) | 1412.83  (1044.81,  1782.24) | 1079.98  (799.64,  1348.94) | 156.00  (103.89,  210.01) | 533.50  (379.82,  690.47) | 880.21  (650.80,  1105.17) | 701.84  (545.25,  857.44) | 219.14  (172.58,  268.36) |
|  |  | %Ch ^c^ | 58.74% | 70.36% | 45.74% | -48.88% | 17.74% | 127.35% | 120.37% | 111.00% |
|  | DALYs number | 1990 | 36568.81  (28680.23,  45510.30) | 21320.19  (16671.02,  26331.00) | 15248.62  (11762.39,  18998.40) | 5729.25  (3599.21,  7922.70) | 9562.75  (6989.60,  12548.83) | 9796.78  (7694.63,  12019.16) | 8652.60  (6818.53,  10530.90) | 2771.71  (2200.85,  3418.98) |

**Supplementary TABLE 8. (Continued)**

| Risk  Factor | Measure  Metric | Categories | Global | Sex | | SDI ^b^ region | | | | |
| --- | --- | --- | --- | --- | --- | --- | --- | --- | --- | --- |
|  |  |  |  | Male | Female | High SDI ^b^ | High-middle SDI ^b^ | Middle SDI ^b^ | Low-middle SDI ^b^ | Low SDI ^b^ |
| Particulate matter  pollution | Deaths number | 2021 | 54675.67  (41652.49,  67418.89) | 33494.56  (25184.02,  42071.36) | 21181.12  (15976.28,  26280.63) | 2793.44  (1930.67,  3743.84) | 9716.33  (6922.05,  12705.93) | 18993.28  (14250.25,  23879.75) | 17544.45  (13694.62,  21400.92) | 5583.83  (4389.66,  6850.51) |
|  |  | %Ch ^c^ | 49.51% | 57.10% | 38.91% | -51.24% | 1.61% | 93.87% | 102.76% | 101.46% |
| Non-optimal  temperature | Deaths number | 1990 | 355.69  (291.44,  465.06) | 185.68  (150.37,  244.73) | 170.01  (137.75,  220.71) | 118.03  (100.41,  154.88) | 111.04  (96.12,  141.14) | 65.83  (48.10,  88.91) | 47.96  (28.87,  68.69) | 12.35  (7.84,  17.92) |
|  |  | 2021 | 610.52  (459.42,  862.75) | 339.80  (255.30,  473.53) | 270.73  (203.91,  380.65) | 99.83  (79.89,  136.20) | 174.25  (140.20,  241.84) | 184.32  (137.99,  251.71) | 124.31  (74.28,  186.37) | 27.32  (17.63,  38.68) |
|  |  | %Ch ^c^ | 71.64% | 83.00% | 59.24% | -15.42% | 56.92% | 179.99% | 159.19% | 121.25% |
|  | DALYs number | 1990 | 7720.58  (6227.79,  10156.65) | 4508.11  (3596.47,  5973.28) | 3212.47  (2588.80,  4240.02) | 2151.26  (1862.97,  2817.80) | 2286.65  (1972.50,  2908.87) | 1641.97  (1189.92,  2220.21) | 1295.94  (778.24,  1863.20) | 335.10  (211.93,  484.42) |
|  |  | 2021 | 12418.52  (9158.61,  17640.35) | 7589.53  (5526.06,  10668.89) | 4828.98  (3550.23,  6840.55) | 1683.16  (1372.51,  2319.10) | 2286.65  (1972.50,  2908.87) | 3827.65  (2845.28,  5241.03) | 3141.20  (1853.50,  4707.17) | 698.30  (451.66,  995.45) |
|  |  | %Ch ^c^ | 60.85% | 68.35% | 50.32% | -21.76% | 33.79% | 133.12% | 142.39% | 108.39% |
| Lead  exposure | Deaths number | 1990 | 277.68  (-40.56,  595.67) | 166.57  (-24.42,  357.77) | 111.12  (-16.13,  235.75) | 60.04  (-8.49,  131.07) | 58.20  (-8.19,  126.16) | 69.71  (-9.96,  149.89) | 67.43  (-10.27,  143.82) | 21.91  (-3.35,  45.75) |
|  |  | 2021 | 590.37  (-83.78,  1233.63) | 360.29  (-51.33,  750.59) | 230.08  (-32.45,  491.87) | 49.29  (-6.96,  107.58) | 118.37  (-16.28,  251.98) | 204.07  (-28.95,  424.99) | 169.23  (-24.37,  361.48) | 48.88  (-7.14,  102.01) |

**Supplementary TABLE 8. (Continued)**

| Risk  Factor | Measure  Metric | Categories | Global | Sex | | SDI ^b^ region | | | | |
| --- | --- | --- | --- | --- | --- | --- | --- | --- | --- | --- |
|  |  |  |  | Male | Female | High SDI ^b^ | High-middle SDI ^b^ | Middle SDI ^b^ | Low-middle SDI ^b^ | Low SDI ^b^ |
|  |  | %Ch ^c^ | 112.61% | 116.30% | 107.06% | -17.91% | 103.39% | 192.74% | 150.97% | 123.10% |
|  | DALYs number | 1990 | 6484.40  (-947.86,  14078.20) | 4187.33  (-613.97,  9090.86) | 2297.07  (-333.90,  4867.45) | 1123.44  (-158.08,  2423.69) | 1260.68  (-177.01,  2741.40) | 1735.49  (-247.54,  3755.52) | 1785.42  (-270.99,  3878.57) | 570.73  (-85.91,  1206.64) |
|  |  | 2021 | 11854.61  (-1668.55,  24791.28) | 7698.64  (-1087.98,  16151.34) | 4155.97  (-580.57,  8783.64) | 787.21  (-111.11,  1688.16) | 2027.58  (-275.34,  4329.97) | 4012.49  (-563.06,  8398.08) | 3874.16  (-552.19,  8296.86) | 1143.29  (-165.43,  2416.51) |
|  |  | %Ch ^c^ | 82.82% | 83.86% | 80.93% | -29.93% | 60.84% | 131.20% | 117.00% | 100.32% |

**Note:** Statistical significance was determined using two-sided t-tests, with *P*<0.05 considered significant.

^a^ IHD = ischemic heart disease.

^b^ SDI = Socio-demographic Index, categorized as High (>0.81), High-middle (0.70-0.81), Middle (0.61-0.69), Low-middle (0.46-0.60), and Low (<0.46).

^c^ %Ch = Percent change, represents the relative change in rates from 1990 to 2021. Negative values indicate a decrease in rates; positive values indicate an increase.

**Supplementary TABLE 9** Net and local drifts for IHD ^a^ deaths and DALYs rates attributed to overall environmental factors (%).

|  | Global | | High SDI ^c^ | | High-middle SDI ^c^ | | Middle SDI ^c^ | | Low-middle SDI ^c^ | | Low SDI ^c^ | |
| --- | --- | --- | --- | --- | --- | --- | --- | --- | --- | --- | --- | --- |
|  | Deaths rates | DALYs rates | Deaths rates | DALYs rates | Deaths rates | DALYs rates | Deaths rates | DALYs rates | Deaths rates | DALYs rates | Deaths rates | DALYs rates |
| Sex Net drifts d (95% CI) ^f^ | | | | | | | | | | | | |
| Both | -3.098  (-3.17,  -3.026) | -3.087  (-3.133,  -3.042) | -5.013  (-5.287,  -4.739) | -4.991  (-4.04,  -3.82) | -4.439  (-4.706,  -4.171) | -4.41  (-4.543,  -4.276) | -2.809  (-2.911,  -2.708) | -2.802  (-2.864,  -2.74) | -2.274  (-2.364,  -2.184) | -2.269  (-2.338,  -2.2) | -2.366  (-2.498,  -2.234) | -2.347  (-2.446,  -2.249) |
| Male | -2.933  (-3.04,  -2.825) | -2.924  (-2.994,  -2.854) | -5.019  (-5.301,  -4.737) | -5.005  (-5.167,  -4.843) | -4.269  (-4.545,  -3.993) | -4.252  (-4.395,  -4.109) | -2.521  (-2.643,  -2.398) | -2.517  (-2.592,  -2.441) | -1.922  (-2.032,  -1.812) | -1.92  (-2.01,  -1.83) | -2.006  (-2.187,  -1.825) | -1.991  (-2.126,  -1.856) |
| Female | -3.334  (-3.428,  -3.24) | -3.325  (-3.383,  -3.267) | -5.107  (-5.432,  -4.78) | -5.065  (-5.254,  -4.876) | -4.788  (-5.149,  -4.426) | -4.733  (-4.913,  -4.553) | -3.261  (-3.421,  -3.099) | -3.252  (-3.349,  -3.154) | -2.721  (-2.826,  -2.616) | -2.716  (-2.794,  -2.638) | -2.824  (-2.936,  -2.712) | -2.802  (-2.891,  -2.714) |
| Age group（Both）Local drifts ^e^ (95% CI) ^f^ | | | | | | | | | | | | |
| 15-19 | -2.778  (-4.218,  -1.317) | -2.769  (-3.588,  -1.943) | -4.012  (-10.004,  2.379) | -3.941  (-7.237,  -0.0528) | -3.954  (-9.788,  2.256) | -3.917  (-6.716,  -1.035) | -3.14  (-5.224,  -1.01) | -3.158  (-4.289,  -2.013) | -2.583  (-4.23,  -0.908) | -2.561  (-3.585,  -1.527) | -3.118  (-5.451,  -0.727) | -3.09  (-4.48,  -1.681) |
| 20-24 | -2.728  (-3.418,  -2.033) | -2.719  (-3.119,  -2.318) | -3.168  (-5.836,  -0.424) | -3.113  (-4.598,  -1.605) | -3.879  (-6.515,  -1.168) | -3.836  (-5.112,  -2.542) | -3.002  (-3.978,  -2.016) | -3.011  (-3.55,  -2.47) | -2.733  (-3.534,  -1.925) | -2.712  (-3.219,  -2.202) | -3.074  (-4.271,  -1.861) | -3.035  (-3.761,  -2.304) |
| 25-29 | -2.794  (-3.096,  -2.491) | -2.774  (-2.957,  -2.591) | -2.813  (-4.002,  -1.611) | -2.747  (-3.429,  -2.059) | -4.184  (-5.293,  -3.063) | -4.128  (-4.683,  -3.57) | -2.954  (-3.356,  -2.55) | -2.949  (-3.181,  -2.717) | -2.9  (-3.267,  -2.532) | -2.869  (-3.112,  -2.626) | -2.893  (-3.425,  -2.358) | -2.847  (-3.184,  -2.508) |
| 30-34 | -2.835  (-3.012,  -2.658) | -2.812  (-2.923,  -2.701) | -2.86  (-3.51,  -2.205) | -2.798  (-3.184,  -2.41) | -4.442  (-5.019,  -3.862) | -4.383  (-4.682,  -4.083) | -3.017  (-3.248,  -2.784) | -3.001  (-3.139,  -2.862) | -2.807  (-3.031,  -2.583) | -2.779  (-2.932,  -2.625) | -2.761  (-3.09,  -2.43) | -2.7  (-2.918,  -2.483) |
| 35-39 | -2.847  (-2.974,  -2.721) | -2.823  (-2.905,  -2.74) | -3.355  (-3.778,  -2.929) | -3.297  (-3.558,  -3.034) | -4.778  (-5.168,  -4.387) | -4.718  (-4.927,  -4.508) | -2.995  (-3.161,  -2.829) | -2.974  (-3.078,  -2.871) | -2.635  (-2.799,  -2.471) | -2.608  (-2.726,  -2.491) | -2.64  (-2.882,  -2.397) | -2.575  (-2.741,  -2.408) |
| 40-44 | -2.874  (-2.971,  -2.778) | -2.857  (-2.923,  -2.791) | -3.931  (-4.231,  -3.63) | -3.887  (-4.08,  -3.692) | -4.99  (-5.274,  -4.705) | -4.944  (-5.103,  -4.784) | -3  (-3.128,  -2.872) | -2.988  (-3.071,  -2.904) | -2.459  (-2.587,  -2.331) | -2.433  (-2.53,  -2.337) | -2.573  (-2.76,  -2.386) | -2.512  (-2.647,  -2.377) |

**Supplementary TABLE 9. (Continued)**

|  | Global | | High SDI ^c^ | | High-middle SDI ^c^ | | Middle SDI ^c^ | | Low-middle SDI ^c^ | | Low SDI ^c^ | |
| --- | --- | --- | --- | --- | --- | --- | --- | --- | --- | --- | --- | --- |
|  | Deaths rates | DALYs rates | Deaths rates | DALYs rates | Deaths rates | DALYs rates | Deaths rates | DALYs rates | Deaths rates | DALYs rates | Deaths rates | DALYs rates |
| 45-49 | -2.98  (-3.056,  -2.903) | -2.963  (-3.019,  -2.908) | -4.442  (-4.665,  -4.218) | -4.41  (-4.562,  -4.257) | -4.959  (-5.172,  -4.747) | -4.91  (-5.037,  -4.783) | -3.041  (-3.142,  -2.939) | -3.033  (-3.103,  -2.962) | -2.377  (-2.481,  -2.273) | -2.355  (-2.438,  -2.272) | -2.645  (-2.795,  -2.495) | -2.589  (-2.703,  -2.476) |
| 50-54 | -3.133  (-3.195,  -3.071) | -3.118  (-3.166,  -3.07) | -4.906  (-5.078,  -4.733) | -4.876  (-5.001,  -4.751) | -4.994  (-5.158,  -4.83) | -4.939  (-5.043,  -4.835) | -3.065  (-3.148,  -2.982) | -3.056  (-3.117,  -2.994) | -2.307  (-2.394,  -2.22) | -2.293  (-2.366,  -2.219) | -2.76  (-2.884,  -2.636) | -2.713  (-2.813,  -2.612) |
| 55-59 | -3.241  (-3.293,  -3.189) | -3.229  (-3.272,  -3.186) | -5.341  (-5.477,  -5.204) | -5.303  (-5.409,  -5.196) | -4.828  (-4.96,  -4.696) | -4.774  (-4.863,  -4.684) | -3.097  (-3.167,  -3.026) | -3.09  (-3.146,  -3.034) | -2.273  (-2.347,  -2.198) | -2.264  (-2.332,  -2.195) | -2.798  (-2.904,  -2.692) | -2.761  (-2.853,  -2.67) |
| 60-64 | -3.321  (-3.366,  -3.276) | -3.309  (-3.349,  -3.268) | -5.807  (-5.918,  -5.695) | -5.764  (-5.857,  -5.67) | -4.684  (-4.793,  -4.574) | -4.633  (-4.713,  -4.553) | -3.04  (-3.103,  -2.978) | -3.037  (-3.09,  -2.983) | -2.279  (-2.346,  -2.211) | -2.272  (-2.338,  -2.205) | -2.682  (-2.776,  -2.587) | -2.654  (-2.742,  -2.566) |
| 65-69 | -3.342  (-3.383,  -3.301) | -3.33  (-3.37,  -3.291) | -6.193  (-6.287,  -6.098) | -6.159  (-6.245,  -6.073) | -4.505  (-4.598,  -4.412) | -4.468  (-4.543,  -4.393) | -2.945  (-3.002,  -2.888) | -2.933  (-2.986,  -2.88) | -2.273  (-2.337,  -2.209) | -2.271  (-2.34,  -2.201) | -2.465  (-2.553,  -2.377) | -2.445  (-2.535,  -2.355) |
| 70-74 | -3.25  (-3.288,  -3.211) | -3.239  (-3.281,  -3.198) | -6.445  (-6.527,  -6.362) | -6.436  (-6.519,  -6.353) | -4.219  (-4.304,  -4.133) | -4.19  (-4.266,  -4.113) | -2.72  (-2.775,  -2.666) | -2.702  (-2.759,  -2.646) | -2.185  (-2.25,  -2.12) | -2.195  (-2.272,  -2.117) | -2.107  (-2.196,  -2.018) | -2.103  (-2.204,  -2.003) |
| 75-79 | -3.164  (-3.203,  -3.125) | -3.158  (-3.204,  -3.112) | -6.502  (-6.578,  -6.427) | -6.51  (-6.594,  -6.427) | -3.995  (-4.077,  -3.914) | -3.998  (-4.078,  -3.917) | -2.421  (-2.477,  -2.365) | -2.408  (-2.473,  -2.344) | -2.01  (-2.08,  -1.939) | -2.032  (-2.125,  -1.938) | -1.726  (-1.825,  -1.627) | -1.756  (-1.88,  -1.631) |
| 80-84 | -3.083  (-3.123,  -3.042) | -3.086  (-3.14,  -3.032) | -6.309  (-6.38,  -6.239) | -6.346  (-6.432,  -6.259) | -3.662  (-3.743,  -3.581) | -3.699  (-3.788,  -3.609) | -2.106  (-2.168,  -2.043) | -2.105  (-2.185,  -2.024) | -1.742  (-1.826,  -1.657) | -1.772  (-1.896,  -1.648) | -1.427  (-1.55,  -1.304) | -1.488  (-1.66,  -1.316) |
| 85-89 | -3.109  (-3.158,  -3.06) | -3.116  (-3.188,  -3.044) | -5.938  (-6.012,  -5.864) | -5.995  (-6.094,  -5.895) | -3.582  (-3.678,  -3.487) | -3.635  (-3.751,  -3.519) | -1.906  (-1.986,  -1.827) | -1.905  (-2.018,  -1.792) | -1.536  (-1.65,  -1.421) | -1.571  (-1.756,  -1.386) | -1.204  (-1.382,  -1.026) | -1.28  (-1.555,  -1.003) |
| 90-94 | -3.28  (-3.352,  -3.208) | -3.288  (-3.402,  -3.174) | -5.63  (-5.723,  -5.537) | -5.691  (-5.826,  -5.556) | -3.589  (-3.731,  -3.446) | -3.6  (-3.837,  -3.463) | -1.955  (-2.08,  -1.829) | -1.955  (-2.147,  -1.763) | -1.51  (-1.694,  -1.325) | -1.545  (-1.866,  -1.222) | -1.058  (-1.365,  -0.75) | -1.133  (-1.646,  -0.618) |

**Supplementary TABLE 9. (Continued)**

|  | Global | | High SDI ^c^ | | High-middle SDI ^c^ | | Middle SDI ^c^ | | Low-middle SDI ^c^ | | Low SDI ^c^ | |
| --- | --- | --- | --- | --- | --- | --- | --- | --- | --- | --- | --- | --- |
|  | Deaths rates | DALYs rates | Deaths rates | DALYs rates | Deaths rates | DALYs rates | Deaths rates | DALYs rates | Deaths rates | DALYs rates | Deaths rates | DALYs rates |
| 95+ | -3.678  (-3.813,  -3.543) | -3.697  (-3.92,  -3.473) | -5.499  (-5.648,  -5.35) | -5.571  (-5.801,  -5.341) | -3.983  (-4.263,  -3.701) | -4.06  (-4.446,  -3.673) | -2.234  (-2.492,  -1.976) | -2.248  (-2.656,  -1.838) | -1.641  (-2.015,  -1.266) | -1.681  (-2.357,  -1) | -0.949  (-1.595,  -0.298) | -1.026  (-2.14,  0.102) |
| Age group（male）Local drifts ^e^ (95% CI) ^f^ | | | | | | | | | | | | |
| 15-19 | -2.778  (-4.218,  -1.317) | -2.58  (-3.855,  -1.289) | -3.91  (-10.144,  2.756) | -3.821  (-7.263,  -0.252) | -3.8  (-9.886,  2.697) | -3.765  (-6.745,  -0.69) | -2.929  (-5.479,  -0.039) | -2.916  (-4.28,  -1.532) | -2.075  (-4.178,  0.074) | -2.035  (-3.412,  -0.638) | -2.512  (-5.877,  0.973) | -2.46  (-4.455,  -0.424) |
| 20-24 | -2.728  (-3.418,  -2.033) | -2.474  (-3.077,  -1.867) | -3.114  (-5.865,  -0.282) | -3.042  (-4.579,  -1.48) | -3.656  (-6.368,  -0.865) | -3.618  (-4.958,  -2.258) | -2.722  (-3.897,  -1.533) | -2.708  (-3.346,  -2.065) | -2.262  (-3.247,  -1.268) | -2.223  (-2.881,  -1.562) | -2.466  (-4.146,  -0.755) | -2.403  (-3.416,  -1.38) |
| 25-29 | -2.794  (-3.096,  -2.491) | -2.512  (-2.774,  -2.25) | -2.798  (-3.989,  -1.593) | -2.716  (-3.403,  -2.024) | -3.95  (-5.058,  -2.83) | -3.898  (-4.465,  -3.328) | -2.639  (-3.104,  -2.171) | -2.614  (-2.878,  -2.349) | -2.464  (-2.891,  -2.036) | -2.418  (-2.716,  -2.119) | -2.362  (-3.051,  -1.667) | -2.294  (-2.73,  -1.857) |
| 30-34 | -2.835  (-3.012,  -2.658) | -2.601  (-2.756,  -2.445) | -2.909  (-3.55,  -2.263) | -2.834  (-3.217,  -2.45) | -4.266  (-4.837,  -3.691) | -4.212  (-4.514,  -3.908) | -2.704  (-2.967,  -2.439) | -2.672  (-2.828,  -2.517) | -2.441  (-2.695,  -2.186) | -2.395  (-2.579,  -2.21) | -2.359  (-2.776,  -1.94) | -2.278  (-2.551,  -2.003) |
| 35-39 | -2.847  (-2.974,  -2.721) | -2.683  (-2.797,  -2.569) | -3.459  (-3.872,  -3.044) | -3.393  (-3.649,  -3.136) | -4.656  (-5.04,  -4.271) | -4.601  (-4.813,  -4.389) | -2.724  (-2.911,  -2.537) | -2.691  (-2.805,  -2.575) | -2.33  (-2.514,  -2.146) | -2.29  (-2.429,  -2.151) | -2.301  (-2.6,  -2.001) | -2.217  (-2.422,  -2.012) |
| 40-44 | -2.874  (-2.971,  -2.778) | -2.757  (-2.848,  -2.666) | -4.063  (-4.353,  -3.772) | -4.015  (-4.204,  -3.826) | -4.905  (-5.185,  -4.626) | -4.863  (-5.024,  -4.702) | -2.757  (-2.901,  -2.613) | -2.733  (-2.825,  -2.64) | -2.164  (-2.307,  -2.022) | -2.129  (-2.242,  -2.016) | -2.253  (-2.48,  -2.025) | -2.179  (-2.342,  -2.015) |
| 45-49 | -2.98  (-3.056,  -2.903) | -2.875  (-2.951,  -2.799) | -4.563  (-4.779,  -4.346) | -4.532  (-4.68,  -4.383) | -4.883  (-5.093,  -4.673) | -4.841  (-4.969,  -4.712) | -2.8  (-2.915,  -2.684) | -2.781  (-2.859,  -2.702) | -2.108  (-2.224,  -1.992) | -2.076  (-2.173,  -1.979) | -2.362  (-2.543,  -2.181) | -2.297  (-2.434,  -2.159) |
| 50-54 | -3.133  (-3.195,  -3.071) | -3.021  (-3.087,  -2.954) | -4.987  (-5.155,  -4.82) | -4.966  (-5.088,  -4.844) | -4.928  (-5.093,  -4.764) | -4.885  (-4.992,  -4.778) | -2.793  (-2.888,  -2.698) | -2.779  (-2.848,  -2.709) | -2.04  (-2.138,  -1.943) | -2.022  (-2.109,  -1.935) | -2.503  (-2.654,  -2.351) | -2.451  (-2.572,  -2.329) |
| 55-59 | -3.241  (-3.293,  -3.189) | -3.129  (-3.19,  -3.068) | -5.367  (-5.501,  -5.232) | -5.341  (-5.446,  -5.236) | -4.748  (-4.883,  -4.613) | -4.702  (-4.796,  -4.607) | -2.82  (-2.903,  -2.738) | -2.807  (-2.872,  -2.743) | -2.017  (-2.102,  -1.932) | -2.007  (-2.089,  -1.926) | -2.575  (-2.705,  -2.444) | -2.534  (-2.647,  -2.422) |

**Supplementary TABLE 9. (Continued)**

|  | Global | | High SDI ^c^ | | High-middle SDI ^c^ | | Middle SDI ^c^ | | Low-middle SDI ^c^ | | Low SDI ^c^ | |
| --- | --- | --- | --- | --- | --- | --- | --- | --- | --- | --- | --- | --- |
|  | Deaths rates | DALYs rates | Deaths rates | DALYs rates | Deaths rates | DALYs rates | Deaths rates | DALYs rates | Deaths rates | DALYs rates | Deaths rates | DALYs rates |
| 60-64 | -3.321  (-3.366,  -3.276) | -3.2  (-3.258,  -3.142) | -5.783  (-5.894,  -5.671) | -5.755  (-5.85,  -5.66) | -4.583  (-4.698,  -4.467) | -4.545  (-4.632,  -4.458) | -2.764  (-2.839,  -2.69) | -2.758  (-2.821,  -2.696) | -1.984  (-2.062,  -1.906) | -1.984  (-2.065,  -1.903) | -2.435  (-2.552,  -2.318) | -2.41  (-2.519,  -2.301) |
| 65-69 | -3.342  (-3.383,  -3.301) | -3.24  (-3.299,  -3.181) | -6.147  (-6.244,  -6.049) | -6.133  (-6.223,  -6.043) | -4.396  (-4.498,  -4.292) | -4.378  (-4.463,  -4.293) | -2.679  (-2.749,  -2.61) | -2.673  (-2.737,  -2.609) | -1.981  (-2.057,  -1.905) | -1.991  (-2.077,  -1.905) | -2.227  (-2.339,  -2.116) | -2.216  (-2.33,  -2.102) |
| 70-74 | -3.25  (-3.288,  -3.211) | -3.169  (-3.234,  -3.105) | -6.431  (-6.52,  -6.343) | -6.443  (-6.533,  -6.353) | -4.089  (-4.189,  -3.988) | -4.076  (-4.168,  -3.984) | -2.462  (-2.531,  -2.393) | -2.462  (-2.533,  -2.392) | -1.881  (-1.96,  -1.802) | -1.908  (-2.007,  -1.809) | -1.849  (-1.964,  -1.733) | -1.864  (-1.994,  -1.734) |
| 75-79 | -3.164  (-3.203,  -3.125) | -3.049  (-3.124,  -2.974) | -6.482  (-6.567,  -6.396) | -6.514  (-6.609,  -6.418) | -3.814  (-3.917,  -3.712) | -3.833  (-3.938,  -3.729) | -2.136  (-2.209,  -2.062) | -2.156  (-2.24,  -2.072) | -1.652  (-1.74,  -1.564) | -1.693  (-1.817,  -1.57) | -1.415  (-1.547,  -1.284) | -1.477  (-1.642,  -1.311) |
| 80-84 | -3.083  (-3.123,  -3.042) | -2.871  (-2.963,  -2.778) | -6.253  (-6.339,  -6.168) | -6.316  (-6.422,  -6.211) | -3.353  (-3.463,  -3.244) | -3.415  (-3.539,  -3.291) | -1.727  (-1.813,  -1.641) | -1.776  (-1.885,  -1.666) | -1.335  (-1.442,  -1.228) | -1.389  (-1.555,  -1.222) | -1.006  (-1.173,  -0.838) | -1.107  (-1.339,  -0.873) |
| 85-89 | -3.109  (-3.158,  -3.06) | -2.727  (-2.859,  -2.595) | -5.817  (-5.915,  -5.719) | -5.898  (-6.032,  -5.764) | -3.12  (-3.26,  -2.98) | -3.199  (-3.373,  -3.024) | -1.388  (-1.501,  -1.274) | -1.435  (-1.593,  -1.276) | -1.023  (-1.171,  -0.876) | -1.087  (-1.339,  -0.834) | -0.634  (-0.883,  -0.384) | -0.754  (-1.137,  -0.369) |
| 90-94 | -3.28  (-3.352,  -3.208) | -2.768  (-2.995,  -2.541) | -5.46  (-5.598,  -5.321) | -5.541  (-5.746,  -5.336) | -3.015  (-3.244,  -2.785) | -3.09  (-3.4,  -2.78) | -1.438  (-1.625,  -1.251) | -1.476  (-1.757,  -1.195) | -0.891  (-1.134,  -0.649) | -0.954  (-1.398,  -0.508) | -0.384  (-0.83,  0.064) | -0.502  (-1.241,  0.243) |
| 95+ | -3.678  (-3.813,  -3.543) | -3.232  (-3.725,  -2.736) | -5.369  (-5.624,  -5.113) | -5.46  (-5.861,  -5.057) | -3.562  (-4.07,  -3.052) | -3.65  (-4.364,  -2.93) | -2.095  (-2.508,  -1.681) | -2.142  (-2.786,  -1.494) | -0.965  (-1.472,  -0.456) | -1.029  (-1.993,  -0.056) | -0.213  (-1.216,  0.8) | -0.326  (-2.04,  1.417) |
| Age group（female）Local drifts ^e^ (95% CI) ^f^ | | | | | | | | | | | | |
| 15-19 | -3.064  (-4.808,  -1.289) | -3.079  (-4.051,  -2.098) | -4.319  (-11.1,  2.978) | -4.263  (-8.049,  -0.32) | -4.519  (-11.998,  3.597) | -4.461  (-8.074,  -0.707) | -3.564  (-6.693,  -0.029) | -3.619  (-5.335,  -1.873) | -3.22  (-5.006,  -1.401) | -3.221  (-4.307,  -2.123) | -3.688  (-5.527,  -1.812) | -3.684  (-4.848,  -2.505) |
| 20-24 | -3.141  (-4.016,  -2.259) | -3.153  (-3.648,  -2.655) | -3.419  (-6.513,  -0.223) | -3.378  (-5.124,  -1.601) | -4.669  (-8.19,  -1.013) | -4.596  (-6.306,  -2.854) | -3.586  (-5.102,  -2.046) | -3.618  (-4.461,  -2.767) | -3.416  (-4.325,  -2.498) | -3.417  (-3.98,  -2.85) | -3.731  (-4.703,  -2.75) | -3.715  (-4.34,  -3.085) |

**Supplementary TABLE 9. (Continued)**

|  | Global | | High SDI ^c^ | | High-middle SDI ^c^ | | Middle SDI ^c^ | | Low-middle SDI ^c^ | | Low SDI ^c^ | |
| --- | --- | --- | --- | --- | --- | --- | --- | --- | --- | --- | --- | --- |
|  | Deaths rates | DALYs rates | Deaths rates | DALYs rates | Deaths rates | DALYs rates | Deaths rates | DALYs rates | Deaths rates | DALYs rates | Deaths rates | DALYs rates |
| 25-29 | -3.289  (-3.704,  -2.872) | -3.285  (-3.53,  -3.039) | -3.002  (-4.485,  -1.496) | -2.951  (-3.812,  -2.083) | -5.032  (-6.642,  -3.395) | -4.942  (-5.745,  -4.133) | -3.659  (-4.335,  -2.979) | -3.666  (-4.057,  -3.274) | -3.623  (-4.072,  -3.172) | -3.608  (-3.898,  -3.317) | -3.614  (-4.085,  -3.141) | -3.58  (-3.896,  -3.262) |
| 30-34 | -3.242  (-3.496,  -2.986) | -3.237  (-3.393,  -3.081) | -2.893  (-3.746,  -2.033) | -2.847  (-3.358,  -2.333) | -5.1  (-5.962,  -4.231) | -5.004  (-5.448,  -4.558) | -3.723  (-4.128,  -3.317) | -3.714  (-3.956,  -3.471) | -3.436  (-3.72,  -3.151) | -3.428  (-3.619,  -3.238) | -3.374  (-3.675,  -3.072) | -3.329  (-3.54,  -3.118) |
| 35-39 | -3.108  (-3.296,  -2.921) | -3.102  (-3.221,  -2.982) | -3.213  (-3.792,  -2.631) | -3.164  (-3.525,  -2.802) | -5.248  (-5.84,  -4.653) | -5.151  (-5.467,  -4.834) | -3.593  (-3.889,  -3.296) | -3.58  (-3.765,  -3.396) | -3.148  (-3.361,  -2.934) | -3.138  (-3.287,  -2.989) | -3.207  (-3.436,  -2.978) | -3.157  (-3.324,  -2.99) |
| 40-44 | -3.038  (-3.183,  -2.892) | -3.04  (-3.137,  -2.943) | -3.662  (-4.082,  -3.241) | -3.616  (-3.89,  -3.341) | -5.33  (-5.766,  -4.893) | -5.254  (-5.497,  -5.01) | -3.492  (-3.721,  -3.262) | -3.499  (-3.648,  -3.349) | -2.927  (-3.096,  -2.758) | -2.918  (-3.042,  -2.793) | -3.131  (-3.313,  -2.949) | -3.083  (-3.222,  -2.944) |
| 45-49 | -3.087  (-3.203,  -2.972) | -3.095  (-3.176,  -3.014) | -4.171  (-4.485,  -3.856) | -4.124  (-4.34,  -3.908) | -5.193  (-5.513,  -4.871) | -5.112  (-5.302,  -4.923) | -3.468  (-3.647,  -3.289) | -3.485  (-3.608,  -3.361) | -2.772  (-2.909,  -2.634) | -2.772  (-2.879,  -2.666) | -3.135  (-3.281,  -2.988) | -3.092  (-3.209,  -2.974) |
| 50-54 | -3.269  (-3.362,  -3.176) | -3.268  (-3.337,  -3.198) | -4.741  (-4.98,  -4.501) | -4.676  (-4.85,  -4.502) | -5.202  (-5.443,  -4.961) | -5.107  (-5.258,  -4.956) | -3.518  (-3.662,  -3.374) | -3.526  (-3.631,  -3.42) | -2.703  (-2.817,  -2.589) | -2.703  (-2.797,  -2.609) | -3.183  (-3.304,  -3.062) | -3.145  (-3.248,  -3.042) |
| 55-59 | -3.383  (-3.458,  -3.308) | -3.385  (-3.445,  -3.324) | -5.356  (-5.54,  -5.172) | -5.273  (-5.416,  -5.13) | -5.067  (-5.251,  -4.882) | -4.978  (-5.102,  -4.854) | -3.527  (-3.644,  -3.409) | -3.538  (-3.631,  -3.445) | -2.642  (-2.738,  -2.546) | -2.645  (-2.729,  -2.56) | -3.123  (-3.224,  -3.022) | -3.098  (-3.191,  -3.006) |
| 60-64 | -3.514  (-3.578,  -3.451) | -3.505  (-3.56,  -3.45) | -6.009  (-6.153,  -5.865) | -5.916  (-6.036,  -5.796) | -4.969  (-5.114,  -4.825) | -4.888  (-4.992,  -4.784) | -3.468  (-3.568,  -3.367) | -3.474  (-3.56,  -3.388) | -2.692  (-2.777,  -2.608) | -2.682  (-2.763,  -2.601) | -3.002  (-3.09,  -2.913) | -2.974  (-3.061,  -2.886) |
| 65-69 | -3.536  (-3.59,  -3.482) | -3.518  (-3.57,  -3.467) | -6.533  (-6.647,  -6.419) | -6.452  (-6.556,  -6.349) | -4.77  (-4.883,  -4.656) | -4.705  (-4.795,  -4.615) | -3.345  (-3.432,  -3.257) | -3.33  (-3.412,  -3.249) | -2.639  (-2.716,  -2.562) | -2.624  (-2.705,  -2.543) | -2.736  (-2.817,  -2.656) | -2.708  (-2.795,  -2.62) |
| 70-74 | -3.432  (-3.481,  -3.383) | -3.406  (-3.457,  -3.355) | -6.801  (-6.894,  -6.708) | -6.752  (-6.844,  -6.659) | -4.477  (-4.572,  -4.381) | -4.426  (-4.51,  -4.342) | -3.087  (-3.167,  -3.006) | -3.049  (-3.132,  -2.966) | -2.521  (-2.595,  -2.446) | -2.505  (-2.592,  -2.418) | -2.389  (-2.467,  -2.31) | -2.364  (-2.459,  -2.269) |

**Supplementary TABLE 9. (Continued)**

|  | Global | | High SDI ^c^ | | High-middle SDI ^c^ | | Middle SDI ^c^ | | Low-middle SDI ^c^ | | Low SDI ^c^ | |
| --- | --- | --- | --- | --- | --- | --- | --- | --- | --- | --- | --- | --- |
|  | Deaths rates | DALYs rates | Deaths rates | DALYs rates | Deaths rates | DALYs rates | Deaths rates | DALYs rates | Deaths rates | DALYs rates | Deaths rates | DALYs rates |
| 75-79 | -3.38  (-3.426,  -3.334) | -3.349  (-3.402,  -3.295) | -6.829  (-6.907,  -6.751) | -6.806  (-6.893,  -6.72) | -4.252  (-4.337,  -4.167) | -4.239  (-4.322,  -4.156) | -2.783  (-2.861,  -2.704) | -2.736  (-2.826,  -2.645) | -2.359  (-2.437,  -2.28) | -2.352  (-2.454,  -2.25) | -2.049  (-2.134,  -1.964) | -2.046  (-2.16,  -1.933) |
| 80-84 | -3.368  (-3.414,  -3.322) | -3.341  (-3.401,  -3.282) | -6.599  (-6.666,  -6.532) | -6.616  (-6.699,  -6.533) | -3.954  (-4.032,  -3.876) | -3.98  (-4.066,  -3.894) | -2.498  (-2.582,  -2.415) | -2.447  (-2.555,  -2.339) | -2.094  (-2.186,  -2.002) | -2.093  (-2.226,  -1.96) | -1.849  (-1.952,  -1.746) | -1.87  (-2.022,  -1.717) |
| 85-89 | -3.481  (-3.533,  -3.428) | -3.452  (-3.527,  -3.377) | -6.185  (-6.25,  -6.121) | -6.23  (-6.319,  -6.141) | -3.907  (-3.995,  -3.819) | -3.952  (-4.059,  -3.845) | -2.347  (-2.448,  -2.245) | -2.29  (-2.435,  -2.144) | -1.961  (-2.084,  -1.838) | -1.96  (-2.155,  -1.765) | -1.765  (-1.91,  -1.62) | -1.798  (-2.036,  -1.559) |
| 90-94 | -3.672  (-3.745,  -3.598) | -3.647  (-3.76,  -3.534) | -5.824  (-5.899,  -5.749) | -5.883  (-5.994,  -5.772) | -3.892  (-4.016,  -3.767) | -3.954  (-4.117,  -3.79) | -2.324  (-2.481,  -2.167) | -2.274  (-2.514,  -2.033) | -2.013  (-2.209,  -1.817) | -2.015  (-2.349,  -1.681) | -1.702  (-1.944,  -1.459) | -1.738  (-2.166,  -1.307) |
| 95+ | -3.976  (-4.107,  -3.845) | -3.96  (-4.171,  -3.749) | -5.609  (-5.723,  -5.495) | -5.685  (-5.861,  -5.508) | -4.164  (-4.398,  -3.929) | -4.241  (-4.562,  -3.918) | -2.322  (-2.628,  -2.016) | -2.289  (-2.776,  -1.799) | -2.152  (-2.537,  -1.766) | -2.163  (-2.845,  -1.475) | -1.6  (-2.085,  -1.113) | -1.645  (-2.534,  -0.748) |

**Note:** ^a^ IHD = ischemic heart disease;

^b^ DALYs = disability adjusted life years.

^c^ SDI = socio-demographic index.

^d^ Net drifts represent the overall annual percentage change in the age-standardized rate based on period and birth cohort.

^e^ Local drifts indicate the annual percentage change over time specific to the age group.

^f^ CI = confidence interval. All net and local drifts were statistically significant (*P*<0.05).

**Supplementary TABLE** **10** Net and local drifts for IHD ^a^ deaths and DALYs ^b^ rates attributed to particulate matter pollution (%).

|  | Global | | High SDI ^c^ | | High-middle SDI ^c^ | | Middle SDI ^c^ | | Low-middle SDI ^c^ | | Low SDI ^c^ | |
| --- | --- | --- | --- | --- | --- | --- | --- | --- | --- | --- | --- | --- |
|  | Deaths rates | DALYs rates | Deaths rates | DALYs rates | Deaths rates | DALYs rates | Deaths rates | DALYs rates | Deaths rates | DALYs rates | Deaths rates | DALYs rates |
| Sex Net drifts ^d^ (95% CI) ^f^ | | | | | | | | | | | | |
| Both | -3.229  (-3.267,  -3.191) | -3.218  (-3.248,  -3.187) | -5.708  (-5.827,  -5.588) | -5.672  (-5.756,  -5.588) | -4.703  (-4.812,  -4.593) | -4.674  (-4.743,  -4.605) | -2.91  (-2.964,  -2.856) | -2.617  (-2.672,  -2.562) | -2.318  (-2.373,  -2.263) | -2.317  (-2.376,  -2.258) | -2.286  (-2.366,  -2.206) | -2.274  (-2.36,  -2.187) |
| Male | -3.051  (-3.104,  -2.999) | -3.047  (-3.095,  -2.998) | -5.019  (-5.301,  -4.737) | -5.694  (-5.781,  -5.608) | -4.516  (-4.632,  -4.399) | -4.5  (-4.583,  -4.418) | -2.619  (-2.681,  -2.556) | -2.517  (-2.592,  -2.441) | -1.969  (-2.034,  -1.904) | -1.974  (-2.052,  -1.896) | -1.935  (-2.041,  -1.829) | -1.931  (-2.052,  -1.81) |
| Female | -3.463  (-3.52,  -3.406) | -3.449  (-3.492,  -3.405) | -5.802  (-5.963,  -5.641) | -5.745  (-5.85,  -5.64) | -5.023  (-5.179,  -4.867) | -4.967  (-5.057,  -4.876) | -3.334  (-3.42,  -3.2425) | -3.316  (-3.388,  -3.245) | -2.742  (-2.811,  -2.674) | -3.316  (-3.388,  -3.245) | -2.727  (-2.798,  -2.655) | -2.802  (-2.891,  -2.714) |
| Age group (Both) Local drifts ^e^ (95% CI) ^f^ | | | | | | | | | | | | |
| 25-29 | -2.843  (-3.199,  -2.486) | -2.833  (-3.038,  -2.627) | -2.768  (-4.327,  -1.184) | -2.675  (-3.545,  -1.796) | -4.364  (-5.719,  -2.989) | -4.301  (-4.977,  -3.619) | -3.006  (-3.5,  -2.508) | -3.014  (-3.29,  -2.736) | -3.016  (-3.442,  -2.589) | -2.993  (-3.271,  -2.715) | -2.87  (-3.465,  -2.272) | -2.83  (-3.209,  -2.45) |
| 30-34 | -2.838  (-3.027,  -2.648) | -2.821  (-2.934,  -2.708) | -2.729  (-3.48,  -1.971) | -2.666  (-3.098,  -2.233) | -4.523  (-5.144,  -3.897) | -4.459  (-4.777,  -4.139) | -3.056  (-3.315,  -2.797) | -3.046  (-3.195,  -2.897) | -2.867  (-3.102,  -2.632) | -2.843  (-3.001,  -2.684) | -2.732  (-3.072,  -2.39) | -2.673  (-2.898,  -2.448) |
| 35-39 | -2.836  (-2.969,  -2.702) | -2.815  (-2.898,  -2.732) | -3.291  (-3.773,  -2.807) | -3.238  (-3.525,  -2.95) | -4.883  (-5.297,  -4.468) | -4.819  (-5.038,  -4.598) | -3.037  (-3.219,  -2.854) | -3.018  (-3.127,  -2.908) | -2.677  (-2.847,  -2.507) | -2.652  (-2.771,  -2.533) | -2.605  (-2.853,  -2.357) | -2.541  (-2.711,  -2.37) |
| 40-44 | -2.866  (-2.968,  -2.764) | -2.852  (-2.918,  -2.786) | -3.987  (-4.33,  -3.643) | -3.949  (-4.163,  -3.734) | -5.128  (-5.43,  -4.826) | -5.079  (-5.247,  -4.911) | -3.059  (-3.2,  -2.918) | -3.048  (-3.137,  -2.96) | -2.503  (-2.635,  -2.37) | -2.478  (-2.576,  -2.381) | -2.534  (-2.726,  -2.342) | -2.474  (-2.612,  -2.336) |
| 45-49 | -2.995  (-3.076,  -2.914) | -2.981  (-3.036,  -2.925) | -4.627  (-4.886,  -4.367) | -4.598  (-4.769,  -4.427) | -5.134  (-5.361,  -4.907) | -5.081  (-5.214,  -4.947) | -3.122  (-3.234,  -3.009) | -3.115  (-3.189,  -3.04) | -2.432  (-2.54,  -2.324) | -2.411  (-2.495,  -2.327) | -2.61  (-2.764,  -2.456) | -2.555  (-2.672,  -2.438) |
| 50-54 | -3.175  (-3.241,  -3.108) | -3.161  (-3.21,  -3.112) | -5.262  (-5.465,  -5.058) | -5.227  (-5.369,  -5.085) | -5.195  (-5.371,  -5.019) | -5.135  (-5.245,  -5.025) | -3.17  (-3.263,  -3.078) | -3.161  (-3.226,  -3.095) | -2.383  (-2.474,  -2.293) | -2.369  (-2.445,  -2.294) | -2.732  (-2.861,  -2.604) | -2.686  (-2.789,  -2.583) |

**Supplementary TABLE 10. (Continued)**

|  | Global | | High SDI ^c^ | | High-middle SDI ^c^ | | Middle SDI ^c^ | | Low-middle SDI ^c^ | | Low SDI ^c^ | |
| --- | --- | --- | --- | --- | --- | --- | --- | --- | --- | --- | --- | --- |
|  | Deaths rates | DALYs rates | Deaths rates | DALYs rates | Deaths rates | DALYs rates | Deaths rates | DALYs rates | Deaths rates | DALYs rates | Deaths rates | DALYs rates |
| 55-59 | -3.319  (-3.375,  -3.263) | -3.307  (-3.351,  -3.263) | -5.855  (-6.019,  -5.691) | -5.8  (-5.923,  -5.678) | -5.089  (-5.231,  -4.947) | -5.028  (-5.124,  -4.933) | -3.228  (-3.307,  -3.149) | -3.221  (-3.281,  -3.16) | -2.371  (-2.449,  -2.292) | -2.362  (-2.431,  -2.292) | -2.782  (-2.892,  -2.672) | -2.746  (-2.841,  -2.651) |
| 60-64 | -3.421  (-3.47,  -3.371) | -3.408  (-3.45,  -3.366) | -6.407  (-6.542,  -6.272) | -6.342  (-6.451,  -6.233) | -4.992  (-5.111,  -4.873) | -4.936  (-5.021,  -4.849) | -3.192  (-3.262,  -3.121) | -3.186  (-3.244,  -3.129) | -2.395  (-2.466,  -2.324) | -2.386  (-2.454,  -2.318) | -2.686  (-2.784,  -2.589) | -2.658  (-2.749,  -2.567) |
| 65-69 | -3.446  (-3.491,  -3.402) | -3.433  (-3.474,  -3.392) | -6.81  (-6.925,  -6.695) | -6.754  (-6.855,  -6.653) | -4.822  (-4.923,  -4.721) | -4.781  (-4.861,  -4.7) | -3.114  (-3.177,  -3.05) | -3.098  (-3.155,  -3.041) | -2.401  (-2.469,  -2.334) | -2.397  (-2.467,  -2.326) | -2.496  (-2.587,  -2.404) | -2.475  (-2.568,  -2.381) |
| 70-74 | -3.361  (-3.403,  -3.319) | -3.346  (-3.389,  -3.303) | -7.052  (-7.154,  -6.951) | -7.024  (-7.121,  -6.926) | -4.56  (-4.652,  -4.467) | -4.526  (-4.607,  -4.444) | -2.912  (-2.973,  -2.85) | -2.888  (-2.95,  -2.827) | -2.325  (-2.393,  -2.257) | -2.331  (-2.41,  -2.252) | -2.167  (-2.259,  -2.074) | -2.161  (-2.266,  -2.057) |
| 75-79 | -3.28  (-3.323,  -3.238) | -3.268  (-3.316,  -3.22) | -7.102  (-7.194,  -7.009) | -7.093  (-7.191,  -6.995) | -4.35  (-4.438,  -4.261) | -4.347  (-4.433,  -4.261) | -2.64  (-2.703,  -2.577) | -2.621  (-2.691,  -2.552) | -2.158  (-2.232,  -2.083) | -2.176  (-2.272,  -2.08) | -1.807  (-1.91,  -1.704) | -1.836  (-1.965,  -1.706) |
| 80-84 | -3.216  (-3.261,  -3.171) | -3.211  (-3.268,  -3.155) | -6.919  (-7.005,  -6.832) | -6.943  (-7.044,  -6.841) | -4.037  (-4.125,  -3.95) | -4.068  (-4.163,  -3.973) | -2.354  (-2.424,  -2.284) | -2.345  (-2.432,  -2.258) | -1.897  (-1.986,  -1.809) | -1.924  (-2.052,  -1.797) | -1.52  (-1.647,  -1.392) | -1.58  (-1.758,  -1.402) |
| 85-89 | -3.264  (-3.318,  -3.209) | -3.262  (-3.337,  -3.187) | -6.592  (-6.682,  -6.501) | -6.638  (-6.754,  -6.521) | -3.955  (-4.058,  -3.852) | -4.004  (-4.126,  -3.881) | -2.177  (-2.265,  -2.088) | -2.168  (-2.288,  -2.047) | -1.701  (-1.821,  -1.581) | -1.733  (-1.923,  -1.543) | -1.304  (-1.489,  -1.119) | -1.379  (-1.664,  -1.093) |
| 90-94 | -3.46  (-3.541,  -3.38) | -3.459  (-3.579,  -3.338) | -6.365  (-6.478,  -6.253) | -6.415  (-6.571,  -6.258) | -3.968  (-4.12,  -3.815) | -4.024  (-4.221,  -3.827) | -2.222  (-2.362,  -2.082) | -2.215  (-2.42,  -2.009) | -1.69  (-1.883,  -1.497) | -1.722  (-2.051,  -1.392) | -1.169  (-1.488,  -0.851) | -1.244  (-1.772,  -0.713) |
| 95+ | -3.888  (-4.04,  -3.735) | -3.897  (-4.135,  -3.657) | -6.31  (-6.489,  -6.13) | -6.369  (-6.635,  -6.102) | -4.379  (-4.679,  -4.078) | -4.452  (-4.858,  -4.044) | -2.494  (-2.781,  -2.207) | -2.499  (-2.936,  -2.06) | -1.841  (-2.231,  -1.449) | -1.876  (-2.567,  -1.18) | -1.073  (-1.74,  -0.401) | -1.148  (-2.294,  0.012) |
| Age group (male) Local drifts ^e^ (95% CI) ^f^ | | | | | | | | | | | | |
| 25-29 | -2.571  (-3.047,  -2.092) | -2.538  (-2.823,  -2.252) | -2.716  (-4.222,  -1.186) | -2.608  (-3.459,  -1.749) | -4.069  (-5.436,  -2.681) | -4.069  (-5.436,  -2.681) | -2.682  (-3.225,  -2.137) | -2.665  (-2.963,  -2.366) | -2.557  (-3.043,  -2.068) | -2.517  (-2.856,  -2.177) | -2.333  (-3.095,  -1.566) | -2.27  (-2.753,  -1.786) |

**Supplementary TABLE 10. (Continued)**

|  | Global | | High SDI ^c^ | | High-middle SDI ^c^ | | Middle SDI ^c^ | | Low-middle SDI ^c^ | | Low SDI ^c^ | |
| --- | --- | --- | --- | --- | --- | --- | --- | --- | --- | --- | --- | --- |
|  | Deaths rates | DALYs rates | Deaths rates | DALYs rates | Deaths rates | DALYs rates | Deaths rates | DALYs rates | Deaths rates | DALYs rates | Deaths rates | DALYs rates |
| 30-34 | -2.625  (-2.875,  -2.374) | -2.588  (-2.742,  -2.433) | -2.752  (-3.471,  -2.027) | -2.679  (-3.096,  -2.259) | -4.315  (-4.944,  -3.683) | -4.315  (-4.944,  -3.683) | -2.732  (-3.013,  -2.451) | -2.706  (-2.865,  -2.546) | -2.483  (-2.749,  -2.216) | -2.441  (-2.632,  -2.249) | -2.321  (-2.754,  -1.886) | -2.241  (-2.525,  -1.957) |
| 5-39 | -2.696  (-2.87,  -2.521) | -2.659  (-2.771,  -2.546) | -3.372  (-3.828,  -2.914) | -3.312  (-3.587,  -3.037) | -4.746  (-5.163,  -4.326) | -4.746  (-5.163,  -4.326) | -2.761  (-2.958,  -2.564) | -2.73  (-2.847,  -2.614) | -2.361  (-2.551,  -2.17) | -2.322  (-2.465,  -2.179) | -2.256  (-2.565,  -1.946) | -2.173  (-2.384,  -1.962) |
| 40-44 | -2.766  (-2.898,  -2.633) | -2.739  (-2.828,  -2.649) | -4.099  (-4.421,  -3.775) | -4.059  (-4.262,  -3.855) | -5.035  (-5.34,  -4.73) | -5.035  (-5.34,  -4.73) | -2.815  (-2.967,  -2.663) | -2.793  (-2.888,  -2.699) | -2.201  (-2.348,  -2.053) | -2.167  (-2.283,  -2.051) | -2.208  (-2.443,  -1.972) | -2.134  (-2.303,  -1.966) |
| 45-49 | -2.908  (-3.014,  -2.803) | -2.882  (-2.957,  -2.807) | -4.731  (-4.975,  -4.487) | -4.707  (-4.869,  -4.544) | -5.055  (-5.285,  -4.824) | -5.055  (-5.285,  -4.824) | -2.882  (-3.004,  -2.76) | -2.866  (-2.946,  -2.786) | -2.16  (-2.28,  -2.039) | -2.129  (-2.229,  -2.029) | -2.322  (-2.51,  -2.134) | -2.258  (-2.4,  -2.115) |
| 50-54 | -3.077  (-3.164,  -2.99) | -3.058  (-3.124,  -2.992) | -5.331  (-5.524,  -5.139) | -5.309  (-5.445,  -5.172) | -5.135  (-5.316,  -4.954) | -5.135  (-5.316,  -4.954) | -2.901  (-3.003,  -2.8) | -2.888  (-2.959,  -2.817) | -2.116  (-2.217,  -2.014) | -2.099  (-2.188,  -2.009) | -2.469  (-2.626,  -2.311) | -2.418  (-2.544,  -2.292) |
| 55-59 | -3.223  (-3.298,  -3.148) | -3.205  (-3.266,  -3.145) | -5.873  (-6.03,  -5.716) | -5.836  (-5.955,  -5.717) | -5.019  (-5.168,  -4.87) | -5.019  (-5.168,  -4.87) | -2.955  (-3.044,  -2.867) | -2.943  (-3.009,  -2.877) | -2.117  (-2.205,  -2.028) | -2.107  (-2.192,  -2.023) | -2.551  (-2.688,  -2.415) | -2.511  (-2.628,  -2.394) |
| 60-64 | -3.313  (-3.38,  -3.246) | -3.301  (-3.36,  -3.242) | -6.383  (-6.515,  -6.251) | -6.339  (-6.447,  -6.23) | -4.902  (-5.031,  -4.774) | -4.902  (-5.031,  -4.774) | -2.921  (-3.001,  -2.841) | -2.913  (-2.978,  -2.849) | -2.103  (-2.185,  -2.022) | -2.102  (-2.186,  -2.018) | -2.432  (-2.555,  -2.309) | -2.406  (-2.52,  -2.292) |
| 65-69 | -3.35  (-3.413,  -3.288) | -3.344  (-3.404,  -3.285) | -6.771  (-6.887,  -6.655) | -6.74  (-6.844,  -6.637) | -4.721  (-4.836,  -4.606) | -4.721  (-4.836,  -4.606) | -2.853  (-2.928,  -2.779) | -2.844  (-2.91,  -2.778) | -2.114  (-2.193,  -2.034) | -2.121  (-2.211,  -2.032) | -2.249  (-2.366,  -2.131) | -2.237  (-2.356,  -2.117) |
| 70-74 | -3.276  (-3.338,  -3.215) | -3.276  (-3.341,  -3.21) | -7.056  (-7.162,  -6.949) | -7.051  (-7.156,  -6.946) | -4.431  (-4.543,  -4.32) | -4.431  (-4.543,  -4.32) | -2.658  (-2.733,  -2.584) | -2.654  (-2.728,  -2.581) | -2.025  (-2.107,  -1.942) | -2.048  (-2.151,  -1.945) | -1.899  (-2.021,  -1.778) | -1.914  (-2.05,  -1.777) |
| 75-79 | -3.142  (-3.207,  -3.077) | -3.155  (-3.231,  -3.079) | -7.108  (-7.21,  -7.005) | -7.123  (-7.234,  -7.013) | -4.157  (-4.27,  -4.043) | -4.157  (-4.27,  -4.043) | -2.362  (-2.442,  -2.283) | -2.377  (-2.464,  -2.29) | -1.803  (-1.896,  -1.71) | -1.842  (-1.97,  -1.713) | -1.49  (-1.629,  -1.351) | -1.551  (-1.724,  -1.377) |

**Supplementary TABLE 10. (Continued)**

|  | Global | | High SDI ^c^ | | High-middle SDI ^c^ | | Middle SDI ^c^ | | Low-middle SDI ^c^ | | Low SDI ^c^ | |
| --- | --- | --- | --- | --- | --- | --- | --- | --- | --- | --- | --- | --- |
|  | Deaths rates | DALYs rates | Deaths rates | DALYs rates | Deaths rates | DALYs rates | Deaths rates | DALYs rates | Deaths rates | DALYs rates | Deaths rates | DALYs rates |
| 80-84 | -2.954  (-3.027,  -2.881) | -2.988  (-3.083,  -2.893) | -6.9  (-7.003,  -6.797) | -6.949  (-7.071,  -6.827) | -3.697  (-3.818,  -3.575) | -3.697  (-3.818,  -3.575) | -1.984  (-2.077,  -1.892) | -2.026  (-2.139,  -1.913) | -1.494  (-1.607,  -1.381) | -1.546  (-1.718,  -1.373) | -1.095  (-1.272,  -0.919) | -1.196  (-1.44,  -0.951) |
| 85-89 | -2.824  (-2.918,  -2.73) | -2.866  (-3.001,  -2.73) | -6.515  (-6.633,  -6.397) | -6.584  (-6.737,  -6.43) | -3.445  (-3.598,  -3.291) | -3.445  (-3.598,  -3.291) | -1.672  (-1.794,  -1.55) | -1.712  (-1.876,  -1.549) | -1.192  (-1.347,  -1.037) | -1.254  (-1.516,  -0.992) | -0.734  (-0.996,  -0.471) | -0.854  (-1.256,  -0.45) |
| 90-94 | -2.895  (-3.045,  -2.744) | -2.932  (-3.167,  -2.697) | -6.252  (-6.416,  -6.088) | -6.32  (-6.554,  -6.085) | -3.335  (-3.587,  -3.083) | -3.335  (-3.587,  -3.083) | -1.72  (-1.92,  -1.52) | -1.753  (-2.042,  -1.463) | -1.071  (-1.326,  -0.816) | -1.132  (-1.593,  -0.669) | -0.496  (-0.966,  -0.025) | -0.615  (-1.389,  0.166) |
| 95+ | -3.38  (-3.697,  -3.061) | -3.426  (-3.942,  -2.907) | -6.248  (-6.549,  -5.945) | -6.325  (-6.784,  -5.864) | -3.923  (-4.482,  -3.36) | -3.923  (-4.482,  -3.36) | -2.37  (-2.814,  -1.923) | -2.41  (-3.076,  -1.739) | -1.154  (-1.687,  -0.619) | -1.216  (-2.216,  -0.205) | -0.337  (-1.39,  0.728) | -0.45  (-2.242,  1.375) |
| Age group (female) Local drifts ^e^ (95% CI) ^f^ | | | | | | | | | | | | |
| 25-29 | -3.399  (-3.931,  -2.863) | -3.41  (-3.72,  -3.099) | -3.05  (-5.032,  -1.026) | -2.974  (-4.075,  -1.861) | -5.406  (-7.423,  -3.345) | -5.303  (-6.297,  -4.298) | -3.736  (-4.603,  -2.861) | -3.768  (-4.266,  -3.268) | -3.792  (-4.333,  -3.249) | -3.768  (-4.266,  -3.268) | -3.632  (-4.169,  -3.092) | -3.611  (-3.974,  -3.247) |
| 30-34 | -3.284  (-3.576,  -2.992) | -3.288  (-3.462,  -3.113) | -2.84  (-3.827,  -1.843) | -2.79  (-3.354,  -2.224) | -5.271  (-6.192,  -4.341) | -5.164  (-5.629,  -4.696) | -3.783  (-4.249,  -3.314) | -3.783  (-4.058,  -3.507) | -3.525  (-3.829,  -3.219) | -3.783  (-4.058,  -3.507) | -3.461  (-3.672,  -3.048) | -3.319  (-3.536,  -3.101) |
| 5-39 | -3.129  (-3.34,  -2.918) | -3.124  (-3.256,  -2.993) | -3.232  (-3.888,  -2.571) | -3.184  (-3.573,  -2.794) | -5.389  (-6.005,  -4.77) | -5.283  (-5.605,  -4.96) | -3.637  (-3.971,  -3.302) | -3.625  (-3.83,  -3.42) | -3.206  (-3.43,  -2.981) | -3.625  (-3.83,  -3.42) | -3.185  (-3.418,  -2.951) | -3.136  (-3.305,  -2.966) |
| 40-44 | -3.058  (-3.222,  -2.894) | -3.062  (-3.168,  -2.955) | -3.799  (-4.276,  -3.318) | -3.755  (-4.051,  -3.458) | -5.484  (-5.936,  -5.03) | -5.4  (-5.647,  -5.152) | -3.545  (-3.803,  -3.286) | -3.551  (-3.717,  -3.385) | -2.98  (-3.157,  -2.801) | -3.551  (-3.717,  -3.385) | -3.098  (-3.283,  -2.913) | -3.05  (-3.191,  -2.909) |
| 45-49 | -3.126  (-3.256,  -2.996) | -3.133  (-3.223,  -3.044) | -4.223  (-4.785,  -4.059) | -4.373  (-4.609,  -4.137) | -5.369  (-5.702,  -5.035) | -5.28  (-5.473,  -5.088) | -3.539  (-3.741,  -3.336) | -3.554  (-3.691,  -3.416) | -2.83  (-2.975,  -2.685) | -3.554  (-3.691,  -3.416) | -3.104  (-3.253,  -2.955) | -3.06  (-3.18,  -2.941) |
| 50-54 | -3.324  (-3.429,  -3.219) | -3.323  (-3.4,  -3.246) | -5.147  (-5.427,  -4.866) | -5.067  (-5.261,  -4.874) | -5.386  (-5.638,  -5.134) | -5.284  (-5.438,  -5.129) | -3.611  (-3.774,  -3.447) | -3.616  (-3.735,  -3.498) | -2.777  (-2.897,  -2.656) | -3.616  (-3.735,  -3.498) | -3.162  (-3.285,  -3.038) | -3.123  (-3.228,  -3.019) |

**Supplementary TABLE 10. (Continued)**

|  | Global | | High SDI ^c^ | | High-middle SDI ^c^ | | Middle SDI ^c^ | | Low-middle SDI ^c^ | | Low SDI ^c^ | |
| --- | --- | --- | --- | --- | --- | --- | --- | --- | --- | --- | --- | --- |
|  | Deaths rates | DALYs rates | Deaths rates | DALYs rates | Deaths rates | DALYs rates | Deaths rates | DALYs rates | Deaths rates | DALYs rates | Deaths rates | DALYs rates |
| 55-59 | -3.463  (-3.549,  -3.377) | -3.465  (-3.532,  -3.397) | -5.899  (-6.117,  -5.68) | -5.787  (-5.947,  -5.626) | -5.298  (-5.492,  -5.104) | -5.201  (-5.329,  -5.074) | -3.644  (-3.778,  -3.509) | -3.655  (-3.759,  -3.55) | -2.735  (-2.836,  -2.633) | -3.655  (-3.759,  -3.55) | -3.117  (-3.221,  -3.014) | -3.092  (-3.186,  -2.997) |
| 60-64 | -3.607  (-3.68,  -3.535) | -3.597  (-3.659,  -3.536) | -6.606  (-6.778,  -6.434) | -6.479  (-6.615,  -6.343) | -5.243  (-5.396,  -5.09) | -5.154  (-5.262,  -5.046) | -3.603  (-3.718,  -3.487) | -3.607  (-3.704,  -3.511) | -2.8  (-2.889,  -2.71) | -3.607  (-3.704,  -3.511) | -3.016  (-3.106,  -2.926) | -2.987  (-3.076,  -2.898) |
| 65-69 | -3.63  (-3.692,  -3.567) | -3.61  (-3.668,  -3.552) | -7.113  (-7.25,  -6.976) | -7  (-7.117,  -6.882) | -5.057  (-5.177,  -4.936) | -4.987  (-5.081,  -4.894) | -3.497  (-3.597,  -3.396) | -3.478  (-3.57,  -3.386) | -2.759  (-2.841,  -2.677) | -3.478  (-3.57,  -3.386) | -2.778  (-2.861,  -2.696) | -2.748  (-2.837,  -2.659) |
| 70-74 | -3.533  (-3.589,  -3.476) | -3.502  (-3.56,  -3.444) | -7.35  (-7.462,  -7.238) | -7.274  (-7.379,  -7.168) | -4.798  (-4.9,  -4.697) | -4.743  (-4.83,  -4.655) | -3.26  (-3.352,  -3.168) | -3.217  (-3.31,  -3.123) | -2.652  (-2.731,  -2.572) | -3.217  (-3.31,  -3.123) | -2.455  (-2.536,  -2.375) | -2.429  (-2.526,  -2.333) |
| 75-79 | -3.487  (-3.541,  -3.434) | -3.449  (-3.51,  -3.388) | -7.358  (-7.452,  -7.264) | -7.315  (-7.414,  -7.217) | -4.602  (-4.692,  -4.511) | -4.585  (-4.672,  -4.498) | -2.983  (-3.074,  -2.893) | -2.929  (-3.031,  -2.827) | -2.5  (-2.584,  -2.416) | -2.929  (-3.031,  -2.827) | -2.132  (-2.219,  -2.046) | -2.128  (-2.244,  -2.013) |
| 80-84 | -3.496  (-3.55,  -3.442) | -3.46  (-3.528,  -3.391) | -7.134  (-7.215,  -7.053) | -7.137  (-7.232,  -7.042) | -4.343  (-4.427,  -4.26) | -4.364  (-4.454,  -4.274) | -2.726  (-2.821,  -2.63) | -2.664  (-2.785,  -2.543) | -2.244  (-2.342,  -2.146) | -2.664  (-2.785,  -2.543) | -1.937  (-2.042,  -1.831) | -1.956  (-2.111,  -1.8) |
| 85-89 | -3.632  (-3.694,  -3.569) | -3.592  (-3.679,  -3.505) | -6.769  (-6.848,  -6.691) | -6.802  (-6.904,  -6.7) | -4.302  (-4.396,  -4.208) | -4.343  (-4.454,  -4.231) | -2.594  (-2.71,  -2.477) | -2.526  (-2.689,  -2.363) | -2.119  (-2.249,  -1.988) | -2.526  (-2.689,  -2.363) | -1.852  (-2,  -1.703) | -1.883  (-2.125,  -1.64) |
| 90-94 | -3.85  (-3.938,  -3.762) | -3.813  (-3.945,  -3.681) | -6.5  (-6.591,  -6.408) | -6.546  (-6.673,  -6.418) | -4.299  (-4.431,  -4.166) | -4.356  (-4.525,  -4.187) | -2.571  (-2.75,  -2.393) | -2.51  (-2.779,  -2.24) | -2.187  (-2.394,  -1.98) | -2.51  (-2.779,  -2.24) | -1.793  (-2.04,  -1.545) | -1.827  (-2.262,  -1.389) |
| 95+ | -4.184  (-4.342,  -4.026) | -4.155  (-4.404,  -3.906) | -6.373  (-6.51,  -6.236) | -6.434  (-6.635,  -6.232) | -4.581  (-4.829,  -4.332) | -4.652  (-4.984,  -4.319) | -2.572  (-2.919,  -2.223) | -2.525  (-3.07,  -1.978) | -2.351  (-2.757,  -1.943) | -2.525  (-3.07,  -1.978) | -1.7  (-2.194,  -1.203) | -1.742  (-2.644,  -0.831) |

**Note:** ^a^ IHD = ischemic heart disease;

^b^ DALYs = disability adjusted life years.

^c^ SDI = socio-demographic index.

^d^ Net drifts represent the overall annual percentage change in the age-standardized rate based on period and birth cohort.

^e^ Local drifts indicate the annual percentage change over time specific to the age group.

^f^ CI = confidence interval. All net and local drifts were statistically significant (*P*<0.05).

**Supplementary Table 11:** Net and local drifts for IHD ^a^ deaths and DALYs ^b^ rates attributed to non-optimal temperature (%).

|  | Global | | High SDI ^c^ | | High-middle SDI ^c^ | | Middle SDI ^c^ | | Low-middle SDI ^c^ | | Low SDI ^c^ | |
| --- | --- | --- | --- | --- | --- | --- | --- | --- | --- | --- | --- | --- |
|  | Deaths rates | DALYs rates | Deaths rates | DALYs rates | Deaths rates | DALYs rates | Deaths rates | DALYs rates | Deaths rates | DALYs rates | Deaths rates | DALYs rates |
| Sex Net drifts ^d^ (95% CI) ^f^ | | | | | | | | | | | | |
| Both | -2.975  (-3.026,  -2.923) | -2.975  (-3.012,  -2.939) | -4.26  (-4.436,  -4.082) | -4.262  (-4.373,  -4.15) | -4.037  (-4.205,  -3.869) | -4.031  (-4.123,  -3.939) | -2.345  (-2.408,  -2.283) | -2.345  (-2.392,  -2.298) | -1.733  (-1.794,  -1.672) | -1.731  (-1.789,  -1.673) | -2.226  (-2.35,  -2.103) | -2.209  (-2.325,  -2.092) |
| Male | -2.828  (-2.907,  -2.748) | -2.828  (-2.886,  -2.77) | -4.265  (-4.448,  -4.082) | -4.272  (-4.388,  -4.156) | -3.919  (-4.082,  -3.756) | -3.922  (-4.017,  -3.826) | -2.031  (-2.111,  -1.952) | -2.031  (-2.092,  -1.969) | -1.368  (-1.441,  -1.295) | -1.365  (-1.437,  -1.293) | -1.844  (-1.995,  -1.693) | -1.826  (-1.973,  -1.679) |
| Female | -3.164  (-3.225,  -3.103) | -3.17  (-3.212,  -3.128) | -4.351  (-4.571,  -4.131) | -4.34  (-4.482,  -4.197) | -4.297  (-4.534,  -4.059) | -4.277  (-4.407,  -4.147) | -2.836  (-2.933,  -2.74) | -2.842  (-2.913,  -2.77) | -2.189  (-2.264,  -2.114) | -2.191  (-2.26,  -2.122) | -2.702  (-2.821,  -2.583) | -2.685  (-2.799,  -2.571) |
| Age group (Both) Local drifts ^e^ (95% CI) ^f^ | | | | | | | | | | | | |
| 15-19 | -2.65  (-3.327,  -1.968) | -2.631  (-3.018,  -2.244) | -3.89  (-7.16,  -0.505) | -3.836  (-5.719,  -1.915) | -3.854  (-6.727,  -0.892) | -3.828  (-5.221,  -2.415) | -3.083  (-3.916,  -2.244) | -3.092  (-3.569,  -2.612) | -2.307  (-2.955,  -1.655) | -2.291  (-2.701,  -1.878) | -3.111  (-4.254,  -1.954) | -3.075  (-3.772,  -2.373) |
| 20-24 | -2.399  (-2.759,  -2.038) | -2.389  (-2.6,  -2.179) | -2.912  (-4.435,  -1.365) | -2.878  (-3.772,  -1.975) | -3.532  (-4.911,  -2.134) | -3.509  (-4.188,  -2.824) | -2.708  (-3.136,  -2.278) | -2.71  (-2.961,  -2.458) | -2.227  (-2.581,  -1.871) | -2.215  (-2.445,  -1.984) | -3.095  (-3.771,  -2.414) | -3.039  (-3.462,  -2.614) |
| 25-29 | -2.301  (-2.547,  -2.054) | -2.285  (-2.434,  -2.136) | -2.366  (-3.268,  -1.455) | -2.322  (-2.868,  -1.773) | -3.619  (-4.468,  -2.762) | -3.594  (-4.024,  -3.161) | -2.44  (-2.724,  -2.155) | -2.433  (-2.605,  -2.26) | -2.121  (-2.374,  -1.867) | -2.102  (-2.273,  -1.931) | -2.857  (-3.363,  -2.347) | -2.792  (-3.119,  -2.463) |
| 30-34 | -2.37  (-2.55,  -2.19) | -2.348  (-2.461,  -2.235) | -2.316  (-2.89,  -1.738) | -2.26  (-2.621,  -1.897) | -3.961  (-4.513,  -3.407) | -3.932  (-4.223,  -3.64) | -2.408  (-2.614,  -2.201) | -2.391  (-2.522,  -2.261) | -1.949  (-2.142,  -1.755) | -1.924  (-2.06,  -1.789) | -2.638  (-3.036,  -2.238) | -2.57  (-2.838,  -2.301) |
| 5-39 | -2.486  (-2.624,  -2.348) | -2.463  (-2.554,  -2.373) | -2.682  (-3.074,  -2.288) | -2.626  (-2.884,  -2.368) | -4.312  (-4.709,  -3.914) | -4.279  (-4.497,  -4.06) | -2.412  (-2.572,  -2.252) | -2.394  (-2.5,  -2.289) | -1.81  (-1.964,  -1.656) | -1.785  (-1.898,  -1.672) | -2.489  (-2.806,  -2.171) | -2.418  (-2.642,  -2.194) |
| 40-44 | -2.621  (-2.729,  -2.513) | -2.607  (-2.681,  -2.532) | -3.144  (-3.425,  -2.862) | -3.1  (-3.294,  -2.906) | -4.559  (-4.853,  -4.263) | -4.534  (-4.704,  -4.364) | -2.456  (-2.583,  -2.328) | -2.448  (-2.537,  -2.359) | -1.692  (-1.817,  -1.566) | -1.667  (-1.764,  -1.57) | -2.482  (-2.736,  -2.226) | -2.419  (-2.608,  -2.23) |

**Supplementary TABLE 11. (Continued)**

|  | Global | | High SDI ^c^ | | High-middle SDI ^c^ | | Middle SDI ^c^ | | Low-middle SDI ^c^ | | Low SDI ^c^ | |
| --- | --- | --- | --- | --- | --- | --- | --- | --- | --- | --- | --- | --- |
|  | Deaths rates | DALYs rates | Deaths rates | DALYs rates | Deaths rates | DALYs rates | Deaths rates | DALYs rates | Deaths rates | DALYs rates | Deaths rates | DALYs rates |
| 45-49 | -2.758  (-2.844,  -2.672) | -2.747  (-2.81,  -2.684) | -3.593  (-3.803,  -3.382) | -3.565  (-3.718,  -3.412) | -4.552  (-4.774,  -4.329) | -4.525  (-4.661,  -4.389) | -2.51  (-2.613,  -2.408) | -2.507  (-2.583,  -2.432) | -1.665  (-1.768,  -1.56) | -1.645  (-1.73,  -1.56) | -2.599  (-2.806,  -2.391) | -2.545  (-2.707,  -2.382) |
| 50-54 | -2.96  (-3.03,  -2.89) | -2.953  (-3.007,  -2.898) | -3.971  (-4.133,  -3.809) | -3.954  (-4.08,  -3.829) | -4.653  (-4.825,  -4.48) | -4.626  (-4.738,  -4.513) | -2.567  (-2.652,  -2.483) | -2.564  (-2.631,  -2.498) | -1.641  (-1.729,  -1.552) | -1.627  (-1.704,  -1.551) | -2.753  (-2.927,  -2.578) | -2.71  (-2.854,  -2.565) |
| 55-59 | -3.079  (-3.138,  -3.021) | -3.076  (-3.125,  -3.028) | -4.335  (-4.464,  -4.206) | -4.322  (-4.429,  -4.214) | -4.423  (-4.56,  -4.285) | -4.399  (-4.495,  -4.303) | -2.637  (-2.708,  -2.565) | -2.638  (-2.698,  -2.578) | -1.653  (-1.729,  -1.576) | -1.644  (-1.716,  -1.573) | -2.809  (-2.959,  -2.658) | -2.781  (-2.914,  -2.647) |
| 60-64 | -3.209  (-3.259,  -3.158) | -3.207  (-3.252,  -3.162) | -4.804  (-4.91,  -4.698) | -4.796  (-4.891,  -4.701) | -4.235  (-4.348,  -4.121) | -4.21  (-4.296,  -4.124) | -2.626  (-2.689,  -2.563) | -2.633  (-2.691,  -2.576) | -1.699  (-1.769,  -1.629) | -1.691  (-1.762,  -1.621) | -2.628  (-2.763,  -2.493) | -2.613  (-2.743,  -2.483) |
| 65-69 | -3.324  (-3.369,  -3.279) | -3.327  (-3.371,  -3.283) | -5.266  (-5.356,  -5.176) | -5.277  (-5.365,  -5.189) | -4.098  (-4.195,  -4.001) | -4.083  (-4.164,  -4.003) | -2.548  (-2.605,  -2.491) | -2.549  (-2.606,  -2.491) | -1.779  (-1.845,  -1.712) | -1.776  (-1.85,  -1.703) | -2.297  (-2.425,  -2.17) | -2.291  (-2.425,  -2.156) |
| 70-74 | -3.307  (-3.349,  -3.265) | -3.315  (-3.361,  -3.27) | -5.625  (-5.704,  -5.546) | -5.666  (-5.751,  -5.582) | -3.798  (-3.887,  -3.709) | -3.789  (-3.87,  -3.707) | -2.328  (-2.383,  -2.273) | -2.321  (-2.382,  -2.261) | -1.797  (-1.864,  -1.73) | -1.807  (-1.889,  -1.725) | -1.815  (-1.945,  -1.686) | -1.816  (-1.968,  -1.665) |
| 75-79 | -3.308  (-3.349,  -3.267) | -3.327  (-3.376,  -3.277) | -5.792  (-5.864,  -5.72) | -5.85  (-5.935,  -5.765) | -3.603  (-3.688,  -3.518) | -3.623  (-3.709,  -3.537) | -2.001  (-2.057,  -1.944) | -2.003  (-2.072,  -1.933) | -1.72  (-1.793,  -1.647) | -1.744  (-1.843,  -1.644) | -1.383  (-1.528,  -1.238) | -1.411  (-1.599,  -1.222) |
| 80-84 | -3.242  (-3.284,  -3.2) | -3.275  (-3.331,  -3.218) | -5.677  (-5.744,  -5.609) | -5.756  (-5.845,  -5.667) | -3.264  (-3.347,  -3.18) | -3.316  (-3.41,  -3.221) | -1.607  (-1.67,  -1.543) | -1.623  (-1.71,  -1.536) | -1.525  (-1.612,  -1.438) | -1.56  (-1.692,  -1.427) | -1.122  (-1.301,  -0.942) | -1.18  (-1.439,  -0.921) |
| 85-89 | -3.254  (-3.304,  -3.205) | -3.288  (-3.362,  -3.215) | -5.322  (-5.393,  -5.252) | -5.412  (-5.515,  -5.31) | -3.246  (-3.345,  -3.147) | -3.311  (-3.434,  -3.188) | -1.328  (-1.408,  -1.247) | -1.338  (-1.461,  -1.215) | -1.354  (-1.472,  -1.235) | -1.394  (-1.591,  -1.196) | -0.941  (-1.201,  -0.68) | -1.016  (-1.431,  -0.6) |
| 90-94 | -3.379  (-3.449,  -3.308) | -3.413  (-3.525,  -3.3) | -4.969  (-5.058,  -4.88) | -5.055  (-5.193,  -4.917) | -3.261  (-3.408,  -3.113) | -3.334  (-3.533,  -3.136) | -1.431  (-1.559,  -1.303) | -1.442  (-1.65,  -1.234) | -1.308  (-1.501,  -1.115) | -1.35  (-1.695,  -1.004) | -0.741  (-1.194,  -0.285) | -0.818  (-1.598,  -0.032) |

**Supplementary TABLE 11. (Continued)**

|  | Global | | High SDI ^c^ | | High-middle SDI ^c^ | | Middle SDI ^c^ | | Low-middle SDI ^c^ | | Low SDI ^c^ | |
| --- | --- | --- | --- | --- | --- | --- | --- | --- | --- | --- | --- | --- |
|  | Deaths rates | DALYs rates | Deaths rates | DALYs rates | Deaths rates | DALYs rates | Deaths rates | DALYs rates | Deaths rates | DALYs rates | Deaths rates | DALYs rates |
| 95+ | -3.734  (-3.863,  -3.606) | -3.779  (-3.991,  -3.565) | -4.787  (-4.93,  -4.643) | -4.877  (-5.111,  -4.641) | -3.648  (-3.937,  -3.358) | -3.739  (-4.144,  -3.332) | -1.857  (-2.117,  -1.596) | -1.884  (-2.322,  -1.444) | -1.445  (-1.834,  -1.054) | -1.49  (-2.213,  -0.763) | -0.535  (-1.501,  0.44) | -0.618  (-2.332,  1.126) |
| Age group (male) Local drifts ^e^ (95% CI) ^f^ | | | | | | | | | | | | |
| 15-19 | -2.504  (-3.607,  -1.389) | -2.462  (-3.085,  -1.834) | -3.788  (-7.235,  -0.212) | -3.715  (-5.691,  -1.697) | -3.731  (-6.559,  -0.816) | -3.707  (-5.114,  -2.279) | -2.856  (-3.933,  -1.768) | -2.844  (-3.45,  -2.233) | -1.765  (-2.591,  -0.932) | -1.733  (-2.269,  -1.194) | -2.48  (-4.007,  -0.927) | -2.424  (-3.36,  -1.478) |
| 20-24 | -2.203  (-2.769,  -1.634) | -2.171  (-2.499,  -1.842) | -2.854  (-4.444,  -1.237) | -2.8  (-3.729,  -1.862) | -3.365  (-4.702,  -2.01) | -3.346  (-4.022,  -2.664) | -2.398  (-2.942,  -1.851) | -2.389  (-2.702,  -2.074) | -1.741  (-2.175,  -1.305) | -1.712  (-2.002,  -1.422) | -2.47  (-3.35,  -1.582) | -2.395  (-2.949,  -1.838) |
| 25-29 | -2.099  (-2.475,  -1.722) | -2.063  (-2.288,  -1.838) | -2.343  (-3.266,  -1.41) | -2.281  (-2.838,  -1.721) | -3.469  (-4.279,  -2.652) | -3.444  (-3.866,  -3.02) | -2.078  (-2.432,  -1.724) | -2.063  (-2.273,  -1.852) | -1.694  (-1.994,  -1.393) | -1.659  (-1.866,  -1.451) | -2.304  (-2.941,  -1.663) | -2.224  (-2.639,  -1.808) |
| 30-34 | -2.226  (-2.493,  -1.959) | -2.182  (-2.348,  -2.016) | -2.357  (-2.935,  -1.776) | -2.287  (-2.648,  -1.924) | -3.862  (-4.379,  -3.342) | -3.833  (-4.114,  -3.552) | -2.049  (-2.3,  -1.796) | -2.021  (-2.178,  -1.865) | -1.611  (-1.833,  -1.388) | -1.569  (-1.729,  -1.409) | -2.24  (-2.72,  -1.756) | -2.151  (-2.476,  -1.824) |
| 5-39 | -2.41  (-2.609,  -2.21) | -2.367  (-2.496,  -2.236) | -2.788  (-3.177,  -2.397) | -2.721  (-2.975,  -2.466) | -4.243  (-4.611,  -3.874) | -4.21  (-4.418,  -4.002) | -2.086  (-2.278,  -1.893) | -2.053  (-2.178,  -1.928) | -1.526  (-1.7,  -1.352) | -1.488  (-1.619,  -1.357) | -2.165  (-2.535,  -1.792) | -2.074  (-2.337,  -1.81) |
| 40-44 | -2.582  (-2.736,  -2.427) | -2.548  (-2.654,  -2.443) | -3.285  (-3.562,  -3.007) | -3.236  (-3.426,  -3.046) | -4.514  (-4.786,  -4.242) | -4.49  (-4.651,  -4.328) | -2.159  (-2.312,  -2.006) | -2.132  (-2.237,  -2.028) | -1.4  (-1.54,  -1.26) | -1.365  (-1.475,  -1.254) | -2.141  (-2.432,  -1.85) | -2.063  (-2.28,  -1.845) |
| 45-49 | -2.725  (-2.848,  -2.602) | -2.697  (-2.786,  -2.608) | -3.724  (-3.931,  -3.517) | -3.696  (-3.846,  -3.546) | -4.507  (-4.713,  -4.3) | -4.482  (-4.612,  -4.353) | -2.229  (-2.352,  -2.106) | -2.208  (-2.298,  -2.119) | -1.381  (-1.497,  -1.265) | -1.35  (-1.447,  -1.252) | -2.28  (-2.515,  -2.045) | -2.213  (-2.398,  -2.028) |
| 50-54 | -2.901  (-3.002,  -2.8) | -2.884  (-2.962,  -2.807) | -4.065  (-4.226,  -3.905) | -4.053  (-4.177,  -3.929) | -4.6  (-4.762,  -4.438) | -4.581  (-4.689,  -4.472) | -2.269  (-2.371,  -2.167) | -2.255  (-2.334,  -2.175) | -1.343  (-1.442,  -1.244) | -1.323  (-1.411,  -1.235) | -2.461  (-2.658,  -2.263) | -2.41  (-2.575,  -2.244) |
| 55-59 | -3.006  (-3.091,  -2.92) | -2.993  (-3.064,  -2.922) | -4.373  (-4.502,  -4.244) | -4.367  (-4.474,  -4.26) | -4.348  (-4.48,  -4.216) | -4.328  (-4.423,  -4.233) | -2.344  (-2.432,  -2.255) | -2.334  (-2.407,  -2.26) | -1.355  (-1.443,  -1.268) | -1.343  (-1.427,  -1.26) | -2.56  (-2.732,  -2.388) | -2.525  (-2.679,  -2.371) |

**Supplementary TABLE 11. (Continued)**

|  | Global | | High SDI ^c^ | | High-middle SDI ^c^ | | Middle SDI ^c^ | | Low-middle SDI ^c^ | | Low SDI ^c^ | |
| --- | --- | --- | --- | --- | --- | --- | --- | --- | --- | --- | --- | --- |
|  | Deaths rates | DALYs rates | Deaths rates | DALYs rates | Deaths rates | DALYs rates | Deaths rates | DALYs rates | Deaths rates | DALYs rates | Deaths rates | DALYs rates |
| 60-64 | -3.106  (-3.181,  -3.031) | -3.101  (-3.168,  -3.033) | -4.786  (-4.894,  -4.678) | -4.787  (-4.884,  -4.69) | -4.137  (-4.25,  -4.025) | -4.121  (-4.209,  -4.033) | -2.339  (-2.419,  -2.259) | -2.338  (-2.41,  -2.267) | -1.363  (-1.443,  -1.282) | -1.357  (-1.441,  -1.273) | -2.361  (-2.516,  -2.205) | -2.347  (-2.498,  -2.196) |
| 65-69 | -3.217  (-3.285,  -3.148) | -3.224  (-3.292,  -3.156) | -5.219  (-5.313,  -5.124) | -5.242  (-5.334,  -5.149) | -3.991  (-4.092,  -3.89) | -3.994  (-4.081,  -3.907) | -2.27  (-2.344,  -2.196) | -2.27  (-2.343,  -2.197) | -1.459  (-1.537,  -1.38) | -1.463  (-1.552,  -1.374) | -2.053  (-2.202,  -1.903) | -2.053  (-2.212,  -1.894) |
| 70-74 | -3.206  (-3.273,  -3.138) | -3.227  (-3.3,  -3.154) | -5.6  (-5.686,  -5.514) | -5.656  (-5.748,  -5.563) | -3.681  (-3.78,  -3.582) | -3.688  (-3.783,  -3.594) | -2.053  (-2.127,  -1.978) | -2.058  (-2.138,  -1.977) | -1.479  (-1.561,  -1.398) | -1.5  (-1.602,  -1.397) | -1.541  (-1.696,  -1.387) | -1.56  (-1.742,  -1.378) |
| 75-79 | -3.156  (-3.225,  -3.086) | -3.2  (-3.284,  -3.117) | -5.753  (-5.836,  -5.67) | -5.832  (-5.93,  -5.734) | -3.469  (-3.571,  -3.367) | -3.508  (-3.615,  -3.401) | -1.693  (-1.772,  -1.614) | -1.72  (-1.816,  -1.624) | -1.363  (-1.454,  -1.272) | -1.397  (-1.524,  -1.27) | -1.049  (-1.226,  -0.872) | -1.104  (-1.336,  -0.872) |
| 80-84 | -2.979  (-3.055,  -2.903) | -3.051  (-3.152,  -2.949) | -5.6  (-5.684,  -5.516) | -5.705  (-5.815,  -5.595) | -3.067  (-3.176,  -2.958) | -3.144  (-3.271,  -3.017) | -1.199  (-1.292,  -1.106) | -1.257  (-1.384,  -1.131) | -1.128  (-1.239,  -1.018) | -1.177  (-1.349,  -1.006) | -0.662  (-0.885,  -0.438) | -0.75  (-1.075,  -0.425) |
| 85-89 | -2.818  (-2.915,  -2.722) | -2.897  (-3.039,  -2.755) | -5.187  (-5.283,  -5.09) | -5.302  (-5.441,  -5.162) | -2.954  (-3.093,  -2.816) | -3.044  (-3.223,  -2.865) | -0.772  (-0.896,  -0.649) | -0.822  (-1.006,  -0.637) | -0.835  (-0.987,  -0.683) | -0.892  (-1.152,  -0.632) | -0.319  (-0.648,  0.012) | -0.422  (-0.953,  0.111) |
| 90-94 | -2.829  (-2.98,  -2.678) | -2.901  (-3.14,  -2.662) | -4.794  (-4.931,  -4.657) | -4.902  (-5.116,  -4.687) | -2.897  (-3.123,  -2.669) | -2.981  (-3.297,  -2.665) | -0.899  (-1.102,  -0.697) | -0.937  (-1.26,  -0.613) | -0.678  (-0.926,  -0.428) | -0.734  (-1.19,  -0.276) | -0.011  (-0.606,  0.587) | -0.112  (-1.14,  0.927) |
| 95+ | -3.278  (-3.584,  -2.971) | -3.362  (-3.867,  -2.855) | -4.666  (-4.918,  -4.412) | -4.776  (-5.194,  -4.357) | -3.414  (-3.907,  -2.918) | -3.512  (-4.226,  -2.794) | -1.831  (-2.272,  -1.388) | -1.8  (-2.611,  -1.144) | -0.754  (-1.268,  -0.237) | -0.813  (-1.789,  0.172) | 0.244  (-1.097,  1.603) | 0.145  (-2.243,  2.591) |
| Age group (female) Local drifts ^e^ (95% CI) ^f^ | | | | | | | | | | | | |
| 15-19 | -2.898  (-3.601,  -2.191) | -2.898  (-3.289,  -2.505) | -4.205  (-7.997,  -0.256) | -4.163  (-6.391,  -1.882) | -4.316  (-8.068,  -0.41) | -4.275  (-6.114,  -2.401) | -3.498  (-4.684,  -2.297) | -3.529  (-4.228,  -2.824) | -2.948  (-3.66,  -2.23) | -2.946  (-3.401,  -2.489) | -3.639  (-4.628,  -2.639) | -3.618  (-4.247,  -2.985) |
| 20-24 | -2.751  (-3.143,  -2.357) | -2.756  (-2.98,  -2.532) | -3.176  (-4.987,  -1.331) | -3.158  (-4.242,  -2.062) | -4.139  (-6.011,  -2.229) | -4.098  (-5.03,  -3.157) | -3.299  (-3.93,  -2.663) | -3.3  (-3.681,  -2.918) | -2.871  (-3.279,  -2.461) | -2.873  (-3.141,  -2.605) | -3.668  (-4.27,  -3.063) | -3.625  (-4.018,  -3.231) |

**Supplementary TABLE 11. (Continued)**

|  | Global | | High SDI ^c^ | | High-middle SDI ^c^ | | Middle SDI ^c^ | | Low-middle SDI ^c^ | | Low SDI ^c^ | |
| --- | --- | --- | --- | --- | --- | --- | --- | --- | --- | --- | --- | --- |
|  | Deaths rates | DALYs rates | Deaths rates | DALYs rates | Deaths rates | DALYs rates | Deaths rates | DALYs rates | Deaths rates | DALYs rates | Deaths rates | DALYs rates |
| 25-29 | -2.701  (-2.984,  -2.417) | -2.699  (-2.865,  -2.532) | -2.576  (-3.705,  -1.433) | -2.551  (-3.248,  -1.85) | -4.195  (-5.4,  -2.974) | -4.16  (-4.776,  -3.541) | -3.184  (-3.62,  -2.746) | -3.167  (-3.438,  -2.894) | -2.758  (-3.063,  -2.452) | -2.749  (-2.956,  -2.543) | -3.482  (-3.949,  -3.013) | -3.418  (-3.733,  -3.102) |
| 30-34 | -2.668  (-2.887,  -2.449) | -2.665  (-2.799,  -2.531) | -2.371  (-3.127,  -1.61) | -2.335  (-2.818,  -1.848) | -4.363  (-5.185,  -3.534) | -4.325  (-4.761,  -3.886) | -3.176  (-3.507,  -2.844) | -3.158  (-3.373,  -2.944) | -2.482  (-2.726,  -2.238) | -2.474  (-2.646,  -2.303) | -3.182  (-3.566,  -2.796) | -3.124  (-3.393,  -2.854) |
| 5-39 | -2.642  (-2.817,  -2.467) | -2.641  (-2.753,  -2.53) | -2.545  (-3.083,  -2.004) | -2.498  (-2.857,  -2.137) | -4.606  (-5.22,  -3.988) | -4.562  (-4.902,  -4.222) | -3.12  (-3.385,  -2.855) | -3.117  (-3.296,  -2.938) | -2.265  (-2.465,  -2.064) | -2.257  (-2.405,  -2.109) | -3.007  (-3.326,  -2.688) | -2.954  (-3.188,  -2.72) |
| 40-44 | -2.682  (-2.822,  -2.541) | -2.693  (-2.787,  -2.598) | -2.865  (-3.261,  -2.468) | -2.814  (-3.091,  -2.536) | -4.766  (-5.232,  -4.298) | -4.736  (-5.005,  -4.465) | -3.071  (-3.284,  -2.857) | -3.098  (-3.249,  -2.947) | -2.146  (-2.313,  -1.98) | -2.143  (-2.272,  -2.013) | -3.047  (-3.312,  -2.781) | -3.002  (-3.206,  -2.798) |
| 45-49 | -2.772  (-2.884,  -2.659) | -2.791  (-2.87,  -2.711) | -3.305  (-3.602,  -3.007) | -3.258  (-3.477,  -3.038) | -4.691  (-5.039,  -4.341) | -4.654  (-4.867,  -4.44) | -3.032  (-3.201,  -2.862) | -3.072  (-3.199,  -2.945) | -2.086  (-2.224,  -1.947) | -2.094  (-2.208,  -1.981) | -3.131  (-3.35,  -2.911) | -3.096  (-3.274,  -2.918) |
| 50-54 | -3.026  (-3.116,  -2.935) | -3.036  (-3.104,  -2.968) | -3.779  (-4.005,  -3.553) | -3.731  (-3.908,  -3.553) | -4.813  (-5.075,  -4.55) | -4.763  (-4.933,  -4.592) | -3.075  (-3.212,  -2.939) | -3.104  (-3.214,  -2.995) | -2.089  (-2.205,  -1.972) | -2.096  (-2.198,  -1.994) | -3.223  (-3.407,  -3.039) | -3.194  (-3.353,  -3.035) |
| 55-59 | -3.184  (-3.257,  -3.111) | -3.198  (-3.257,  -3.139) | -4.32  (-4.494,  -4.145) | -4.271  (-4.417,  -4.124) | -4.649  (-4.848,  -4.449) | -4.606  (-4.744,  -4.467) | -3.094  (-3.206,  -2.982) | -3.126  (-3.222,  -3.03) | -2.09  (-2.189,  -1.991) | -2.099  (-2.191,  -2.006) | -3.168  (-3.324,  -3.012) | -3.156  (-3.3,  -3.011) |
| 60-64 | -3.396  (-3.457,  -3.335) | -3.401  (-3.454,  -3.348) | -4.988  (-5.125,  -4.851) | -4.947  (-5.07,  -4.823) | -4.525  (-4.679,  -4.371) | -4.482  (-4.598,  -4.367) | -3.067  (-3.163,  -2.972) | -3.094  (-3.183,  -3.006) | -2.175  (-2.263,  -2.088) | -2.171  (-2.26,  -2.083) | -2.975  (-3.113,  -2.837) | -2.964  (-3.102,  -2.826) |
| 65-69 | -3.531  (-3.583,  -3.48) | -3.533  (-3.581,  -3.484) | -5.608  (-5.718,  -5.498) | -5.591  (-5.698,  -5.483) | -4.364  (-4.484,  -4.244) | -4.33  (-4.428,  -4.231) | -2.958  (-3.04,  -2.875) | -2.965  (-3.049,  -2.881) | -2.186  (-2.266,  -2.105) | -2.179  (-2.268,  -2.089) | -2.578  (-2.705,  -2.451) | -2.565  (-2.705,  -2.425) |
| 70-74 | -3.509  (-3.554,  -3.463) | -3.504  (-3.552,  -3.456) | -5.996  (-6.086,  -5.906) | -6.013  (-6.109,  -5.916) | -4.04  (-4.14,  -3.939) | -4.012  (-4.104,  -3.92) | -2.708  (-2.783,  -2.632) | -2.69  (-2.775,  -2.604) | -2.153  (-2.232,  -2.075) | -2.147  (-2.243,  -2.05) | -2.119  (-2.245,  -1.993) | -2.101  (-2.254,  -1.948) |

**Supplementary TABLE 11. (Continued)**

|  | Global | | High SDI ^c^ | | High-middle SDI ^c^ | | Middle SDI ^c^ | | Low-middle SDI ^c^ | | Low SDI ^c^ | |
| --- | --- | --- | --- | --- | --- | --- | --- | --- | --- | --- | --- | --- |
|  | Deaths rates | DALYs rates | Deaths rates | DALYs rates | Deaths rates | DALYs rates | Deaths rates | DALYs rates | Deaths rates | DALYs rates | Deaths rates | DALYs rates |
| 75-79 | -3.534  (-3.576,  -3.492) | -3.53  (-3.578,  -3.481) | -6.137  (-6.213,  -6.062) | -6.175  (-6.265,  -6.085) | -3.807  (-3.896,  -3.717) | -3.812  (-3.902,  -3.722) | -2.381  (-2.455,  -2.307) | -2.356  (-2.449,  -2.263) | -2.071  (-2.153,  -1.988) | -2.075  (-2.188,  -1.962) | -1.743  (-1.88,  -1.606) | -1.741  (-1.926,  -1.556) |
| 80-84 | -3.512  (-3.553,  -3.472) | -3.519  (-3.572,  -3.467) | -5.985  (-6.05,  -5.92) | -6.05  (-6.136,  -5.963) | -3.456  (-3.537,  -3.376) | -3.496  (-3.588,  -3.403) | -2.018  (-2.096,  -1.939) | -1.991  (-2.102,  -1.879) | -1.864  (-1.962,  -1.767) | -1.878  (-2.025,  -1.73) | -1.603  (-1.77,  -1.437) | -1.631  (-1.88,  -1.381) |
| 85-89 | -3.594  (-3.639,  -3.549) | -3.597  (-3.662,  -3.533) | -5.578  (-5.641,  -5.516) | -5.659  (-5.752,  -5.566) | -3.447  (-3.538,  -3.356) | -3.501  (-3.616,  -3.386) | -1.79  (-1.888,  -1.693) | -1.754  (-1.906,  -1.602) | -1.783  (-1.913,  -1.653) | -1.8  (-2.017,  -1.581) | -1.595  (-1.831,  -1.358) | -1.641  (-2.033,  -1.248) |
| 90-94 | -3.719  (-3.78,  -3.658) | -3.726  (-3.82,  -3.632) | -5.16  (-5.234,  -5.087) | -5.245  (-5.362,  -5.129) | -3.431  (-3.56,  -3.301) | -3.503  (-3.679,  -3.328) | -1.806  (-1.956,  -1.656) | -1.777  (-2.027,  -1.526) | -1.83  (-2.039,  -1.62) | -1.852  (-2.229,  -1.473) | -1.504  (-1.904,  -1.103) | -1.557  (-2.271,  -0.837) |
| 95+ | -3.974  (-4.08,  -3.869) | -3.989  (-4.159,  -3.819) | -4.886  (-4.997,  -4.775) | -4.979  (-5.164,  -4.795) | -3.726  (-3.97,  -3.481) | -3.815  (-4.159,  -3.47) | -1.888  (-2.179,  -1.595) | -1.878  (-2.382,  -1.371) | -1.989  (-2.404,  -1.573) | -2.017  (-2.792,  -1.236) | -1.318  (-2.13,  -0.499) | -1.387  (-2.888,  0.139) |

**Note:** ^a^ IHD = ischemic heart disease;

^b^ DALYs = disability adjusted life years.

^c^ SDI = socio-demographic index.

^d^ Net drifts represent the overall annual percentage change in the age-standardized rate based on period and birth cohort.

^e^ Local drifts indicate the annual percentage change over time specific to the age group.

^f^ CI = confidence interval. All net and local drifts were statistically significant (*P*<0.05).

**Supplementary TABLE 12** Net and local drifts for IHD a deaths and DALYs b rates attributed to lead exposure (%).

|  | Global | | High SDI ^c^ | | High-middle SDI ^c^ | | Middle SDI ^c^ | | Low-middle SDI ^c^ | | Low SDI ^c^ | |
| --- | --- | --- | --- | --- | --- | --- | --- | --- | --- | --- | --- | --- |
|  | Deaths rates | DALYs rates | Deaths rates | DALYs rates | Deaths rates | DALYs rates | Deaths rates | DALYs rate s | Deaths rates | DALYs rates | Deaths rates | DALYs rates |
| Sex Net drifts ^d^ (95% CI) ^f^ | | | | | | | | | | | | |
| Both | -2.801  (-2.862,  -2.74) | -2.788  (-2.832,  -2.743) | -5.353  (-5.533,  -5.173) | -5.307  (-5.408,  -5.205) | -3.654  (-3.782,  -3.525) | -3.619  (-3.699,  -3.54) | -2.689  (-2.76,  -2.618) | -2.675  (-2.727,  -2.622) | -2.245  (-2.321,  -2.169) | -2.24  (-2.311,  -2.169) | -2.377  (-2.488,  -2.267) | -2.361  (-2.466,  -2.255) |
| Male | -2.713  (-2.782,  -2.644) | -2.702  (-2.761,  -2.643) | -5.41  (-5.572,  -5.248) | -5.373  (-5.472,  -5.274) | -3.573  (-3.706,  -3.44) | -3.549  (-3.637,  -3.46) | -2.44  (-2.52,  -2.36) | -2.43  (-2.495,  -2.366) | -1.956  (-2.04,  -1.873) | -1.955  (-2.043,  -1.868) | -2.115  (-2.247,  -1.982) | -2.104  (-2.236,  -1.971) |
| Female | -3.001  (-3.084,  -2.917) | -2.989  (-3.042,  -2.936) | -5.447  (-5.714,  -5.178) | -5.378  (-5.523,  -5.232) | -3.995  (-4.194,  -3.797) | -3.936  (-4.046,  -3.825) | -3.148  (-3.262,  -3.035) | -3.131  (-3.205,  -3.058) | -2.638  (-2.732,  -2.544) | -2.63  (-2.707,  -2.553) | -2.758  (-2.865,  -2.652) | -2.736  (-2.833,  -2.638) |
| Age group（Both）Local drifts ^e^ (95% CI) ^f^ | | | | | | | | | | | | |
| 25-29 | -4.371  (-5.141,  -3.594) | -4.361  (-4.801,  -3.919) | -6.156  (-9.189,  -3.021) | -6.101  (-7.678,  -4.497) | -5.835  (-7.809,  -3.819) | -5.797  (-6.847,  -4.736) | -4.782  (-5.685,  -3.869) | -4.78  (-5.287,  -4.271) | -4.569  (-5.404,  -3.726) | -4.544  (-5.071,  -4.014) | -4.139  (-5.286,  -2.978) | -4.103  (-4.815,  -3.385) |
| 30-34 | -4.093  (-4.457,  -3.726) | -4.073  (-4.287,  -3.857) | -5.677  (-6.916,  -4.421) | -5.625  (-6.286,  -4.96) | -5.648  (-6.455,  -4.835) | -5.597  (-6.037,  -5.155) | -4.532  (-4.95,  -4.112) | -4.513  (-4.754,  -4.271) | -4.131  (-4.538,  -3.723) | -4.101  (-4.366,  -3.836) | -3.839  (-4.429,  -3.245) | -3.781  (-4.159,  -3.401) |
| 5-39 | -3.74  (-3.971,  -3.509) | -3.714  (-3.855,  -3.573) | -5.473  (-6.146,  -4.795) | -5.416  (-5.788,  -5.043) | -5.515  (-5.994,  -5.033) | -5.456  (-5.726,  -5.184) | -4.158  (-4.419,  -3.896) | -4.13  (-4.286,  -3.973) | -3.654  (-3.917,  -3.39) | -3.621  (-3.799,  -3.443) | -3.528  (-3.917,  -3.138) | -3.46  (-3.719,  -3.2) |
| 40-44 | -3.425  (-3.584,  -3.265) | -3.4  (-3.502,  -3.298) | -5.347  (-5.758,  -4.934) | -5.295  (-5.532,  -5.056) | -5.267  (-5.583,  -4.95) | -5.215  (-5.402,  -5.029) | -3.822  (-4.002,  -3.641) | -3.798  (-3.911,  -3.685) | -3.215  (-3.401,  -3.029) | -3.179  (-3.311,  -3.047) | -3.276  (-3.549,  -3.002) | -3.208  (-3.4,  -3.017) |
| 45-49 | -3.269  (-3.385,  -3.153) | -3.244  (-3.323,  -3.165) | -5.274  (-5.546,  -5) | -5.226  (-5.392,  -5.059) | -4.866  (-5.083,  -4.647) | -4.811  (-4.947,  -4.675) | -3.558  (-3.689,  -3.427) | -3.536  (-3.623,  -3.449) | -2.909  (-3.049,  -2.769) | -2.874  (-2.979,  -2.769) | -3.186  (-3.388,  -2.983) | -3.121  (-3.27,  -2.971) |
| 50-54 | -3.133  (-3.222,  -3.044) | -3.109  (-3.173,  -3.044) | -5.224  (-5.415,  -5.032) | -5.172  (-5.296,  -5.048) | -4.491  (-4.649,  -4.332) | -4.425  (-4.53,  -4.319) | -3.291  (-3.391,  -3.192) | -3.266  (-3.337,  -3.195) | -2.605  (-2.715,  -2.495) | -2.579  (-2.667,  -2.491) | -3.151  (-3.31,  -2.992) | -3.093  (-3.218,  -2.968) |

**Supplementary TABLE 12. (Continued)**

|  | Global | | High SDI ^c^ | | High-middle SDI ^c^ | | Middle SDI ^c^ | | Low-middle SDI ^c^ | | Low SDI ^c^ | |
| --- | --- | --- | --- | --- | --- | --- | --- | --- | --- | --- | --- | --- |
|  | Deaths rates | DALYs rates | Deaths rates | DALYs rates | Deaths rates | DALYs rates | Deaths rates | DALYs rate s | Deaths rates | DALYs rates | Deaths rates | DALYs rates |
| 55-59 | -2.998  (-3.07,  -2.926) | -2.977  (-3.033,  -2.921) | -5.261  (-5.403,  -5.12) | -5.197  (-5.296,  -5.099) | -4.05  (-4.173,  -3.927) | -3.983  (-4.071,  -3.896) | -3.086  (-3.167,  -3.005) | -3.063  (-3.125,  -3.001) | -2.365  (-2.456,  -2.275) | -2.347  (-2.425,  -2.269) | -3.045  (-3.176,  -2.915) | -3  (-3.11,  -2.891) |
| 60-64 | -2.85  (-2.911,  -2.789) | -2.833  (-2.885,  -2.782) | -5.457  (-5.568,  -5.346) | -5.382  (-5.466,  -5.299) | -3.668  (-3.768,  -3.568) | -3.61  (-3.687,  -3.533) | -2.845  (-2.914,  -2.775) | -2.83  (-2.887,  -2.772) | -2.204  (-2.283,  -2.124) | -2.192  (-2.266,  -2.118) | -2.772  (-2.884,  -2.659) | -2.739  (-2.841,  -2.636) |
| 65-69 | -2.659  (-2.713,  -2.604) | -2.648  (-2.698,  -2.598) | -5.654  (-5.745,  -5.562) | -5.581  (-5.656,  -5.506) | -3.286  (-3.37,  -3.201) | -3.244  (-3.315,  -3.172) | -2.571  (-2.634,  -2.509) | -2.555  (-2.612,  -2.499) | -2.037  (-2.111,  -1.962) | -2.037  (-2.113,  -1.96) | -2.386  (-2.489,  -2.283) | -2.365  (-2.469,  -2.262) |
| 70-74 | -2.384  (-2.436,  -2.332) | -2.378  (-2.431,  -2.326) | -5.755  (-5.833,  -5.676) | -5.7  (-5.771,  -5.628) | -2.85  (-2.928,  -2.773) | -2.816  (-2.889,  -2.743) | -2.164  (-2.224,  -2.104) | -2.147  (-2.207,  -2.087) | -1.788  (-1.863,  -1.713) | -1.8  (-1.886,  -1.715) | -1.851  (-1.955,  -1.748) | -1.847  (-1.962,  -1.732) |
| 75-79 | -2.082  (-2.135,  -2.029) | -2.076  (-2.136,  -2.016) | -5.643  (-5.714,  -5.571) | -5.6  (-5.672,  -5.529) | -2.412  (-2.487,  -2.337) | -2.4  (-2.479,  -2.321) | -1.653  (-1.715,  -1.591) | -1.642  (-1.712,  -1.572) | -1.469  (-1.552,  -1.387) | -1.491  (-1.596,  -1.387) | -1.315  (-1.43,  -1.198) | -1.342  (-1.485,  -1.198) |
| 80-84 | -1.807  (-1.864,  -1.75) | -1.804  (-1.877,  -1.732) | -5.272  (-5.339,  -5.205) | -5.253  (-5.329,  -5.178) | -1.889  (-1.965,  -1.813) | -1.905  (-1.995,  -1.815) | -1.129  (-1.19,  -1.058) | -1.129  (-1.218,  -1.039) | -1.079  (-1.178,  -0.979) | -1.104  (-1.244,  -0.964) | -0.903  (-1.048,  -0.757) | -0.959  (-1.158,  -0.759) |
| 85-89 | -1.675  (-1.746,  -1.604) | -1.669  (-1.768,  -1.57) | -4.757  (-4.828,  -4.685) | -4.756  (-4.844,  -4.668) | -1.598  (-1.692,  -1.505) | -1.619  (-1.741,  -1.498) | -0.752  (-0.844,  -0.66) | -0.75  (-0.878,  -0.622) | -0.761  (-0.897,  -0.624) | -0.79  (-1.001,  -0.578) | -0.597  (-0.809,  -0.385) | -0.666  (-0.987,  -0.344) |
| 90-94 | -1.789  (-1.895,  -1.682) | -1.781  (-1.941,  -1.621) | -4.353  (-4.443,  -4.262) | -4.358  (-4.479,  -4.237) | -1.514  (-1.659,  -1.369) | -1.539  (-1.742,  -1.335) | -0.733  (-0.882,  -0.584) | -0.733  (-0.953,  -0.511) | -0.624  (-0.848,  -0.399) | -0.653  (-1.025,  -0.279) | -0.384  (-0.752,  -0.014) | -0.454  (-1.054,  0.149) |
| 95+ | -2.173  (-2.375,  -1.97) | -2.177  (-2.493,  -1.861) | -4.231  (-4.379,  -4.083) | -4.25  (-4.457,  -4.042) | -1.798  (-2.097,  -1.498) | -1.841  (-2.274,  -1.405) | -0.983  (-1.294,  -0.671) | -0.999  (-1.477,  -0.519) | -0.633  (-1.096,  -0.167) | -0.672  (-1.469,  0.132) | -0.219  (-1.003,  0.572) | -0.292  (-1.61,  1.044) |
| Age group (male) Local drifts ^e^ (95% CI) ^f^ | | | | | | | | | | | | |
| 25-29 | -4.186  (-5.035,  -3.329) | -4.155  (-4.685,  -3.622) | -6.157  (-8.883,  -3.35) | -6.092  (-7.607,  -4.553) | -5.663  (-7.646,  -3.637) | -5.622  (-6.689,  -4.544) | -4.546  (-5.529,  -3.554) | -4.524  (-5.081,  -3.963) | -4.221  (-5.116,  -3.318) | -4.182  (-4.785,  -3.575) | -3.702  (-5.045,  -2.339) | -3.645  (-4.478,  -2.806) |

**Supplementary TABLE 12. (Continued)**

|  | Global | | High SDI ^c^ | | High-middle SDI ^c^ | | Middle SDI ^c^ | | Low-middle SDI ^c^ | | Low SDI ^c^ | |
| --- | --- | --- | --- | --- | --- | --- | --- | --- | --- | --- | --- | --- |
|  | Deaths rates | DALYs rates | Deaths rates | DALYs rates | Deaths rates | DALYs rates | Deaths rates | DALYs rate s | Deaths rates | DALYs rates | Deaths rates | DALYs rates |
| 30-34 | -3.938  (-4.336,  -3.538) | -3.898  (-4.154,  -3.641) | -5.713  (-6.819,  -4.594) | -5.652  (-6.283,  -5.018) | -5.502  (-6.313,  -4.683) | -5.451  (-5.899,  -5.001) | -4.282  (-4.735,  -3.828) | -4.248  (-4.512,  -3.983) | -3.82  (-4.251,  -3.386) | -3.774  (-4.075,  -3.473) | -3.502  (-4.191,  -2.809) | -3.425  (-3.865,  -2.982) |
| 5-39 | -3.634  (-3.885,  -3.383) | -3.591  (-3.758,  -3.423) | -5.553  (-6.152,  -4.95) | -5.488  (-5.842,  -5.133) | -5.396  (-5.88,  -4.91) | -5.34  (-5.616,  -5.063) | -3.935  (-4.217,  -3.652) | -3.894  (-4.065,  -3.722) | -3.39  (-3.669,  -3.111) | -3.345  (-3.546,  -3.143) | -3.234  (-3.681,  -2.784) | -3.149  (-3.447,  -2.85) |
| 40-44 | -3.34  (-3.513,  -3.167) | -3.301  (-3.422,  -3.18) | -5.458  (-5.825,  -5.09) | -5.399  (-5.626,  -5.171) | -5.173  (-5.492,  -4.852) | -5.123  (-5.314,  -4.932) | -3.614  (-3.81,  -3.417) | -3.578  (-3.703,  -3.453) | -2.954  (-3.15,  -2.757) | -2.909  (-3.058,  -2.759) | -2.995  (-3.306,  -2.683) | -2.916  (-3.133,  -2.697) |
| 45-49 | -3.186  (-3.313,  -3.058) | -3.147  (-3.242,  -3.053) | -5.384  (-5.629,  -5.139) | -5.333  (-5.493,  -5.174) | -4.776  (-4.998,  -4.553) | -4.725  (-4.866,  -4.584) | -3.333  (-3.477,  -3.189) | -3.301  (-3.398,  -3.204) | -2.671  (-2.82,  -2.522) | -2.627  (-2.747,  -2.507) | -2.945  (-3.176,  -2.714) | -2.871  (-3.042,  -2.7) |
| 50-54 | -3.034  (-3.132,  -2.936) | -3.002  (-3.08,  -2.925) | -5.311  (-5.484,  -5.138) | -5.262  (-5.381,  -5.142) | -4.418  (-4.582,  -4.254) | -4.36  (-4.47,  -4.249) | -3.03  (-3.141,  -2.919) | -3  (-3.08,  -2.92) | -2.371  (-2.489,  -2.253) | -2.341  (-2.442,  -2.24) | -2.949  (-3.131,  -2.766) | -2.886  (-3.029,  -2.743) |
| 55-59 | -2.909  (-2.989,  -2.829) | -2.88  (-2.948,  -2.812) | -5.313  (-5.443,  -5.184) | -5.257  (-5.353,  -5.16) | -3.985  (-4.114,  -3.855) | -3.923  (-4.016,  -3.829) | -2.826  (-2.918,  -2.735) | -2.797  (-2.868,  -2.727) | -2.147  (-2.245,  -2.048) | -2.126  (-2.217,  -2.035) | -2.89  (-3.041,  -2.738) | -2.84  (-2.967,  -2.713) |
| 60-64 | -2.773  (-2.842,  -2.703) | -2.753  (-2.817,  -2.689) | -5.481  (-5.584,  -5.378) | -5.417  (-5.5,  -5.334) | -3.617  (-3.725,  -3.508) | -3.568  (-3.653,  -3.483) | -2.595  (-2.675,  -2.514) | -2.576  (-2.643,  -2.509) | -1.949  (-2.037,  -1.861) | -1.942  (-2.03,  -1.854) | -2.599  (-2.731,  -2.467) | -2.566  (-2.686,  -2.446) |
| 65-69 | -2.641  (-2.704,  -2.578) | -2.633  (-2.696,  -2.569) | -5.692  (-5.779,  -5.605) | -5.634  (-5.711,  -5.557) | -3.284  (-3.38,  -3.189) | -3.256  (-3.338,  -3.174) | -2.344  (-2.418,  -2.271) | -2.332  (-2.399,  -2.264) | -1.79  (-1.874,  -1.706) | -1.799  (-1.891,  -1.707) | -2.237  (-2.359,  -2.114) | -2.221  (-2.343,  -2.099) |
| 70-74 | -2.429  (-2.491,  -2.367) | -2.432  (-2.501,  -2.362) | -5.859  (-5.937,  -5.782) | -5.819  (-5.895,  -5.743) | -2.891  (-2.983,  -2.799) | -2.869  (-2.957,  -2.781) | -1.964  (-2.036,  -1.891) | -1.961  (-2.035,  -1.887) | -1.54  (-1.627,  -1.453) | -1.567  (-1.673,  -1.462) | -1.697  (-1.822,  -1.571) | -1.708  (-1.847,  -1.569) |
| 75-79 | -2.127  (-2.193,  -2.062) | -2.14  (-2.222,  -2.059) | -5.764  (-5.838,  -5.69) | -5.741  (-5.821,  -5.661) | -2.455  (-2.549,  -2.36) | -2.457  (-2.557,  -2.356) | -1.443  (-1.521,  -1.365) | -1.46  (-1.549,  -1.371) | -1.179  (-1.277,  -1.081) | -1.217  (-1.349,  -1.085) | -1.118  (-1.26,  -0.974) | -1.172  (-1.349,  -0.996) |

**Supplementary TABLE 12. (Continued)**

|  | Global | | High SDI ^c^ | | High-middle SDI ^c^ | | Middle SDI ^c^ | | Low-middle SDI ^c^ | | Low SDI ^c^ | |
| --- | --- | --- | --- | --- | --- | --- | --- | --- | --- | --- | --- | --- |
|  | Deaths rates | DALYs rates | Deaths rates | DALYs rates | Deaths rates | DALYs rates | Deaths rates | DALYs rate s | Deaths rates | DALYs rates | Deaths rates | DALYs rates |
| 80-84 | -1.771  (-1.846,  -1.696) | -1.801  (-1.904,  -1.697) | -5.367  (-5.441,  -5.292) | -5.372  (-5.461,  -5.283) | -1.825  (-1.927,  -1.722) | -1.866  (-1.988,  -1.743) | -0.829  (-0.921,  -0.736) | -0.871  (-0.989,  -0.752) | -0.746  (-0.865,  -0.626) | -0.791  (-0.971,  -0.612) | -0.594  (-0.776,  -0.412) | -0.686  (-0.936,  -0.437) |
| 85-89 | -1.475  (-1.573,  -1.377) | -1.508  (-1.658,  -1.357) | -4.767  (-4.853,  -4.681) | -4.792  (-4.906,  -4.679) | -1.381  (-1.516,  -1.246) | -1.43  (-1.608,  -1.252) | -0.305  (-0.429,  -0.18) | -0.343  (-0.518,  -0.167) | -0.339  (-0.505,  -0.173) | -0.393  (-0.666,  -0.118) | -0.14  (-0.412,  0.133) | -0.25  (-0.661,  0.163) |
| 90-94 | -1.452  (-1.611,  -1.294) | -1.48  (-1.74,  -1.22) | -4.264  (-4.386,  -4.142) | -4.296  (-4.471,  -4.12) | -1.185  (-1.413,  -0.957) | -1.23  (-1.552,  -0.907) | -0.287  (-0.493,  -0.08) | -0.316  (-0.626,  -0.005) | -0.134  (-0.409,  0.141) | -0.187  (-0.673,  0.302) | 0.171  (-0.319,  0.663) | 0.062  (-0.734,  0.864) |
| 95+ | -1.515  (-1.551,  -1.479) | -1.885  (-2.449,  -1.318) | -4.126  (-4.351,  -3.901) | -4.171  (-4.511,  -3.829) | -1.561  (-2.082,  -1.037) | -1.62  (-2.385,  -0.849) | -0.925  (-1.38,  -0.467) | -0.964  (-1.671,  -0.251) | -0.143  (-0.726,  0.444) | -0.201  (-1.27,  0.879) | 0.389  (-0.721,  1.512) | 0.283  (-1.574,  2.174) |
| Age group (female) Local drifts ^e^ (95% CI) ^f^ | | | | | | | | | | | | |
| 25-29 | -4.9  (-5.96,  -3.827) | -4.918  (-5.48,  -4.352) | -6.443  (-10.9,  -1.763) | -6.374  (-8.667,  -4.024) | -6.917  (-10.066,  -3.658) | -6.84  (-8.46,  -5.193) | -5.579  (-7.088,  -4.045) | -5.601  (-6.409,  -4.785) | -5.396  (-6.463,  -4.317) | -5.393  (-6.023,  -4.758) | -4.935  (-6.054,  -3.804) | -4.922  (-5.634,  -4.205) |
| 30-34 | -4.528  (-5.046,  -4.008) | -4.537  (-4.819,  -4.254) | -5.864  (-7.751,  -3.939) | -5.81  (-6.802,  -4.808) | -6.503  (-7.788,  -5.2) | -6.41  (-7.085,  -5.729) | -5.318  (-6.032,  -4.599) | -5.317  (-5.709,  -4.923) | -4.857  (-5.389,  -4.321) | -4.854  (-5.177,  -4.53) | -4.484  (-5.063,  -3.901) | -4.449  (-4.83,  -4.068) |
| 35-39 | -4.019  (-4.351,  -3.685) | -4.018  (-4.205,  -3.829) | -5.454  (-6.498,  -4.399) | -5.403  (-5.972,  -4.831) | -6.159  (-6.917,  -5.396) | -6.059  (-6.47,  -5.645) | -4.794  (-5.241,  -4.344) | -4.782  (-5.037,  -4.527) | -4.215  (-4.562,  -3.867) | -4.204  (-4.423,  -3.985) | -4.12  (-4.512,  -3.726) | -4.071  (-4.338,  -3.803) |
| 40-44 | -3.606  (-3.837,  -3.375) | -3.608  (-3.744,  -3.471) | -5.116  (-5.747,  -4.481) | -5.07  (-5.43,  -4.708) | -5.754  (-6.247,  -5.259) | -5.671  (-5.951,  -5.39) | -4.322  (-4.625,  -4.017) | -4.322  (-4.503,  -4.141) | -3.697  (-3.943,  -3.451) | -3.683  (-3.845,  -3.519) | -3.85  (-4.132,  -3.566) | -3.797  (-3.999,  -3.595) |
| 45-49 | -3.395  (-3.561,  -3.229) | -3.4  (-3.504,  -3.296) | -4.97  (-5.377,  -4.561) | -4.918  (-5.163,  -4.673) | -5.209  (-5.537,  -4.88) | -5.125  (-5.322,  -4.928) | -4.011  (-4.225,  -3.797) | -4.017  (-4.152,  -3.882) | -3.28  (-3.462,  -3.098) | -3.272  (-3.4,  -3.144) | -3.662  (-3.872,  -3.452) | -3.612  (-3.77,  -3.454) |
| 50-54 | -3.293  (-3.418,  -3.168) | -3.289  (-3.373,  -3.206) | -4.983  (-5.259,  -4.707) | -4.909  (-5.085,  -4.732) | -4.773  (-5.002,  -4.544) | -4.673  (-4.819,  -4.527) | -3.785  (-3.944,  -3.627) | -3.778  (-3.884,  -3.671) | -2.975  (-3.116,  -2.834) | -2.966  (-3.071,  -2.861) | -3.522  (-3.684,  -3.359) | -3.475  (-3.605,  -3.345) |

**Supplementary TABLE 12. (Continued)**

|  | Global | | High SDI ^c^ | | High-middle SDI ^c^ | | Middle SDI ^c^ | | Low-middle SDI ^c^ | | Low SDI ^c^ | |
| --- | --- | --- | --- | --- | --- | --- | --- | --- | --- | --- | --- | --- |
|  | Deaths rates | DALYs rates | Deaths rates | DALYs rates | Deaths rates | DALYs rates | Deaths rates | DALYs rate s | Deaths rates | DALYs rates | Deaths rates | DALYs rates |
| 55-59 | -3.134  (-3.231,  -3.037) | -3.137  (-3.206,  -3.067) | -5.183  (-5.379,  -4.987) | -5.082  (-5.215,  -4.948) | -4.298  (-4.464,  -4.132) | -4.207  (-4.32,  -4.09) | -3.542  (-3.664,  -3.419) | -3.538  (-3.627,  -3.449) | -2.701  (-2.813,  -2.588) | -2.696  (-2.787,  -2.606) | -3.279  (-3.409,  -3.149) | -3.247  (-3.359,  -3.135) |
| 60-64 | -3.01  (-3.089,  -2.93) | -3.004  (-3.066,  -2.943) | -5.577  (-5.723,  -5.431) | -5.456  (-5.563,  -5.349) | -3.911  (-4.038,  -3.784) | -3.83  (-3.924,  -3.737) | -3.294  (-3.396,  -3.193) | -3.289  (-3.368,  -3.21) | -2.586  (-2.682,  -2.49) | -2.572  (-2.656,  -2.489) | -2.986  (-3.096,  -2.876) | -2.955  (-3.058,  -2.853) |
| 65-69 | -2.776  (-2.843,  -2.708) | -2.765  (-2.822,  -2.708) | -5.931  (-6.043,  -5.819) | -5.811  (-5.9,  -5.722) | -3.475  (-3.574,  -3.377) | -3.409  (-3.489,  -3.33) | -2.989  (-3.075,  -2.902) | -2.97  (-3.044,  -2.896) | -2.36  (-2.446,  -2.273) | -2.346  (-2.429,  -2.264) | -2.528  (-2.626,  -2.429) | -2.5  (-2.601,  -2.399) |
| 70-74 | -2.458  (-2.519,  -2.397) | -2.439  (-2.497,  -2.382) | -6.059  (-6.149,  -5.97) | -5.963  (-6.041,  -5.885) | -3.018  (-3.1,  -2.935) | -2.961  (-3.035,  -2.886) | -2.536  (-2.615,  -2.457) | -2.5  (-2.575,  -2.425) | -2.067  (-2.151,  -1.983) | -2.053  (-2.142,  -1.964) | -2  (-2.095,  -1.904) | -1.972  (-2.082,  -1.863) |
| 75-79 | -2.183  (-2.242,  -2.125) | -2.152  (-2.214,  -2.091) | -5.941  (-6.015,  -5.866) | -5.867  (-5.939,  -5.794) | -2.59  (-2.664,  -2.515) | -2.561  (-2.635,  -2.486) | -2.021  (-2.098,  -1.943) | -1.976  (-2.058,  -1.893) | -1.751  (-1.84,  -1.662) | -1.746  (-1.851,  -1.64) | -1.518  (-1.623,  -1.414) | -1.508  (-1.641,  -1.376) |
| 80-84 | -2  (-2.06,  -1.94) | -1.964  (-2.034,  -1.894) | -5.575  (-5.637,  -5.512) | -5.535  (-5.604,  -5.466) | -2.131  (-2.201,  -2.062) | -2.131  (-2.21,  -2.052) | -1.542  (-1.626,  -1.458) | -1.495  (-1.596,  -1.394) | -1.354  (-1.459,  -1.249) | -1.351  (-1.49,  -1.212) | -1.241  (-1.368,  -1.114) | -1.253  (-1.431,  -1.073) |
| 85-89 | -2.004  (-2.074,  -1.933) | -1.958  (-2.049,  -1.867) | -5.064  (-5.124,  -5.004) | -5.051  (-5.125,  -4.977) | -1.923  (-2.003,  -1.843) | -1.928  (-2.029,  -1.826) | -1.225  (-1.33,  -1.121) | -1.175  (-1.313,  -1.036) | -1.106  (-1.248,  -0.964) | -1.102  (-1.308,  -0.895) | -1.127  (-1.306,  -0.946) | -1.148  (-1.428,  -0.867) |
| 90-94 | -2.183  (-2.284,  -2.083) | -2.139  (-2.277,  -2.0) | -4.647  (-4.717,  -4.577) | -4.648  (-4.741,  -4.555) | -1.834  (-1.952,  -1.715) | -1.851  (-2.011,  -1.69) | -1.109  (-1.274,  -0.945) | -1.067  (-1.3,  -0.833) | -1.028  (-1.259,  -0.797) | -1.027  (-1.386,  -0.667) | -1.043  (-1.345,  -0.741) | -1.069  (-1.573,  -0.562) |
| 95+ | -2.498  (-2.678,  -2.318) | -2.464  (-2.723,  -2.205) | -4.453  (-4.56,  -4.346) | -4.471  (-4.619,  -4.323) | -2.001  (-2.232,  -1.77) | -2.035  (-2.36,  -1.71) | -1.016  (-1.345,  -0.685) | -0.996  (-1.48,  -0.51) | -1.001  (-1.463,  -0.536) | -1.014  (-1.762,  -0.261) | -0.919  (-1.526,  -0.308) | -0.957  (-2.005,  0.103) |

**Note:** ^a^ IHD = ischemic heart disease;

^b^ DALYs = disability adjusted life years.

^c^ SDI = socio-demographic index.

^d^ Net drifts represent the overall annual percentage change in the age-standardized rate based on period and birth cohort.

^e^ Local drifts indicate the annual percentage change over time specific to the age group.

^f^ CI = confidence interval. All net and local drifts were statistically significant (*P*<0.05).

**Supplementary FIGURE 1** Age-standardized deaths and DALYs rates (per 100,000 population) of IHD attributed to overall environmental risk factors in 2021stratified by country for male.

(A) Age-standardized IHD deaths rates attributed to overall environmental factors for male. (B) Age-standardized IHD DALYs rates attributed to overall environmental factors for male. (C) Age-standardized IHD deaths rates attributed to particulate matter pollution for male. (D) Age-standardized IHD DALYs rates attributed to particulate matter pollution for male. (E) Age-standardized IHD deaths rates attributed to non-optimal temperature for male. (F) Age-standardized IHD DALYs rates attributed to non-optimal temperature for male. (G) Age-standardized IHD deaths rates attributed to lead exposure for male. (H) Age-standardized IHD DALYs rates attributed to lead exposure for male.

**Supplementary FIGURE 2** Age-standardized deaths and DALYs rates (per 100,000 population) of IHD attributed to overall environmental risk factors in 2021stratified by country for female.

(A) Age-standardized IHD deaths rates attributed to overall environmental factors for female. (B) Age-standardized IHD DALYs rates attributed to overall environmental factors for female. (C) Age-standardized IHD deaths rates attributed to particulate matter pollution for female. (D) Age-standardized IHD DALYs rates attributed to particulate matter pollution for female. (E) Age-standardized IHD deaths rates attributed to non-optimal temperature for female. (F) Age-standardized IHD DALYs rates attributed to non-optimal temperature for female. (G) Age-standardized IHD deaths rates attributed to lead exposure for female. (H) Age-standardized IHD DALYs rates attributed to lead exposure for female.

**Supplementary FIGURE 3** Age, period, and cohort effects on IHD deaths and DALYs rates attributed to Overall Environmental for male.

(A) Age-specific IHD Deaths rates attributed to Overall Environmental by SDI regions for male. (B) Age-specific IHD DALYs rates attributed to Overall Environmental by SDI regions for male. (C) Period -specific IHD Deaths rates attributed to Overall Environmental by SDI regions for male. (D) Period -specific IHD DALYs rates attributed to Overall Environmental by SDI regions for male. (E) Cohort-specific IHD Deaths rates attributed to Overall Environmental by SDI regions for male. (F) Cohort-specific IHD DALYs rates attributed to Overall Environmental by SDI regions for male.

**Supplementary FIGURE 4** Age, period, and cohort effects on IHD deaths and DALYs rates attributed to Overall Environmental for female.

(A) Age-specific IHD Deaths rates attributed to Overall Environmental by SDI regions for female. (B) Age-specific IHD DALYs rates attributed to Overall Environmental by SDI regions for female. (C) Period -specific IHD Deaths rates attributed to Overall Environmental by SDI regions for female. (D) Period -specific IHD DALYs rates attributed to Overall Environmental by SDI regions for female. (E) Cohort-specific IHD Deaths rates attributed to Overall Environmental by SDI regions for female. (F) Cohort-specific IHD DALYs rates attributed to Overall Environmental by SDI regions for female.

**Supplementary FIGURE 5** Age, period, and cohort effects on IHD deaths and DALYs rates attributed to particulate matter pollution for male.

(A) Age-specific IHD Deaths rates attributed to particulate matter pollution by SDI regions for male. (B) Age-specific IHD DALYs rates attributed to particulate matter pollution by SDI regions for male. (C) Period -specific IHD Deaths rates attributed to particulate matter pollution by SDI regions for male. (D) Period -specific IHD DALYs rates attributed to particulate matter pollution by SDI regions for male. (E) Cohort-specific IHD Deaths rates attributed to particulate matter pollution by SDI regions for male. (F) Cohort-specific IHD DALYs rates attributed to particulate matter pollution by SDI regions for male.

**Supplementary FIGURE 6** Age, period, and cohort effects on IHD deaths and DALYs rates attributed to particulate matter pollution for female.

(A) Age-specific IHD Deaths rates attributed to particulate matter pollution by SDI regions for female. (B) Age-specific IHD DALYs rates attributed to particulate matter pollution by SDI regions for female. (C) Period -specific IHD Deaths rates attributed to particulate matter pollution by SDI regions for female. (D) Period -specific IHD DALYs rates attributed to particulate matter pollution by SDI regions for female. (E) Cohort-specific IHD Deaths rates attributed to particulate matter pollution by SDI regions for female. (F) Cohort-specific IHD DALYs rates attributed to particulate matter pollution by SDI regions for female.

**Supplementary FIGURE 7** Age, period, and cohort effects on IHD deaths and DALYs rates attributed to non-optimal temperature for male.

(A) Age-specific IHD Deaths rates attributed to non-optimal temperature by SDI regions for male. (B) Age-specific IHD DALYs rates attributed to non-optimal temperature by SDI regions for male. (C) Period -specific IHD Deaths rates attributed to non-optimal temperature by SDI regions for male. (D) Period -specific IHD DALYs rates attributed to non-optimal temperature by SDI regions for male. (E) Cohort-specific IHD Deaths rates attributed to non-optimal temperature by SDI regions for male. (F) Cohort-specific IHD DALYs rates attributed to non-optimal temperature by SDI regions for male.

**Supplementary FIGURE 8** Age, period, and cohort effects on IHD deaths and DALYs rates attributed to non-optimal temperature for female.

(A) Age-specific IHD Deaths rates attributed to non-optimal temperature by SDI regions for female. (B) Age-specific IHD DALYs rates attributed to non-optimal temperature by SDI regions for female. (C) Period -specific IHD Deaths rates attributed to non-optimal temperature by SDI regions for female. (D) Period -specific IHD DALYs rates attributed to non-optimal temperature by SDI regions for female. (E) Cohort-specific IHD Deaths rates attributed to non-optimal temperature by SDI regions for female. (F) Cohort-specific IHD DALYs rates attributed to non-optimal temperature by SDI regions for female.

**Supplementary FIGURE 9** Age, period, and cohort effects on IHD deaths and DALYs rates attributed to lead exposure for male.

(A) Age-specific IHD Deaths rates attributed to Lead exposure by SDI regions for male. (B) Age-specific IHD DALYs rates attributed to Lead exposure by SDI regions for male. (C) Period -specific IHD Deaths rates attributed to Lead exposure by SDI regions for male. (D) Period -specific IHD DALYs rates attributed to Lead exposure by SDI regions for male. (E) Cohort-specific IHD Deaths rates attributed to Lead exposure by SDI regions for male. (F) Cohort-specific IHD DALYs rates attributed to Lead exposure by SDI regions for male.

**Supplementary FIGURE 10** Age, period, and cohort effects on IHD deaths and DALYs rates attributed to lead exposure for female.

(A) Age-specific IHD Deaths rates attributed to Lead exposure by SDI regions for female. (B) Age-specific IHD DALYs rates attributed to Lead exposure by SDI regions for female. (C) Period -specific IHD Deaths rates attributed to Lead exposure by SDI regions for female. (D) Period -specific IHD DALYs rates attributed to Lead exposure by SDI regions for female. (E) Cohort-specific IHD Deaths rates attributed to Lead exposure by SDI regions for female. (F) Cohort-specific IHD DALYs rates attributed to Lead exposure by SDI regions for female.
